# Supplementary material for: Reconstruction of stochastic temporal networks through diffusive arrival times
Source: Nat Commun. 2017 Jun 12;8:15729. doi: 10.1038/ncomms15729 (PMC5472785; doi:10.1038/ncomms15729)
Supplement: Supplementary Information — Supplementary Figures, Supplementary Tables Supplementary Notes and Supplementary References. [file ncomms15729-s1.pdf]

Supplementary Figures

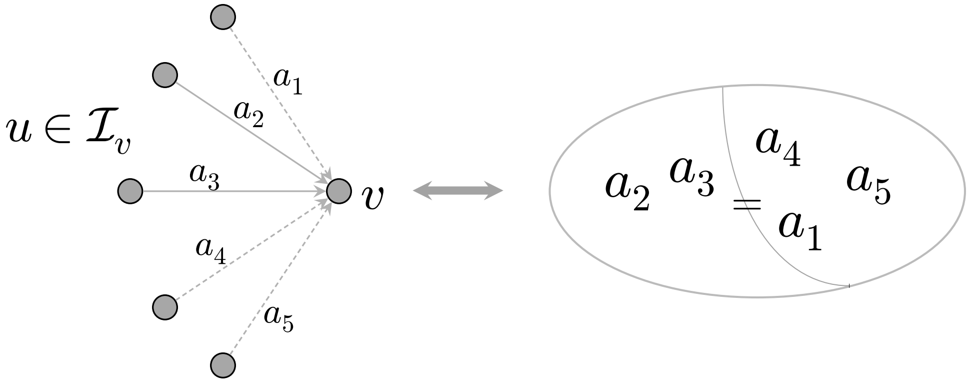

Supplementary Figure 1: Reduction of the local topology inference to the NP-hard PARTITION problem. This illustrative example of the ML local configuration $\mathcal{I}_v$ consists of two links (solid arrows) incident to the focus node v, which corresponds to a possible partition for achieving the PARTITION task (see Supplementary Note 3).

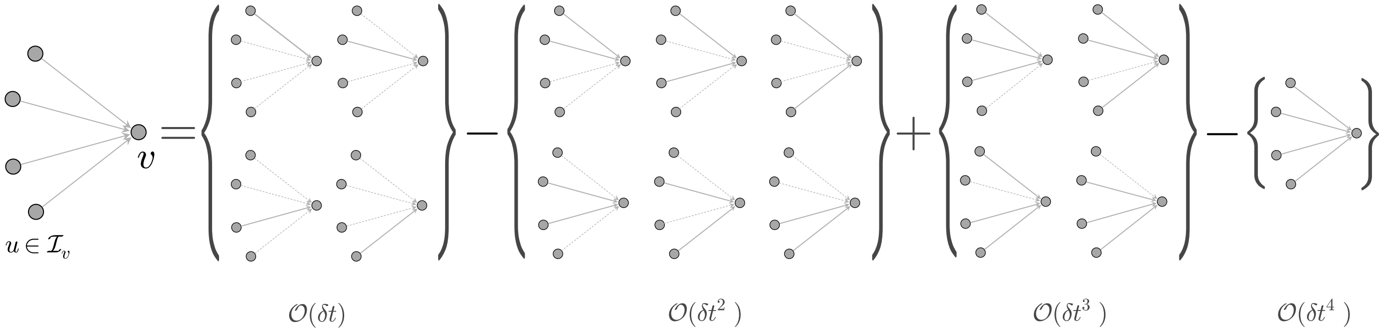

Supplementary Figure 2: An illustrative example of local diffusion structure permitting tied branches. Within a local configuration $\mathcal{I}_v$, the (in-)degree of the focus node is 4. Diffusion branches (solid arrows) correspond to exactly having waiting times $\tau_{uv} = d_{uv}$ with probability $\rho(d_{uv})\delta t$, and chords (dashed arrows) to having censored waiting times $\tau_{uv} \geq d_{uv}$ with probability $\Phi(d_{uv})$. Therefore the probability of observing DATs $\mathcal{D}$ is obtained from the inclusion-exclusion principle [see equation (53) in Supplementary Note 10].

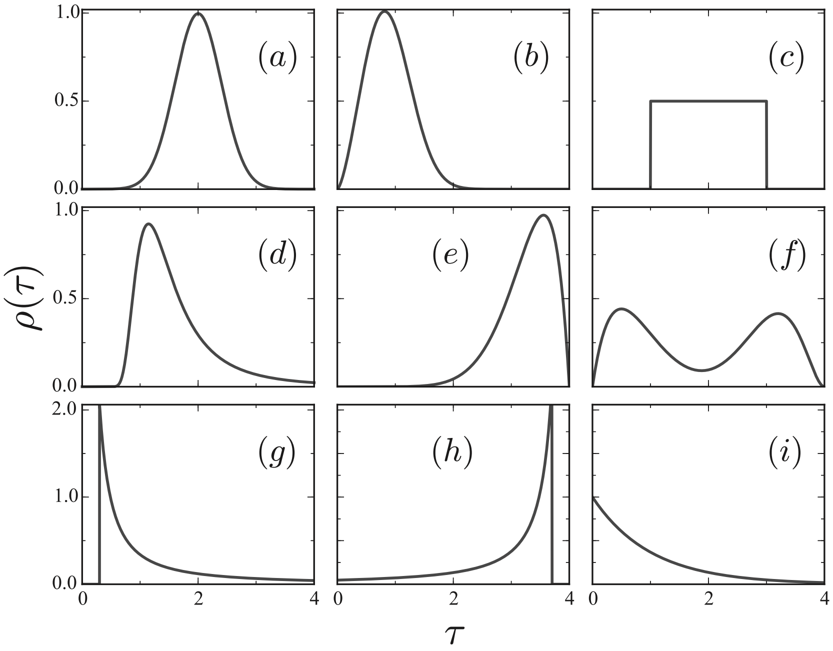

Supplementary Figure 3: Benchmark WTDs used in numerical experiments. See Supplementary Table 3 for distributional parameters and more detailed descriptions.

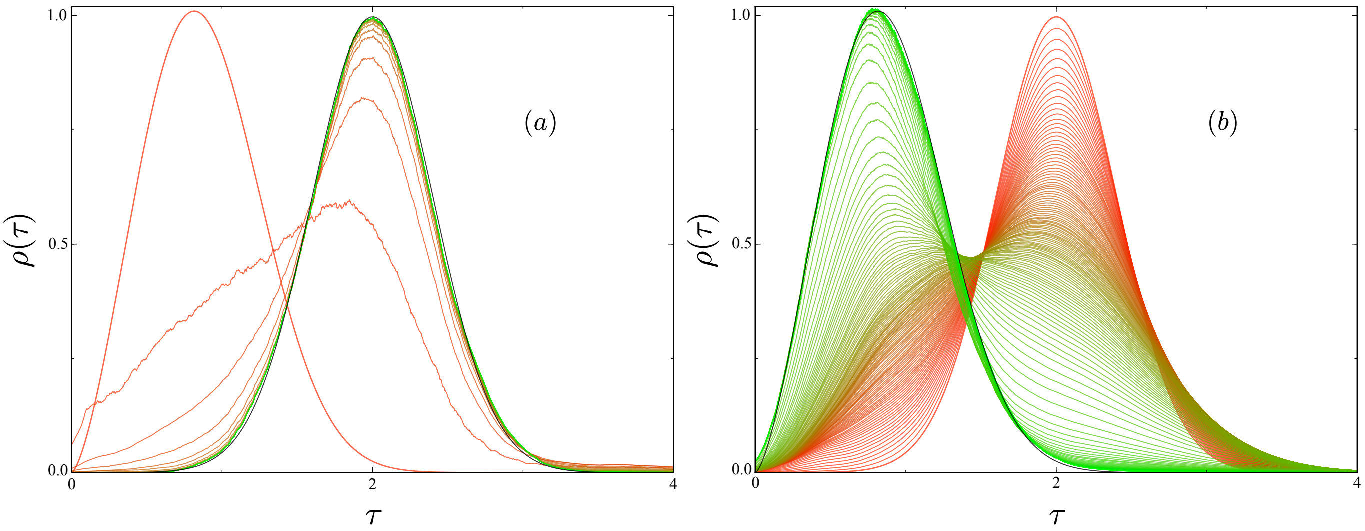

Supplementary Figure 4: Effects of initial condition $\hat{\rho}^0$ on the iterative convergence rate. (a) The real WTD ρ (black line) is Gaussian of mean $\mu = 2$ and variance $\sigma^2 = 0.4^2$), and the *a priori* guess $\hat{\rho}^0$ (red line) is Weibull with scale $a = 1$ and exponent $b = 2.5$; (b) the real and the *a priori* WTDs interchange their roles. Here we assume $\rho_{uv}(\tau)$ to be identical for all links $(u, v) \in \mathcal{G}$. The iterative procedure converges to the correct WTD estimation $\hat{\rho}$ (green line) after 17 and 347 iterations (gradient-coloured lines) for both under- and overestimated cases, respectively. The underlying time-aggregated network is an Erdös-Rényi (ER) random graph consisting of $N = 100$ nodes and $L = 291$ links. Other parameters are set as: relative sample size $C/N = 1$, kernel bandwidth $h = 0.05$, WTD support $l_\tau = 8$, and error threshold $\epsilon = 10^{-4}$.

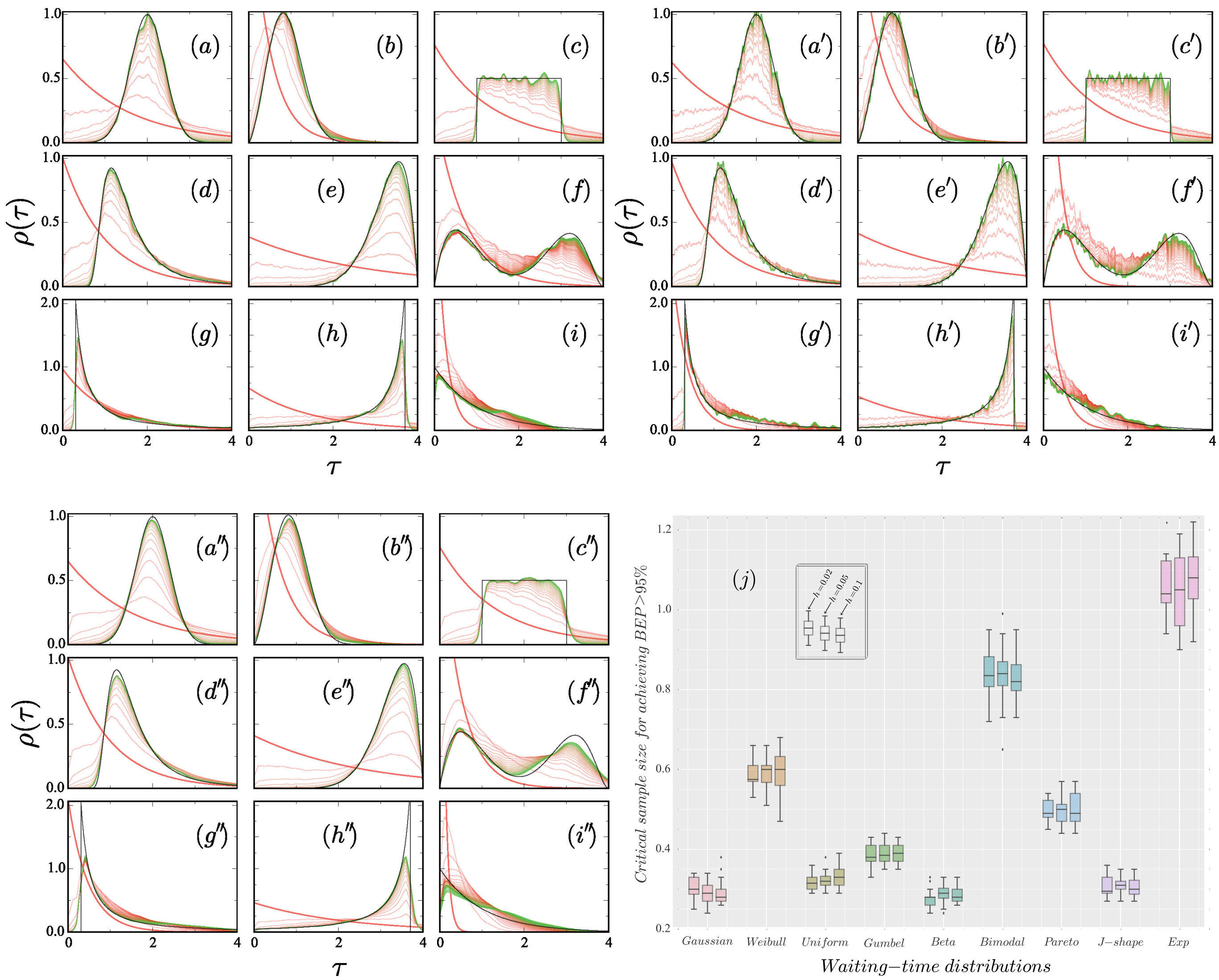

Supplementary Figure 5: Effects of kernel bandwidth h on the estimation performance. Illustrative examples are provided for estimating different WTDs based on samples of diffusion cascades D of relative size $C/N = 1$. The kernel bandwidths are (a)-(i) $h = 0.05$, (a')-(i') $h = 0.02$, and (a'')-(i'') $h = 0.1$. (j) Box plot of critical sample sizes that achieve at least 0.95 BEP for reconstruction of underlying networks with different kernel bandwidths. Each box plot is obtained by using 10 independent realisations. The real WTDs are listed in Supplementary Table 3 and *a priori* WTDs are selected as exponentials of mean $\mu_0 = \left\langle \min_{v\in\mathcal{V}\setminus s^*} t_v \right\rangle_D$ (see Methods). The underlying network and other parameters are the same as Supplementary Figure 4.

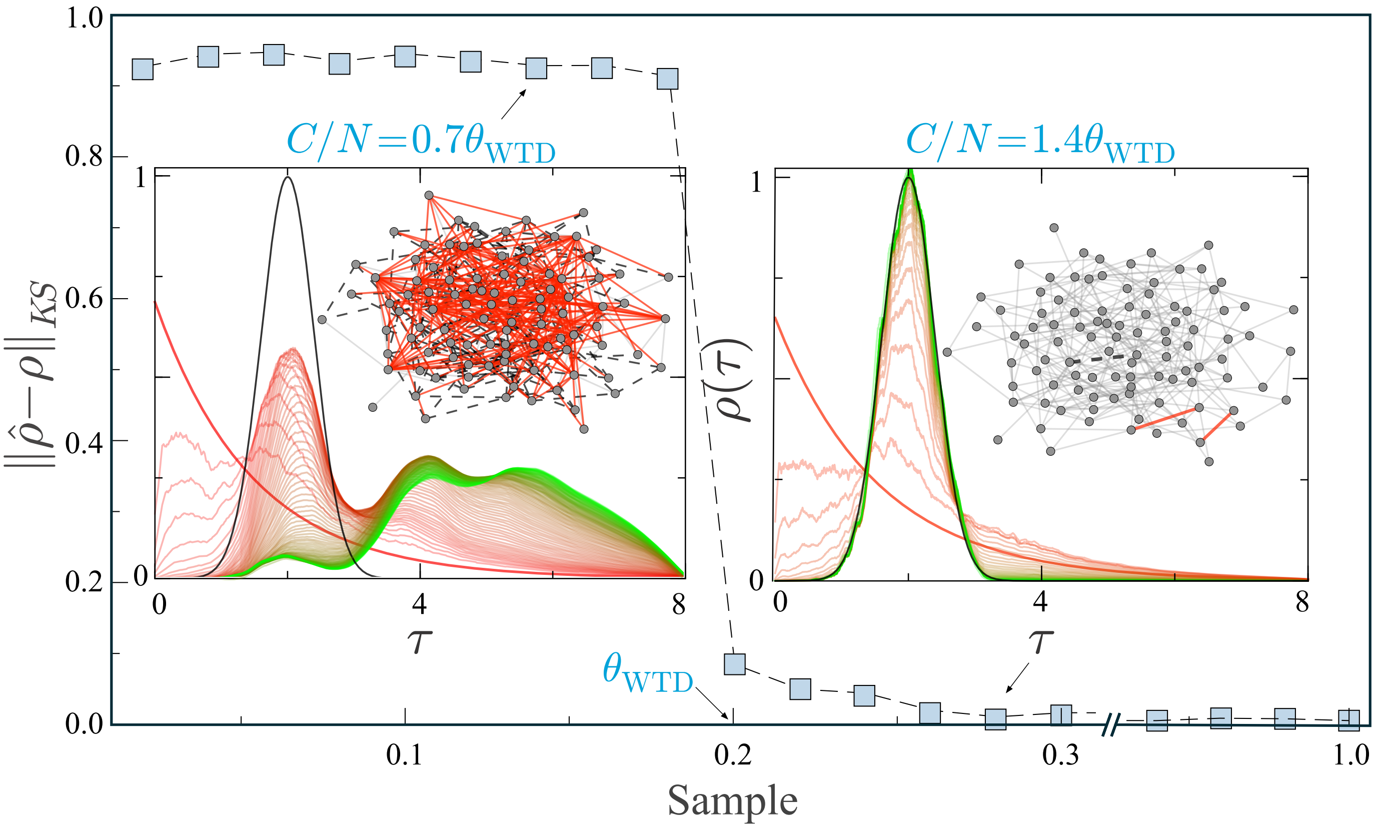

Supplementary Figure 6: Phase transition observed in WTD recovery. Kolmogorov-Smirnov divergence $\|\rho,\hat{\rho}\|_{KS}$ as an order parameter are plotted versus the relative sample size C/N. Inset: the iteratively estimated WTDs (gradient-coloured lines) as well as the optimal configuration of time-aggregated graph (in the sense of attaining BEP between precision and recall of existing links, see Methods) simultaneously obtained using synthesised diffusion cascades of (a) a subcritical sample size $C/N = 0.7\theta_{\mathrm{WTD}}$, and (b) a supercritical sample size $C/N = 1.4\theta_{\mathrm{WTD}}$, respectively. Real WTDs represented by black lines are adopted as Gaussian of mean $\mu = 2$ and variance $\sigma^2 = 0.4^2$, the time-aggregated network is the ER random network of size $N = 100$ and average degree $\langle k \rangle = 6$, and erroneous presence and erroneous absence of links are respectively represented by red solid and black dashed lines. Other parameters: kernel bandwidth $h = 0.05$, WTD support $l_\tau = 8$, and error threshold $\epsilon = 10^{-4}$. The critical sample size θ_{WTD} is defined as the minimal C/N assuring a sufficiently small distributional divergence between the estimated and real WTDs. Here we adopted $\|\rho,\hat{\rho}\|_{KS} < 0.1$.

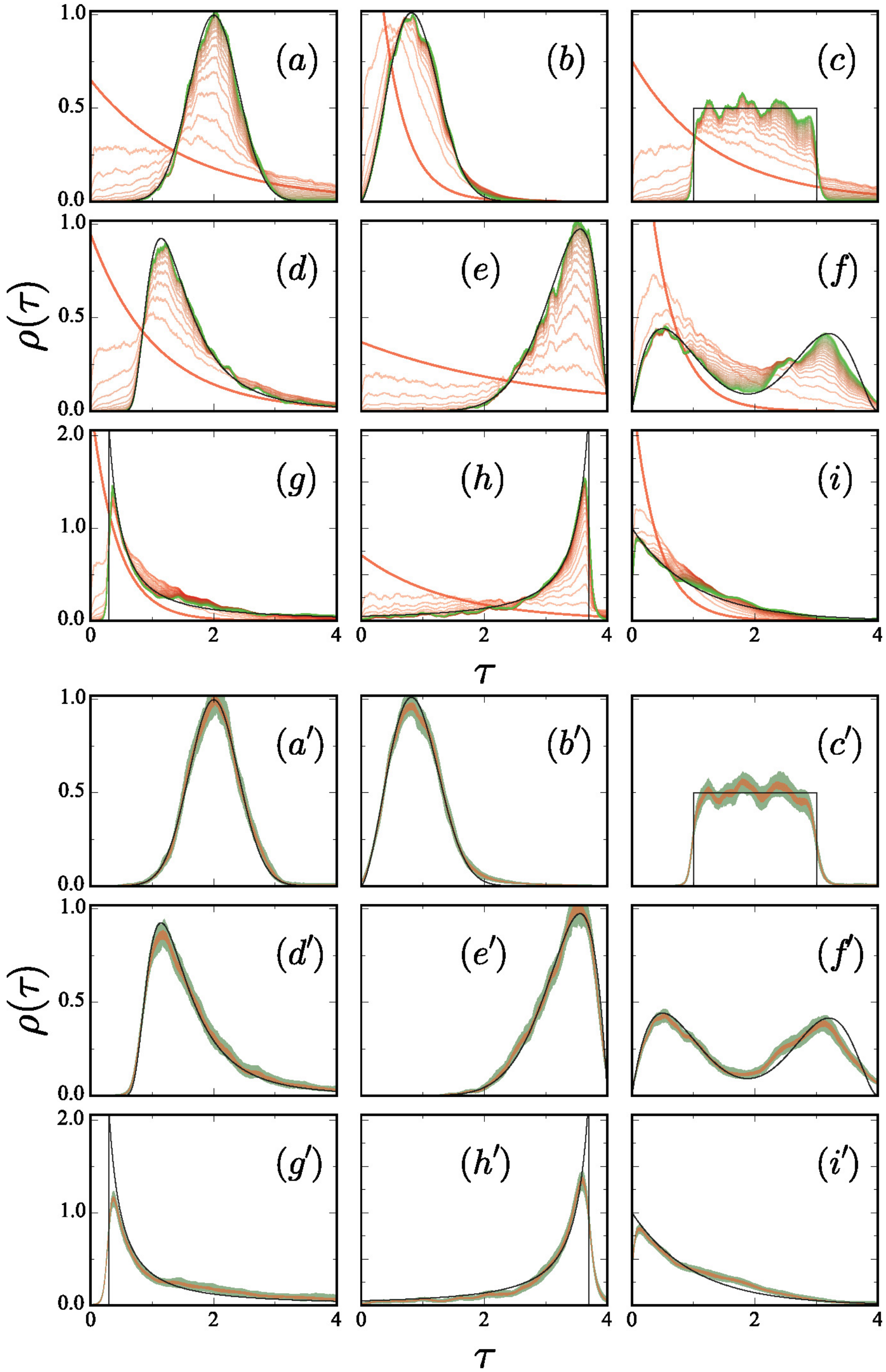

Supplementary Figure 7: WTD estimates with confidence bands using parametric bootstrap method. (a)-(i) The evolving WTDs during self-consistency iterations (gradient-colored lines) using supercritical samples of size $C/N = 1.4\theta_{\mathrm{WTD}}$ (see Supplementary Table 5) relative to network size, ultimately converging to the true underlying WTDs (black lines). (a')-(i') The 50% and 95% bootstrap confidence bands for different WTD estimates obtained using Algorithm 7. The time-aggregated network is ER random network with size $N = 100$ and average degree $\langle k \rangle = 5.8$. Other parameters: bootstrap size $B = 100$, kernel bandwidth $h = 0.05$.

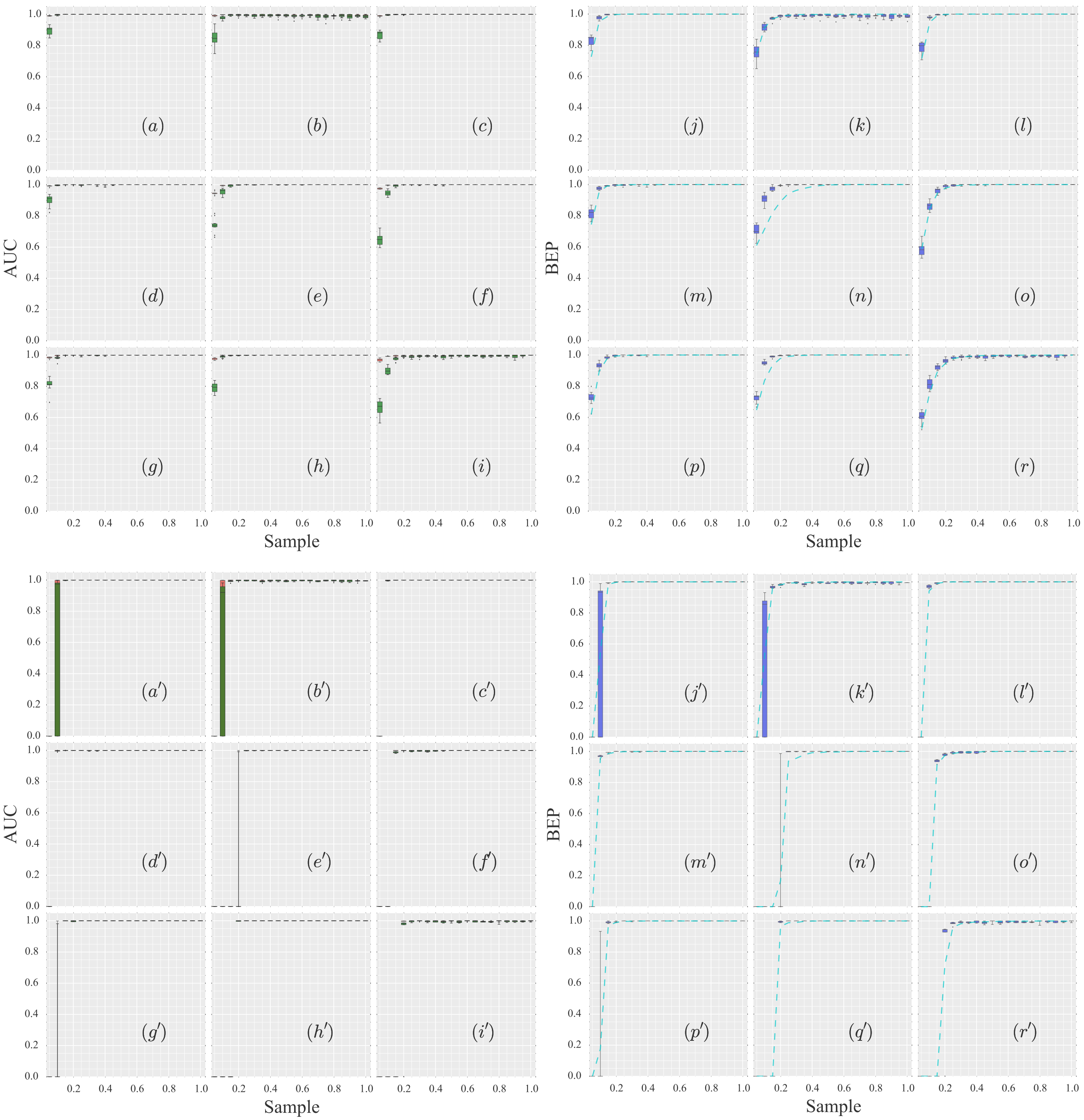

Supplementary Figure 8: Box plot of AUC and BEP of reconstructing Lattice network. (a)-(i) AUROC (red box plots) and AUPR (green box plots), and (j)-(r) the real (blue box plots) and estimated BEP (dashed lines) of topology inference results in the case of explicit WTDs; (a')-(r') the same measurement indices for iteratively estimated topologies without *a priori* knowledge of associated WTDs. The benchmark WTDs are listed in Supplementary Table 3. Other parameters: network size $N = 100$, average degree $\langle k \rangle = 3.6$, hyperparameter $\beta = 0$, kernel bandwidth $h = 0.05$, WTD support $l_\tau = 8$, and error threshold $\epsilon = 10^{-4}$. Each box plot is obtained by using 10 independent realizations.

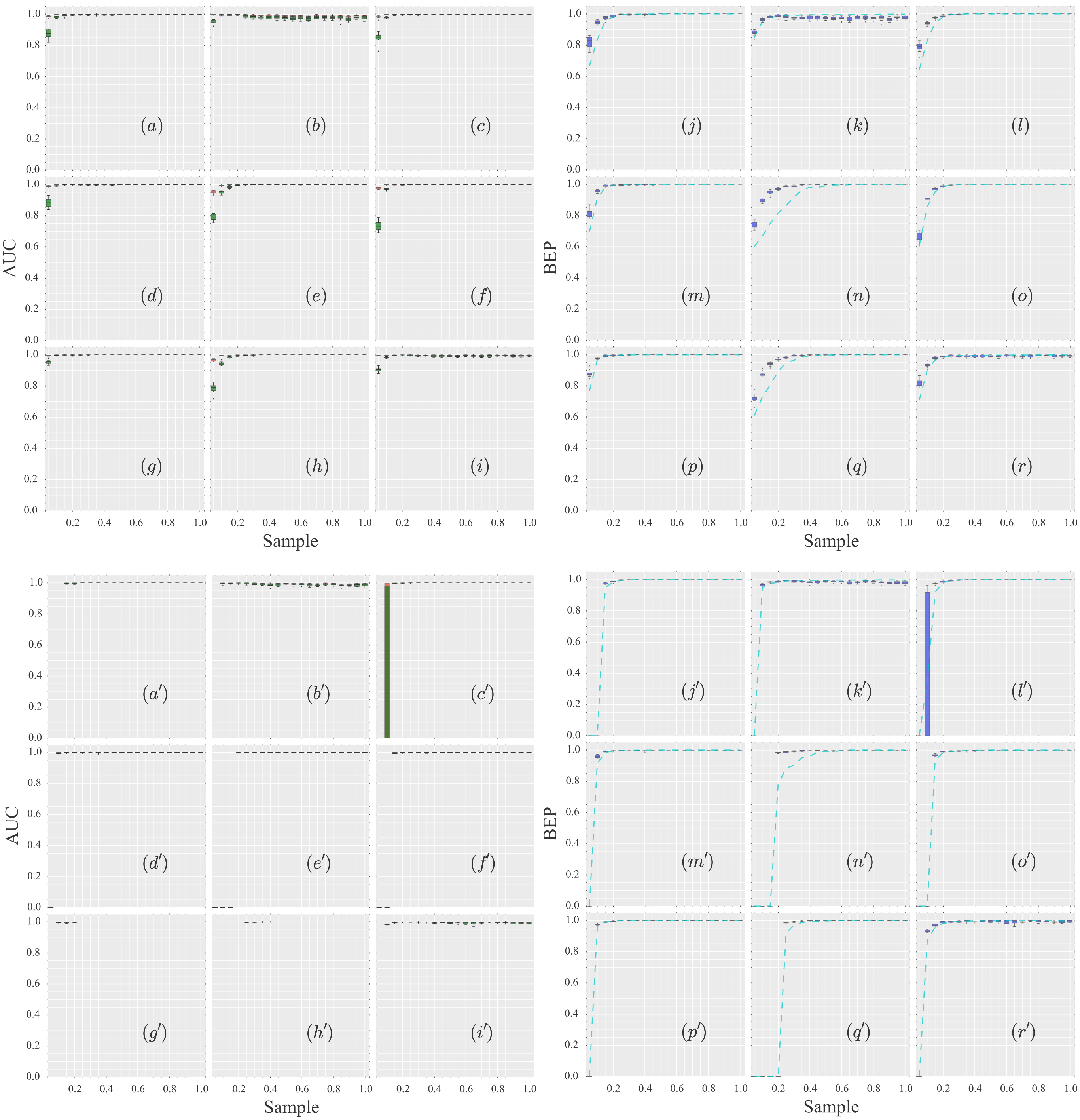

Supplementary Figure 9: Box plot of AUC and BEP of reconstructing Sierpinski network. (a)-(i) AUROC (red box plots) and AUPR (green box plots), and (j)-(r) the real (blue box plots) and estimated BEP (dashed lines) of topology inference results in the case of explicit WTDs; (a')-(r') the same measurement indices for iteratively estimated topologies without *a priori* knowledge of associated WTDs. The benchmark WTDs are listed in Supplementary Table 3. Other parameters: network size $N = 123$, average degree $\langle k \rangle = 4.0$, hyperparameter $\beta = 0$, kernel bandwidth $h = 0.05$, WTD support $l_\tau = 8$, and error threshold $\epsilon = 10^{-4}$. Each box plot is obtained by using 10 independent realizations.

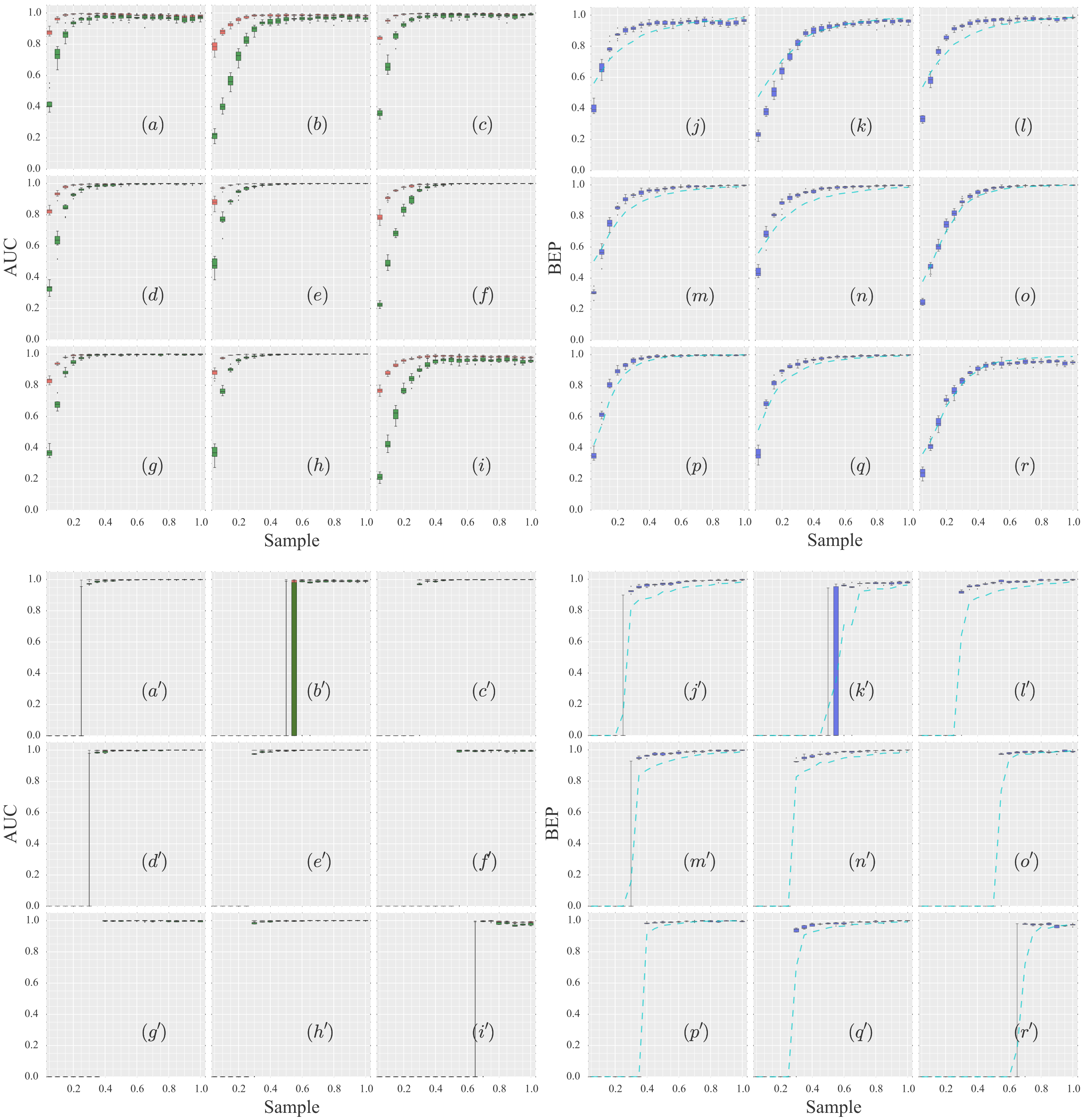

Supplementary Figure 10: Box plot of AUC and BEP of reconstructing Pseudofractal network. (a)-(i) AUROC (red box plots) and AUPR (green box plots), and (j)-(r) the real (blue box plots) and estimated BEP (dashed lines) of topology inference results in the case of explicit WTDs; (a')-(r') the same measurement indices for iteratively estimated topologies without *a priori* knowledge of associated WTDs. The benchmark WTDs are listed in Supplementary Table 3. Other parameters: network size $N = 123$, average degree $\langle k \rangle = 4.0$, hyperparameter $\beta = 0$, kernel bandwidth $h = 0.05$, WTD support $l_\tau = 8$, and error threshold $\epsilon = 10^{-4}$. Each box plot is obtained by using 10 independent realizations.

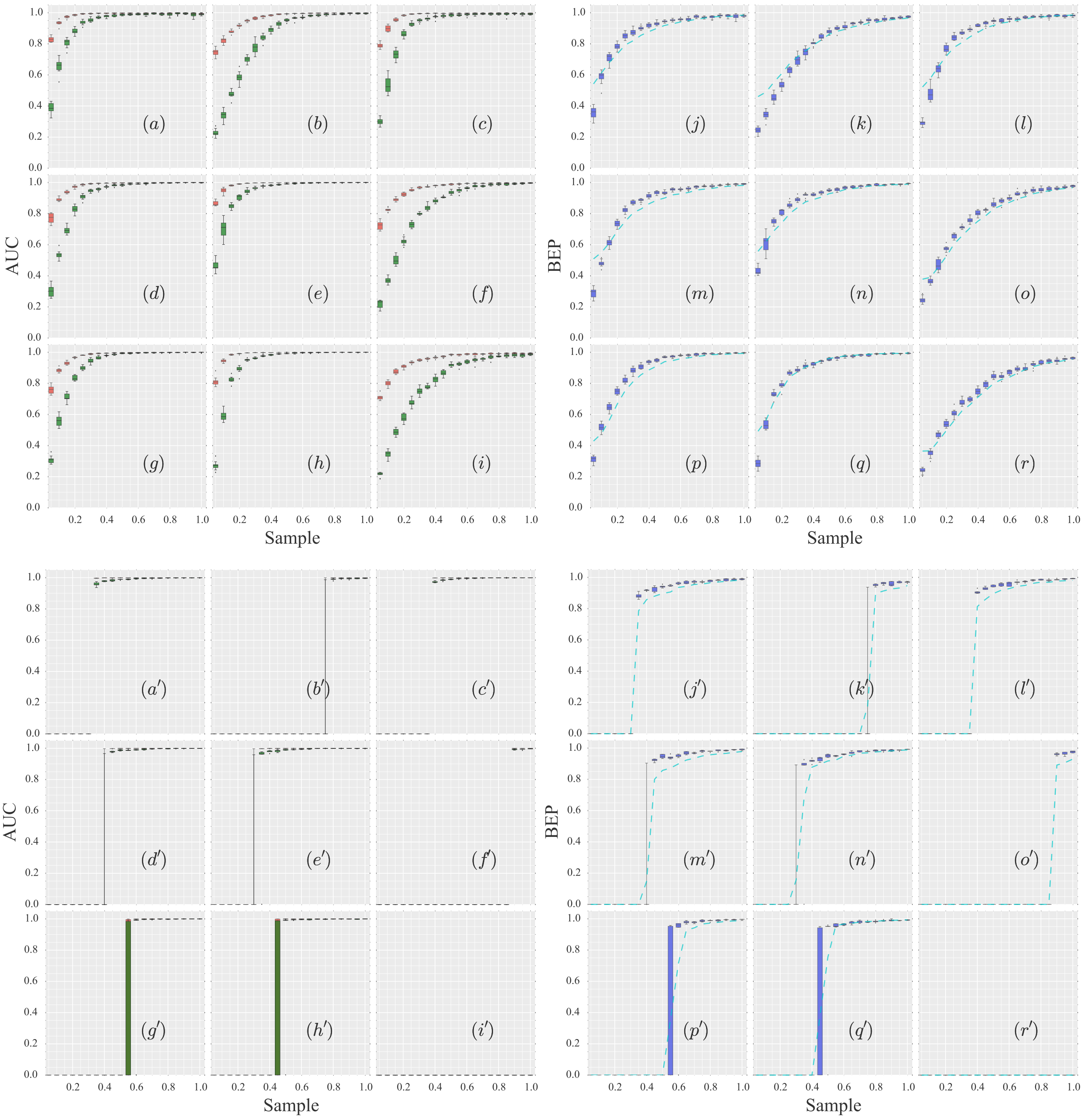

Supplementary Figure 11: Box plot of AUC and BEP of reconstructing Apollonian network. (a)-(i) AUROC (red box plots) and AUPR (green box plots), and (j)-(r) the real (blue box plots) and estimated BEP (dashed lines) of topology inference results in the case of explicit WTDs; (a')-(r') the same measurement indices for iteratively estimated topologies without *a priori* knowledge of associated WTDs. Here, the minimal sample size for simultaneously reconstructing both underlying Apollonian network and Exponential WTD is 1.27, which is out of the range of (i') and (r').The benchmark WTDs are listed in Supplementary Table 3. Other parameters: network size $N = 124$, average degree $\langle k \rangle = 5.9$, hyperparameter $\beta = 0$, kernel bandwidth $h = 0.05$, WTD support $l_\tau = 8$, and error threshold $\epsilon = 10^{-4}$. Each box plot is obtained by using 10 independent realizations.

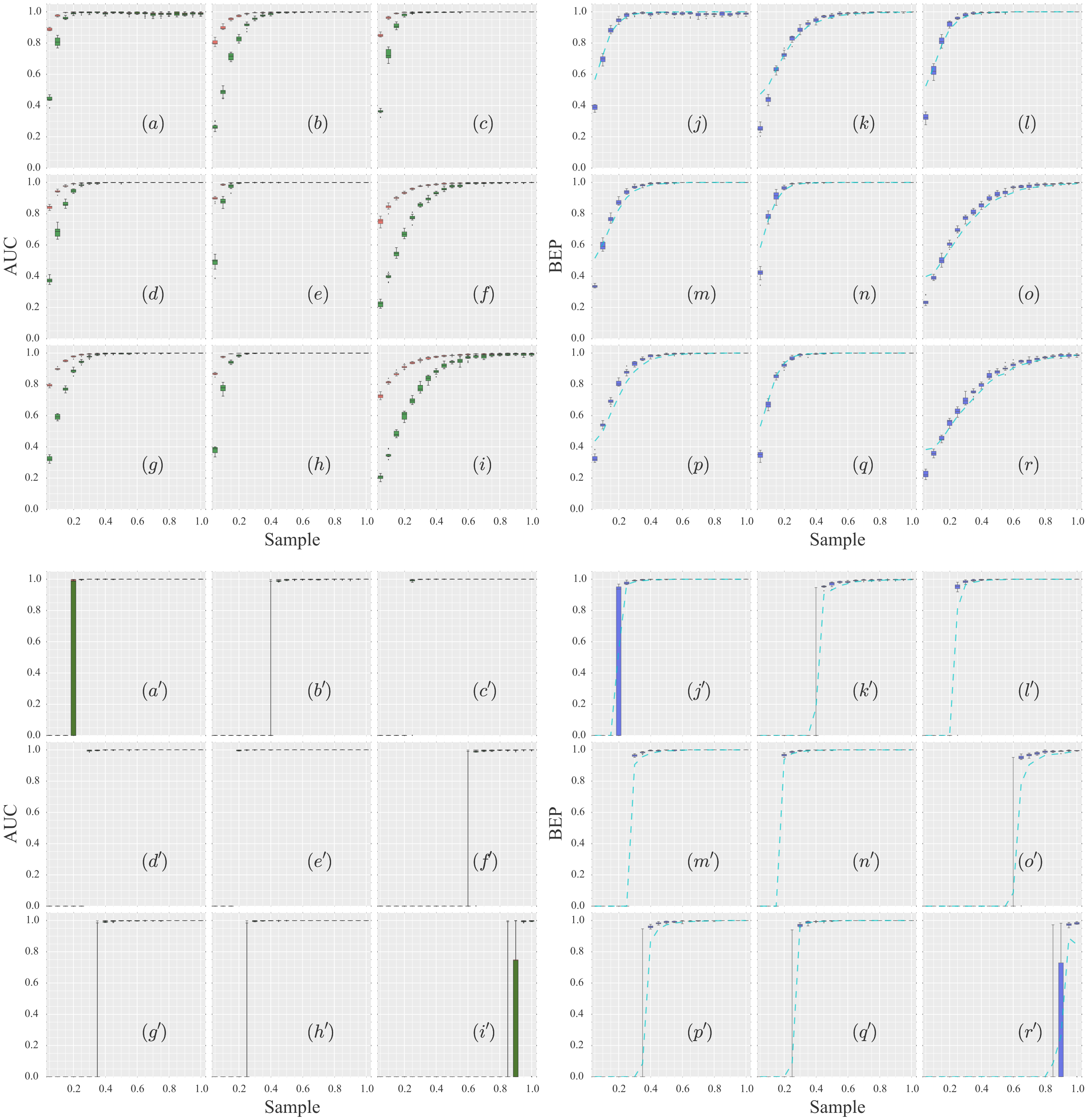

Supplementary Figure 12: Box plot of AUC and BEP of reconstructing ER random network. (a)-(i) AUROC (red box plots) and AUPR (green box plots), and (j)-(r) the real (blue box plots) and estimated BEP (dashed lines) of topology inference results in the case of explicit WTDs; (a')-(r') the same measurement indices for iteratively estimated topologies without *a priori* knowledge of associated WTDs. The benchmarkWTDs are listed in Supplementary Table 3. Other parameters: network size $N = 100$, average degree $\langle k \rangle = 5.8$, hyperparameter $\beta = 0$, kernel bandwidth $h = 0.05$, WTD support $l_\tau = 8$, and error threshold $\epsilon = 10^{-4}$. Each box plot is obtained by using 10 independent realizations.

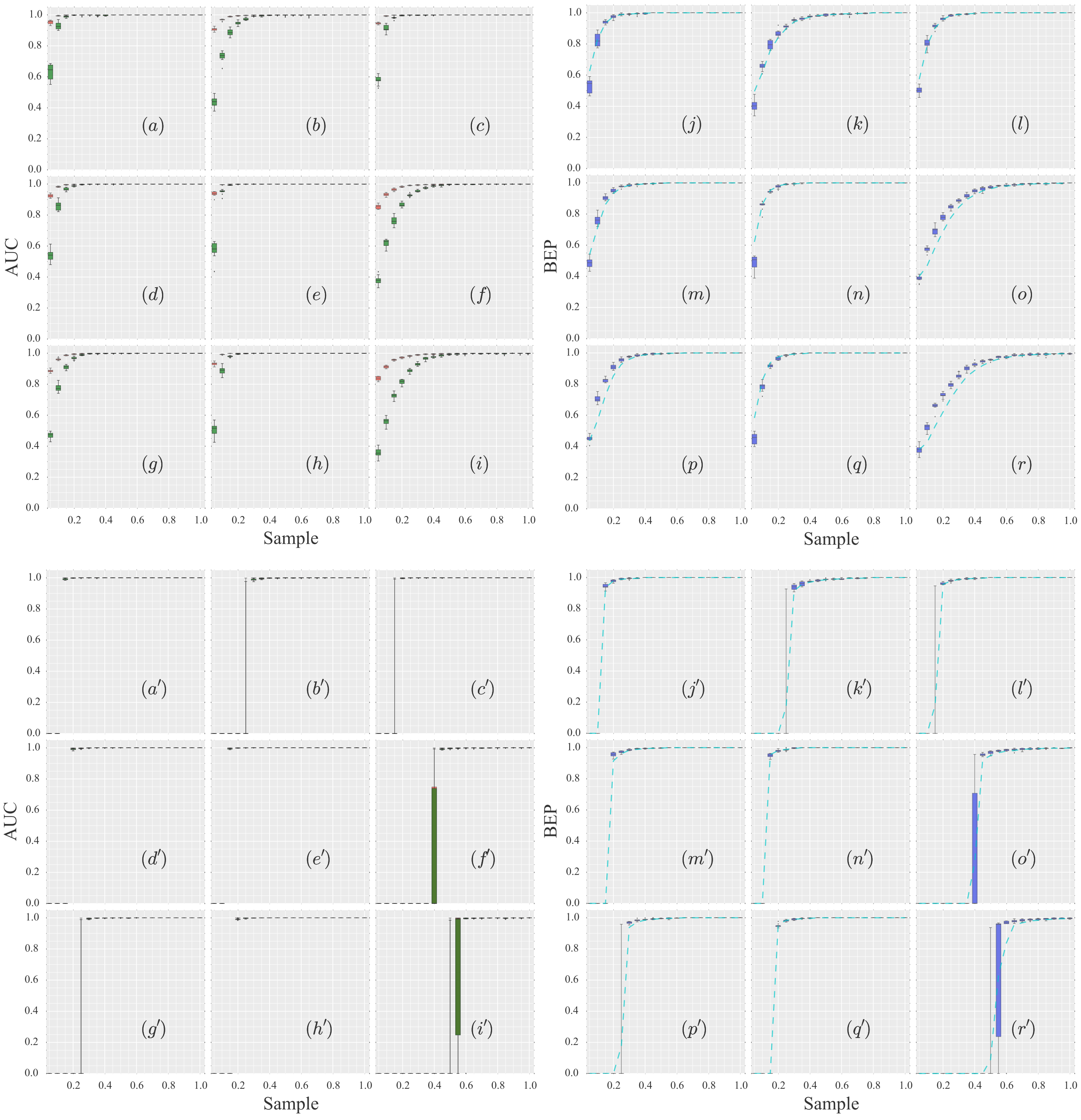

Supplementary Figure 13: Box plot of AUC and BEP of reconstructing small-world network. (a)-(i) AUROC (red box plots) and AUPR (green box plots), and (j)-(r) the real (blue box plots) and estimated BEP (dashed lines) of topology inference results in the case of explicit WTDs; (a')-(r') the same measurement indices for iteratively estimated topologies without *a priori* knowledge of associated WTDs. The benchmark WTDs are listed in Supplementary Table 3. Other parameters: network size $N = 100$, average degree $\langle k \rangle = 6.0$, hyperparameter $\beta = 0$, rewiring probability $r = 0.2$, kernel bandwidth $h = 0.05$, WTD support $l_\tau = 8$, and error threshold $\epsilon = 10^{-4}$. Each box plot is obtained by using 10 independent realizations.

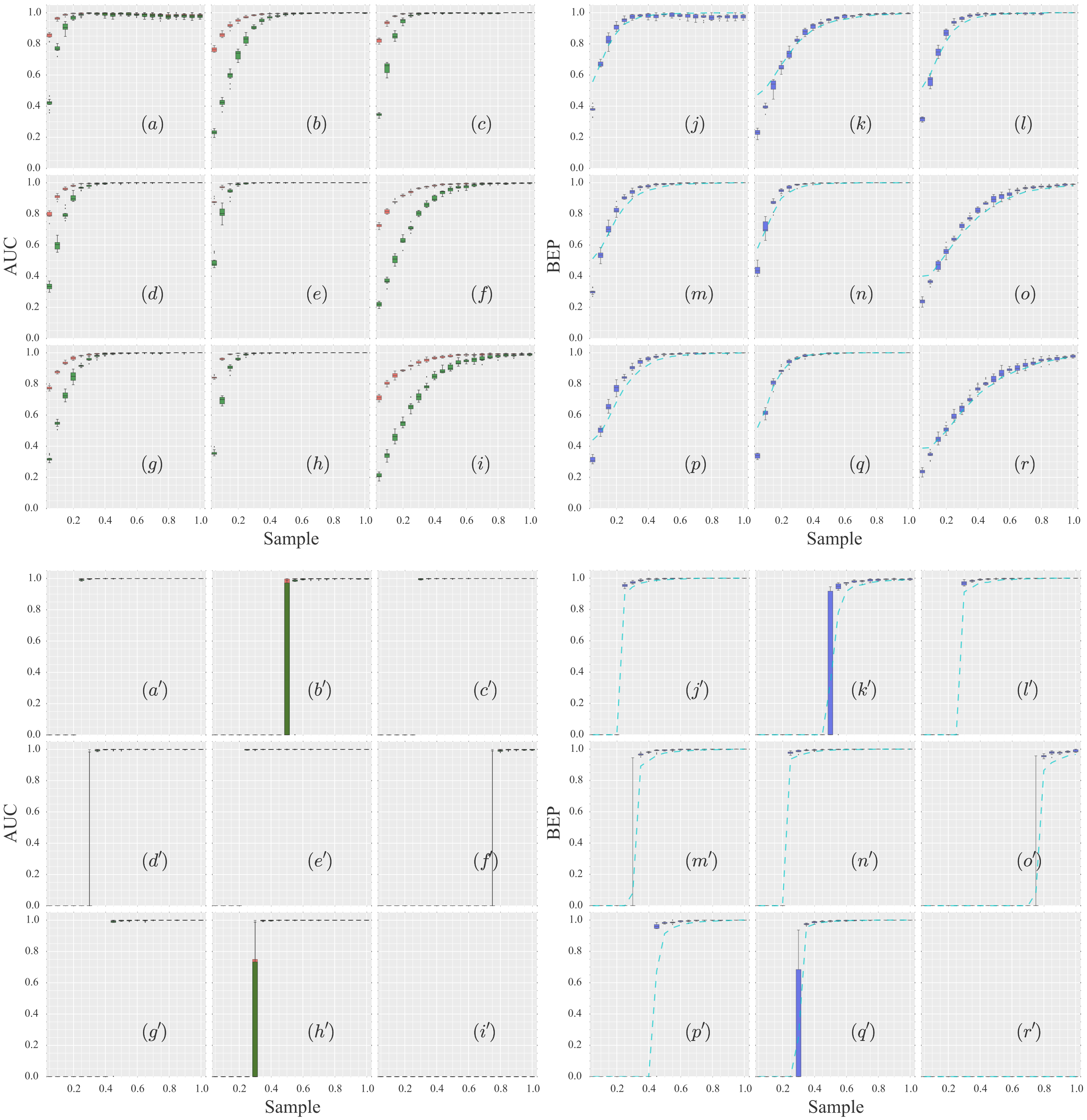

Supplementary Figure 14: Box plot of AUC and BEP of reconstructing scale-free network. (a)-(i) AUROC (red box plots) and AUPR (green box plots), and (j)-(r) the real (blue box plots) and estimated BEP (dashed lines) of topology inference results in the case of explicit WTDs; (a')-(r') the same measurement indices for iteratively estimated topologies without *a priori* knowledge of associated WTDs. Here, the minimal sample size for simultaneously reconstructing both underlying SF network and Exponential WTD is 1.02, which is out of the range of (i') and (r').The benchmark WTDs are listed in Supplementary Table 3. Other parameters: network size $N = 100$, average degree $\langle k \rangle = 5.8$, hyperparameter $\beta = 0$, kernel bandwidth $h = 0.05$, WTD support $l_\tau = 8$, and error threshold $\epsilon = 10^{-4}$. Each box plot is obtained by using 10 independent realizations.

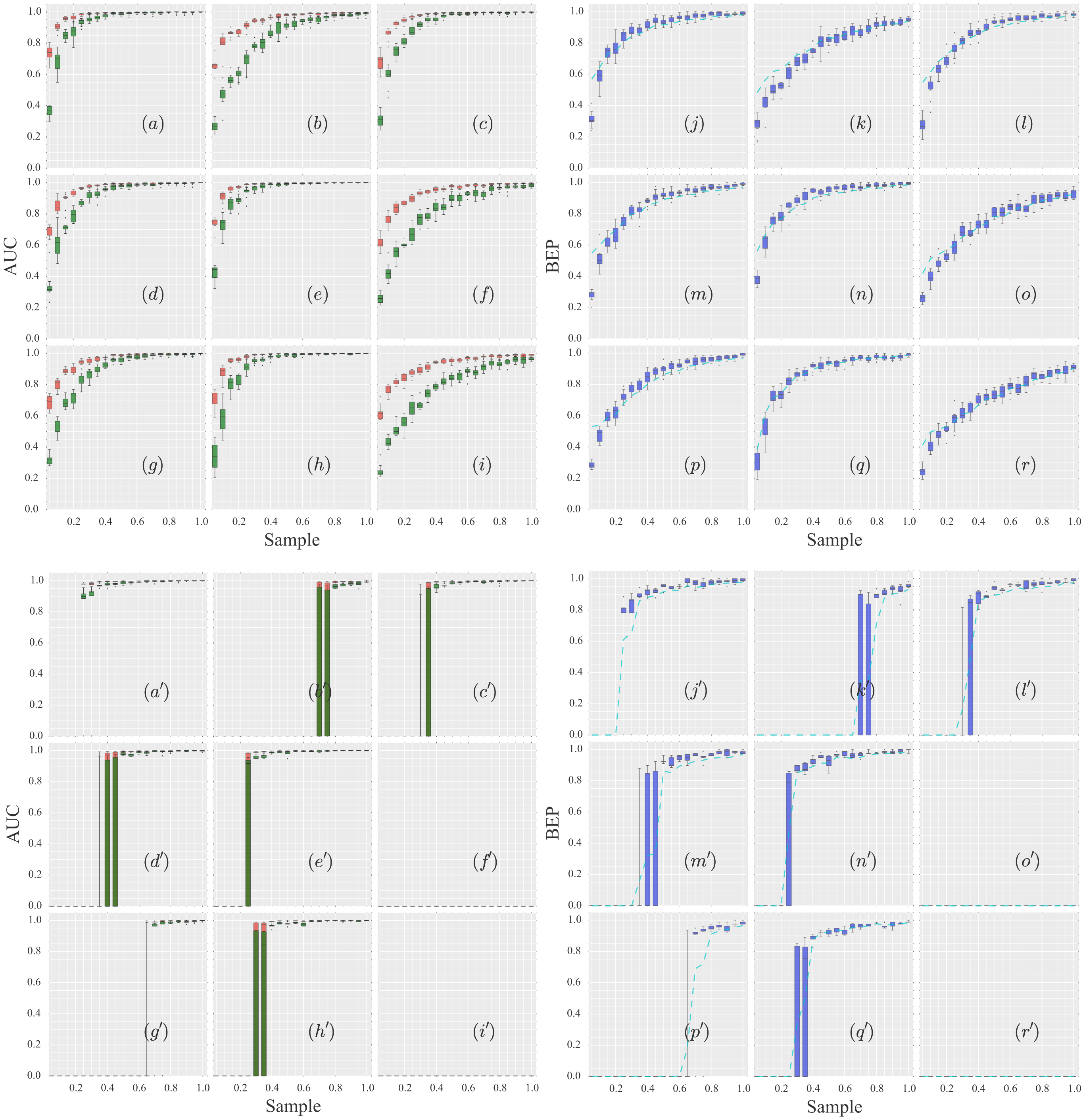

Supplementary Figure 15: Box plot of AUC and BEP of reconstructing Karate network. (a)-(i) AUROC (red box plots) and AUPR (green box plots), and (j)-(r) the real (blue box plots) and estimated BEP (dashed lines) of topology inference results in the case of explicit WTDs; (a')-(r') the same measurement indices for iteratively estimated topologies without *a priori* knowledge of associated WTDs. Here, the minimal sample size for simultaneously reconstructing both underlying Karate network and Bimodal (Exponential) WTD is 1.04 (1.38), which is out of the range of (f') and (o') [(i') and (r')]. The benchmark WTDs are listed in Supplementary Table 3. Other parameters: network size $N = 34$, average degree $\langle k \rangle = 4.6$, hyperparameter $\beta = 0$, kernel bandwidth $h = 0.05$, WTD support $l_\tau = 8$, and error threshold $\epsilon = 10^{-4}$. Each box plot is obtained by using 10 independent realizations.

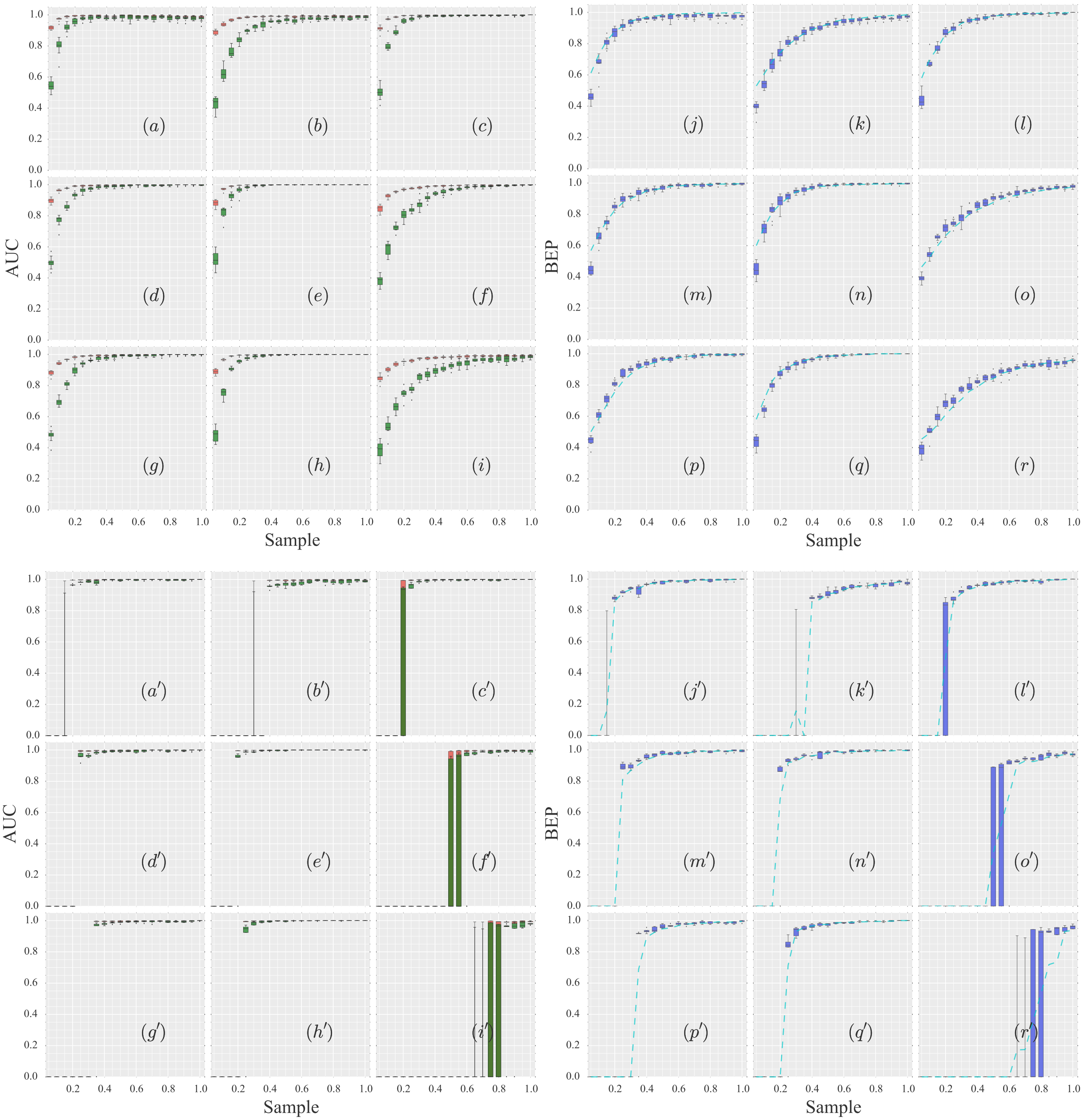

Supplementary Figure 16: Box plot of AUC and BEP of reconstructing Dolphins network. (a)-(i) AUROC (red box plots) and AUPR (green box plots), and (j)-(r) the real (blue box plots) and estimated BEP (dashed lines) of topology inference results in the case of explicit WTDs; (a')-(r') the same measurement indices for iteratively estimated topologies without *a priori* knowledge of associated WTDs. The benchmark WTDs are listed in Supplementary Table 3. Other parameters: network size $N = 62$, average degree $\langle k \rangle = 5.1$, hyperparameter $\beta = 0$, kernel bandwidth $h = 0.05$, WTD support $l_\tau = 8$, and error threshold $\epsilon = 10^{-4}$. Each box plot is obtained by using 10 independent realizations.

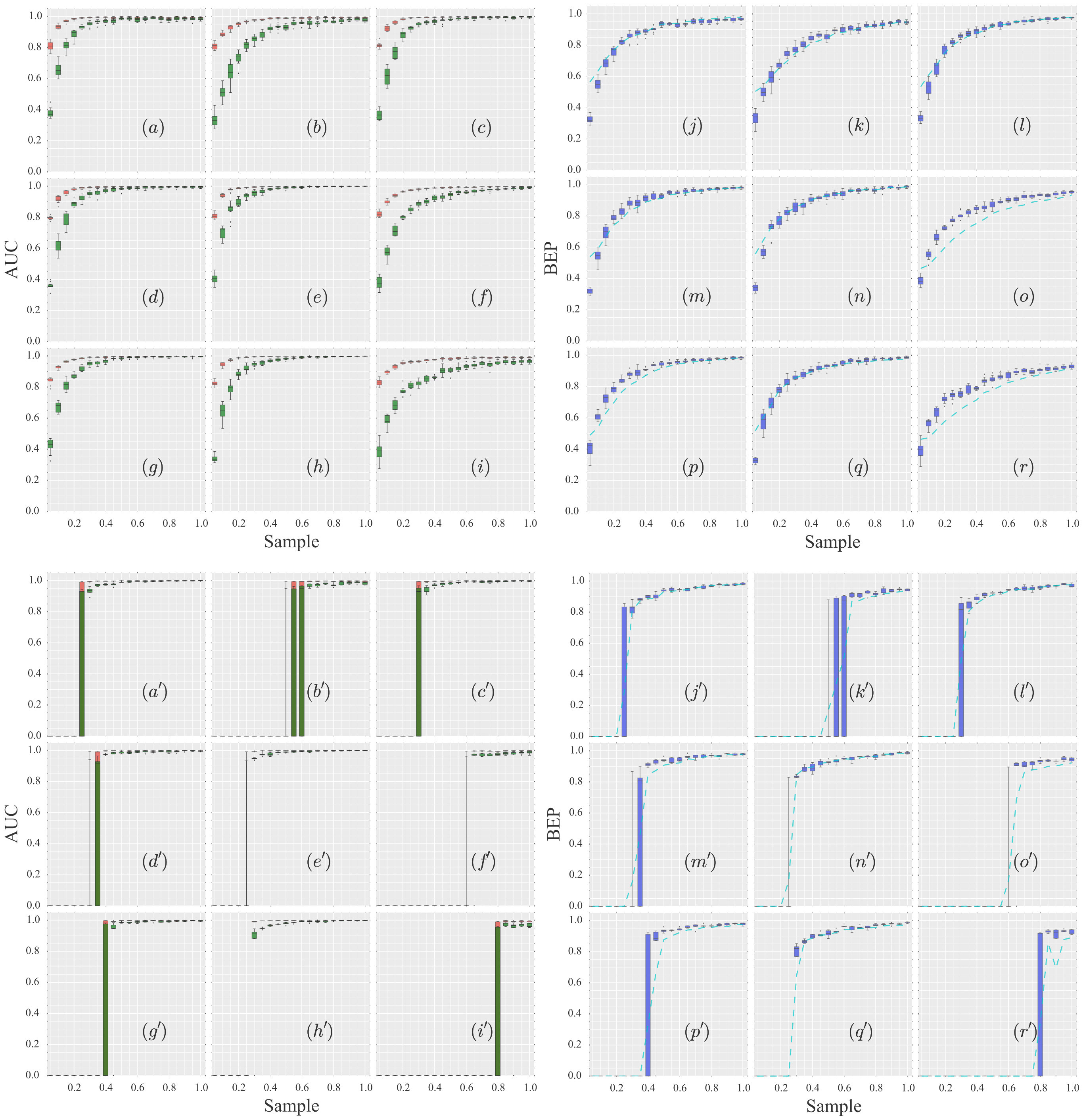

Supplementary Figure 17: Box plot of AUC and BEP of reconstructing Miserables network. (a)-(i) AUROC (red box plots) and AUPR (green box plots), and (j)-(r) the real (blue box plots) and estimated BEP (dashed lines) of topology inference results in the case of explicit WTDs; (a')-(r') the same measurement indices for iteratively estimated topologies without *a priori* knowledge of associated WTDs. The benchmark WTDs are listed in Supplementary Table 3. Other parameters: network size $N = 77$, average degree $\langle k \rangle = 6.6$, hyperparameter $\beta = 0$, kernel bandwidth $h = 0.05$, WTD support $l_\tau = 8$, and error threshold $\epsilon = 10^{-4}$. Each box plot is obtained by using 10 independent realizations.

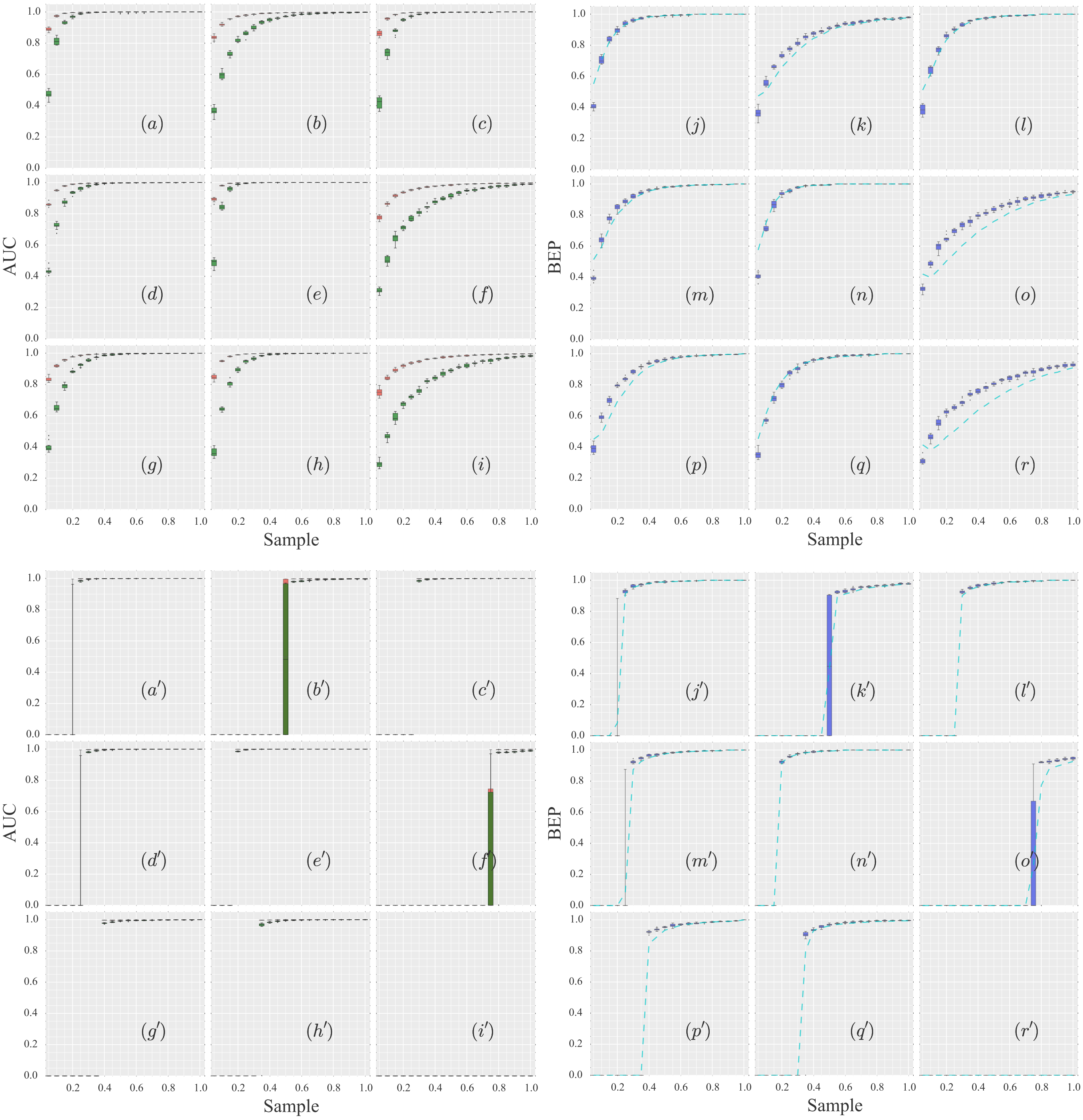

Supplementary Figure 18: Box plot of AUC and BEP of reconstructing Football network. (a)-(i) AUROC (red box plots) and AUPR (green box plots), and (j)-(r) the real (blue box plots) and estimated BEP (dashed lines) of topology inference results in the case of explicit WTDs; (a')-(r') the same measurement indices for iteratively estimated topologies without *a priori* knowledge of associated WTDs. Here, the minimal sample size for simultaneously reconstructing both underlying Football network and Exponential WTD is 1.33, which is out of the range of (i') and (r'). The benchmark WTDs are listed in Supplementary Table 3. Other parameters: network size $N = 115$, average degree $\langle k \rangle = 10.7$, hyperparameter $\beta = 0$, kernel bandwidth $h = 0.05$, WTD support $l_\tau = 8$, and error threshold $\epsilon = 10^{-4}$. Each box plot is obtained by using 10 independent realizations.

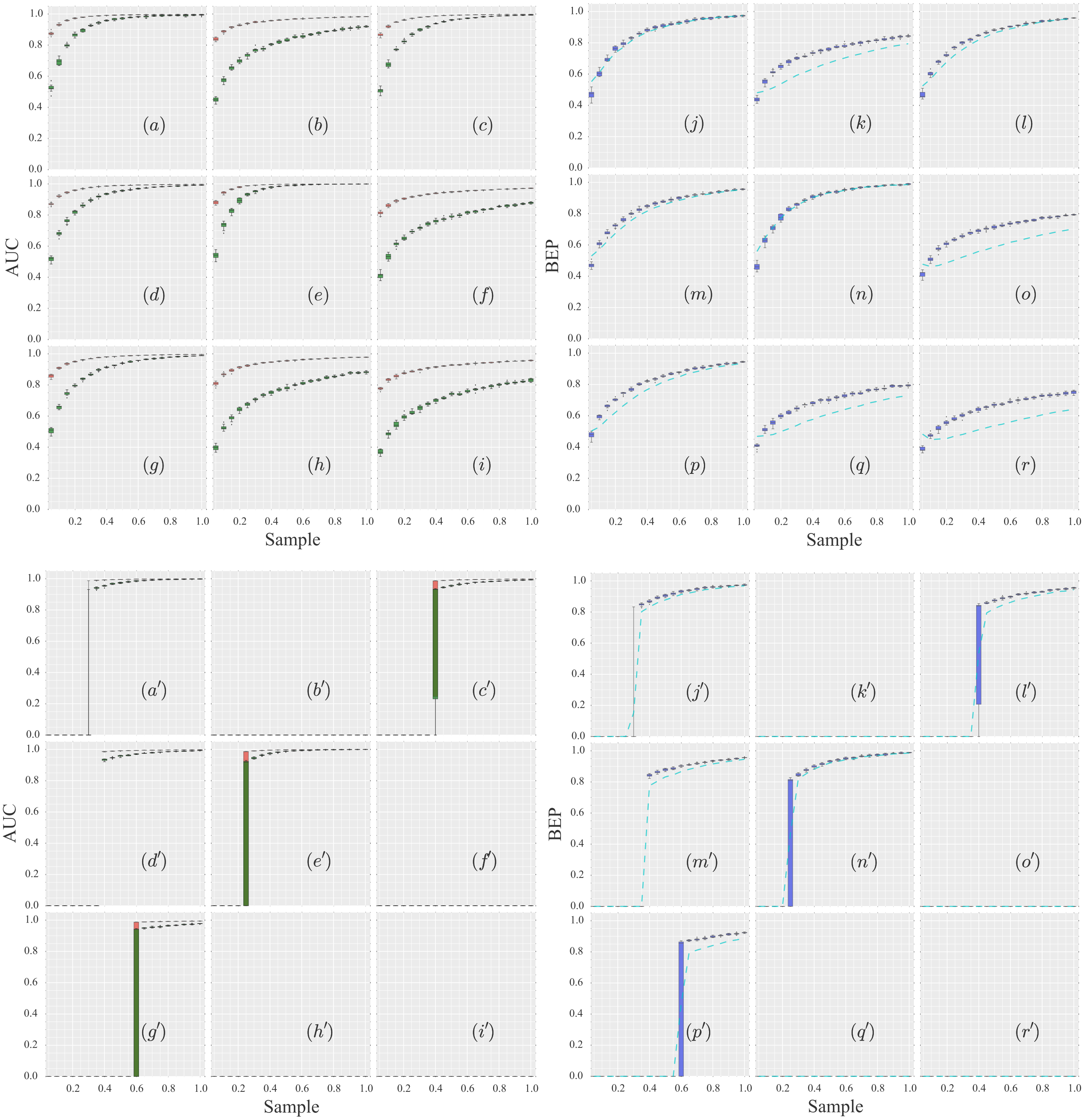

Supplementary Figure 19: Box plot of AUC and BEP of reconstructing Jazz network. (a)-(i) AUROC (red box plots) and AUPR (green box plots), and (j)-(r) the real (blue box plots) and estimated BEP (dashed lines) of topology inference results in the case of explicit WTDs; (a')-(r') the same measurement indices for iteratively estimated topologies without *a priori* knowledge of associated WTDs. Here, the minimal sample size for simultaneously reconstructing both underlying Jazz network and Weibull (Bimodal, J-shape, Exponential) WTD is 1.09 (1.66, 1.48, $-$), which is out of the range of (b') and (k') [(f') and (o'), (h') and (q'), (i') and (r')]. The dash symbol indicates a failure in recovering the underlying WTD with sample size $C/N \leq 3$ in our numerical experiments. The benchmark WTDs are listed in Supplementary Table 3. Other parameters: network size $N = 115$, average degree $\langle k \rangle = 10.7$, hyperparameter $\beta = 0$, kernel bandwidth $h = 0.05$, WTD support $l_\tau = 8$, and error threshold $\epsilon = 10^{-4}$. Each box plot is obtained by using 10 independent realizations.

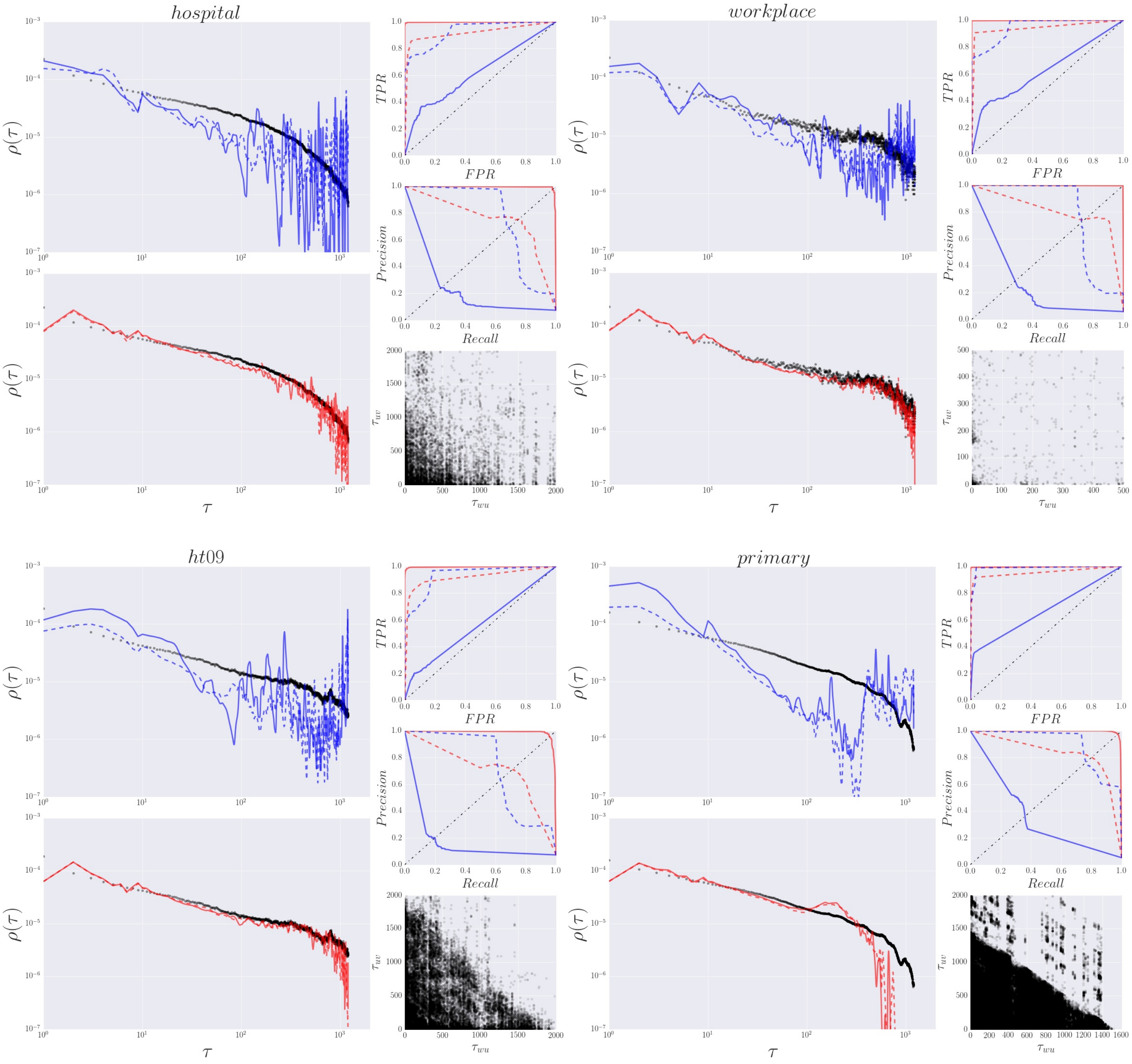

Supplementary Figure 20: Empirical validation results using realistic temporal networks. Here we applied our method to the empirical data sets, $Hospital$, $Workplace$, $HT09$ and $Primary$, respectively. (Left top) The estimated curves of WTDs from realistic diffusion data without (blue solid line) and with (blue dashed line) knowledge of the real time-aggregated graph $\mathcal{G}$, respectively. Here, the black circles correspond to mean-field distribution ρ of the empirical WTDs obtained from the raw temporal network data [equation (40)]. (Left bottom) the same estimated curves (red lines) from synthesised diffusion cascades using ρ in accordance with empirical waiting times of the corresponding dataset. (Right top and middle) The ROC and PR curves in cases of reconstruction from (1) realistic cascades without knowing ρ (blue solid lines), (2) synthesised cascades without knowing ρ (red solid lines), (3) realistic cascades given ρ (blue dashed lines), (4) synthesised cascades given ρ (red dashed lines). (Right bottom) The second-order empirical WTD $\rho(\tau_{wu}, \tau_{uv})$ of waiting times on any successive links (w, u) and (u, v) used for calculation of PCC [equation (41)] and NMI [equation (42)].

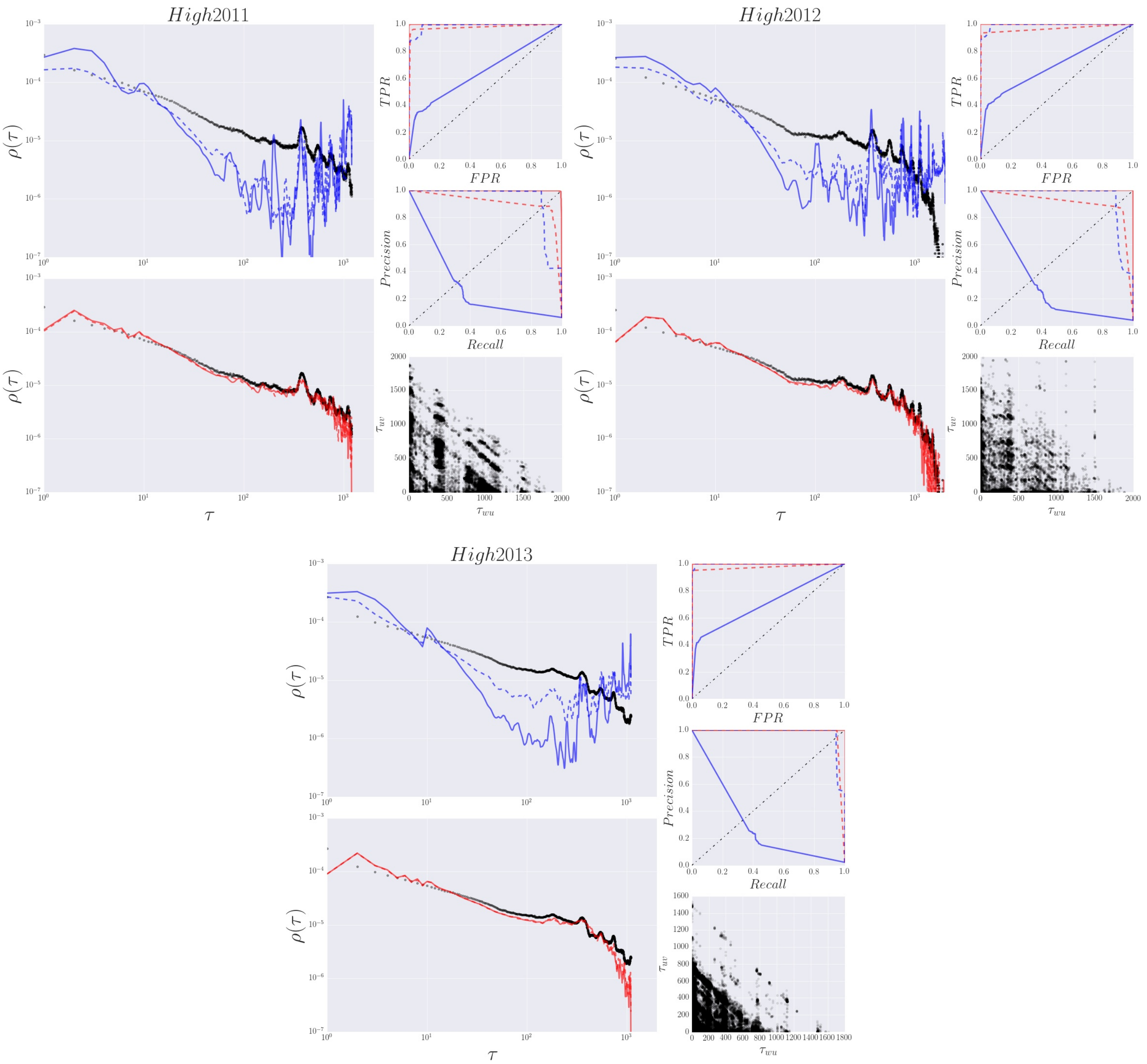

Supplementary Figure 21: Empirical validation results using realistic temporal networks. Here we applied our method to the empirical data sets, $High2011$, $High2012$, and $High2013$, respectively. (Left top) The estimated curves of WTDs from realistic diffusion data without (blue solid line) and with (blue dashed line) knowledge of the real time-aggregated graph $\mathcal{G}$, respectively. Here, the black circles correspond to mean-field distribution ρ of the empirical WTDs obtained from the raw temporal network data [equation (40)]. (Left bottom) the same estimated curves (red lines) from synthesised diffusion cascades using ρ in accordance with empirical waiting times of the corresponding dataset. (Right top and middle) The ROC and PR curves in cases of reconstruction from (1) realistic cascades without knowing ρ (blue solid lines), (2) synthesised cascades without knowing ρ (red solid lines), (3) realistic cascades given ρ (blue dashed lines), (4) synthesised cascades given ρ (red dashed lines). (Right bottom) The second-order empirical WTD $\rho(\tau_{wu}, \tau_{uv})$ of waiting times on any successive links (w, u) and (u, v) used for calculation of PCC [equation (41)] and NMI [equation (42)].

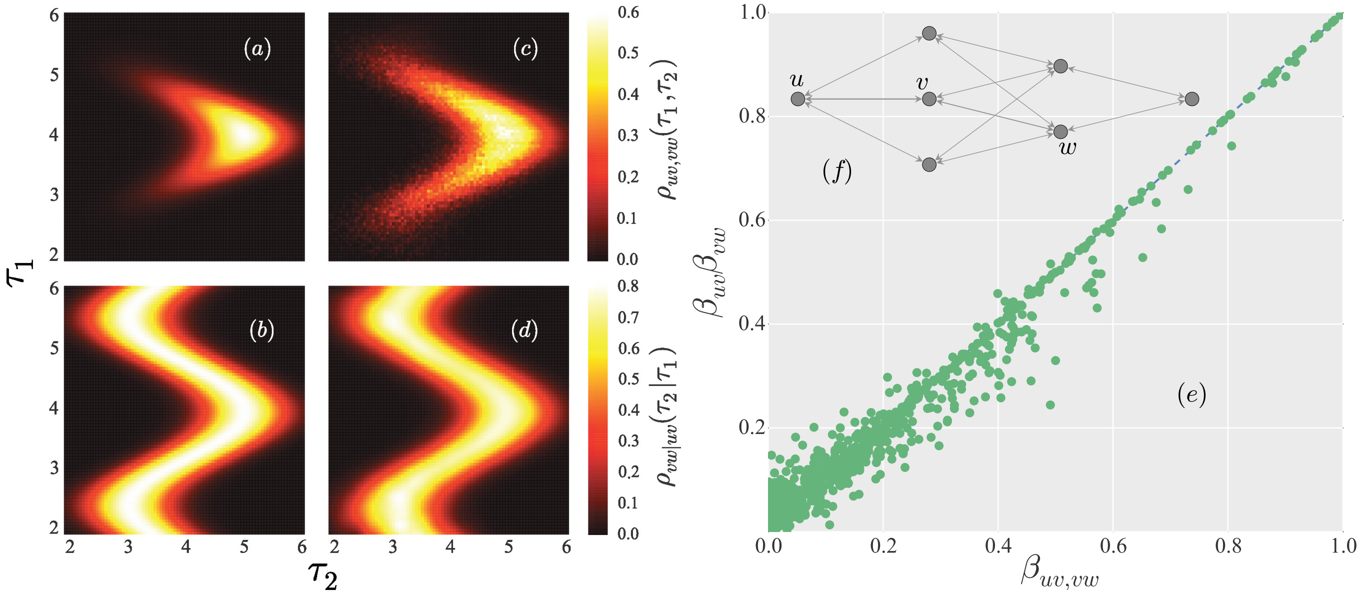

Supplementary Figure 22: Bivariate WTD estimation under the second-order STN model. (a)-(b) Colour-coded plots of real joint and conditional WTDs, $\rho_{uv,vw}(\tau_1,\tau_2) = \boldsymbol{N}\big(\tau_1; 4, 0.5^2\big)\boldsymbol{N}\big(\tau_2; \sin(2\tau_1)+4, 0.5^2\big)$, $\rho_{vw|uv}(\tau_2|\tau_1) = \boldsymbol{N}\big(\tau_2; \sin(2\tau_1)+4, 0.5^2\big)$, $\boldsymbol{N}\big(x; \mu, \sigma^2\big)$ being the PDF of Gaussian RV with mean μ and variance σ^2. (c)-(d) Estimated joint and conditional WTDs using $C = 10^4$ diffusion cascades on an illustrative network [shown in inset (f)]. Here, subplot (c) shows the original joint distribution $\hat{\rho}_{uv,vw}(\tau_1,\tau_2)$ before smoothing, which is obtained from equation (46) by replacing conditional branching coefficients [equation (47)] by the joint probabilities [equation (44)]. Initially, we set $\hat{\rho}^0_{uv,vw} = (\tau_1,\tau_2) = \boldsymbol{N}\big(\tau_1; 4, 0.5^2\big)\boldsymbol{N}\big(\tau_2; 4, 0.5^2\big)$. The self-consistently estimated conditional WTD $\hat{\rho}_{vw|uv}(\tau_2|\tau_1)$ agrees well with the real distribution, as displayed in (b) and (d). Other parameters: kernel bandwidth $h = 0.05$, and error threshold $\epsilon = 10^{-4}$. (e) Product of branching coefficients for two successive links $\beta_{uv}\beta_{vw}$ versus joint branching coefficients $\beta_{uv,vw}$ for 10^3 independent cascades initialised by source u. The deviation of the curve from the negative diagonal of the unit square (dashed line) reveals correlated properties of waiting times occurring on successive links.

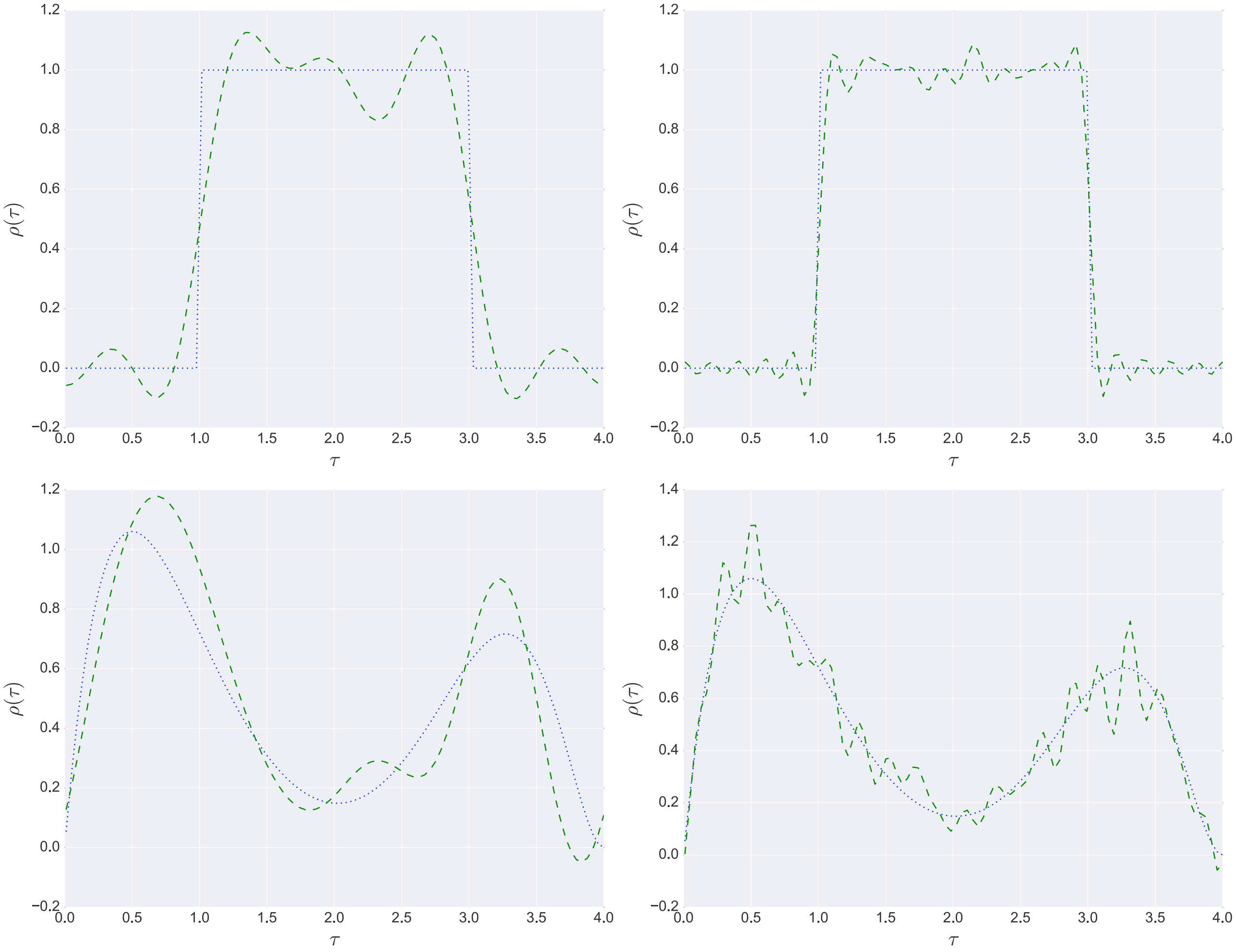

Supplementary Figure 23: Fourier series based estimation for WTDs. Here we illustrate the Fourier series method for density estimation using two benchmark tests: uniform (top panel) and bimodal (bottom panel) distributed WTDs. The time-aggregated network is the ER random network of size $N = 100$ and average degree $\langle k \rangle = 5.8$, which we assume to be explicitly given for the density estimator. Other parameters: WTD support $l_\tau = 4$, relative sample size $C/N = 0.5$, kernel smoother $K_h(\tau) = \delta(\tau)$. The order of truncated Fourier series: (left panel) $K = 5$, (right panel) $K = 20$. Note that using an improper (inadequate or exorbitant) order of series incompatible with sample size may potentially cause the under- or overfitting problem.

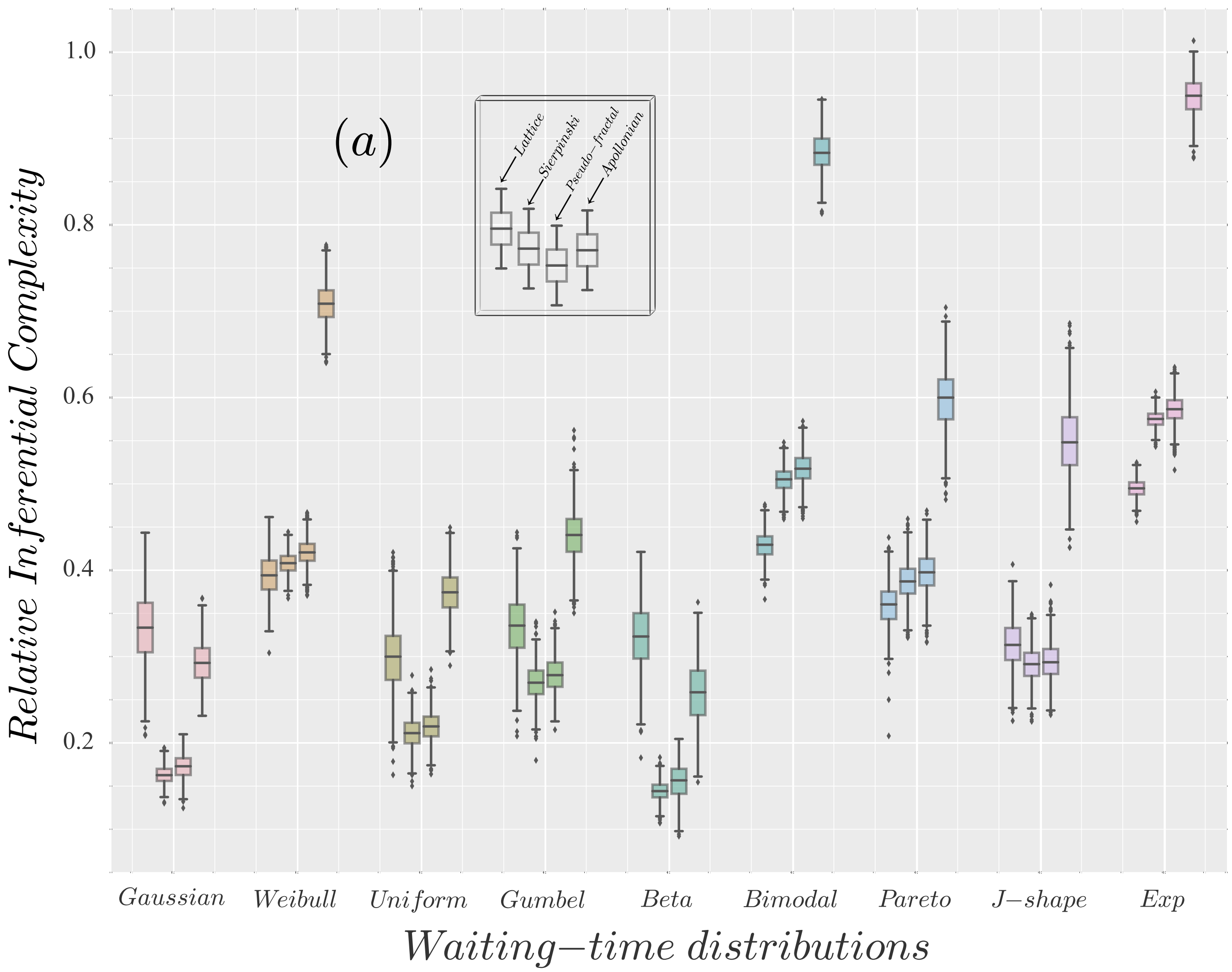

Supplementary Figure 24: Inferential complexity of benchmark STNs. Box plot of relative inferential complexity of diffusion structure for four time-aggregated graphs (Lattice, Sierpinski, Pseudo-fractal and Apollonian) in combination of with different types of WTDs. Here, relative inferential complexity [$\equiv N^{-1}\langle\xi(\mathcal{D},\mathcal{N}_{\rm s})\rangle_D$] represents the entropic complexity metric divided by the network size, where the angular brackets $\langle\cdot\rangle_D$ denote the average over ensembles of diffusion cascades D. The benchmark time-aggregated networks are listed in Supplementary Table 1, and the underlying WTDs are listed in Supplementary Table 3. Each box plot is obtained by using 1000 independent realisations.

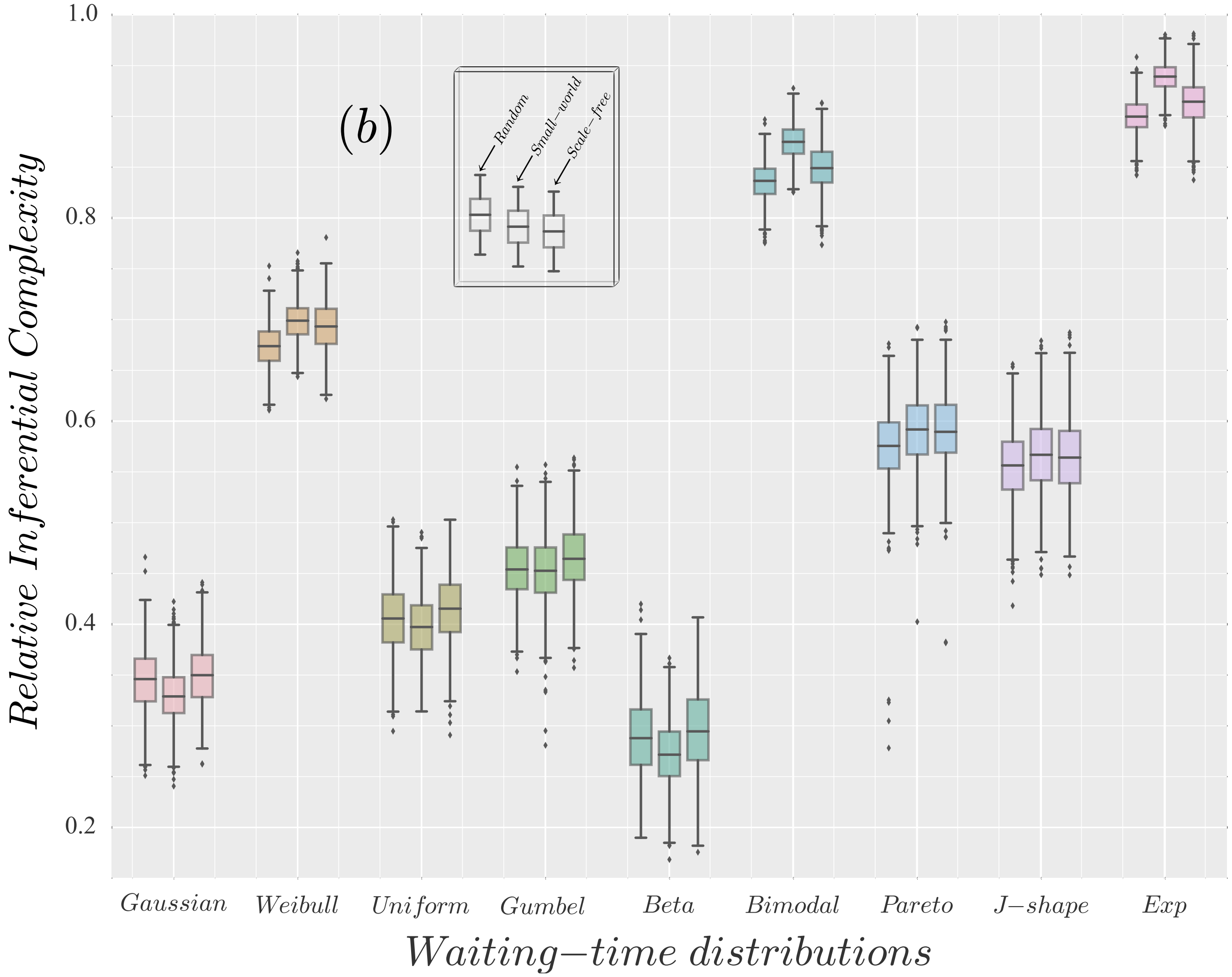

Supplementary Figure 25: Inferential complexity of benchmark STNs. Box plot of relative inferential complexity of diffusion structure for three time-aggregated graphs (Random, Small-world and Scale-free) in combination of with different types of WTDs. Here, relative inferential complexity [$\equiv N^{-1}\langle\xi(\mathcal{D}, \mathcal{N}_{\rm s})\rangle_D$] represents the entropic complexity metric divided by the network size, where the angular brackets $\langle\cdot\rangle_D$ denote the average over ensembles of diffusion cascades D. The benchmark time-aggregated networks are listed in Supplementary Table 1, and the underlying WTDs are listed in Supplementary Table 3. Each box plot is obtained by using 1000 independent realisations.

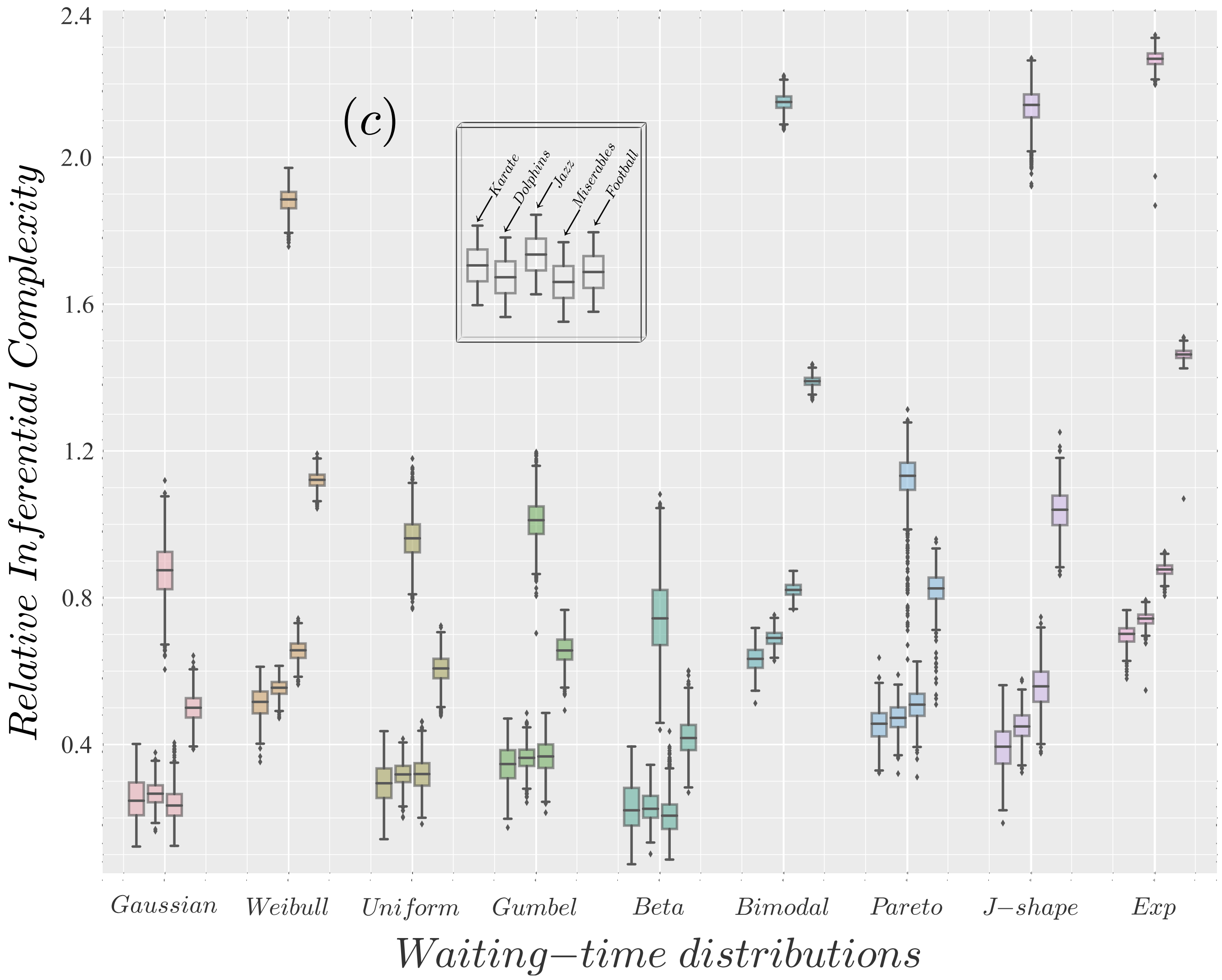

Supplementary Figure 26: Inferential complexity of benchmark STNs. Box plot of relative inferential complexity of diffusion structure for five time-aggregated graphs (Karate, Dolphin, Jazz, Miserables and Football) in combination of with different types of WTDs. Here, relative inferential complexity [$\equiv N^{-1}\langle \xi(\mathcal{D}, \mathcal{N}_{\mathrm{s}}) \rangle_D$] represents the entropic complexity metric divided by the network size, where the angular brackets $\langle \cdot \rangle_D$ denote the average over ensembles of diffusion cascades D. The benchmark time-aggregated networks are listed in Supplementary Table 1, and the underlying WTDs are listed in Supplementary Table 3. Each box plot is obtained by using 1000 independent realisations.

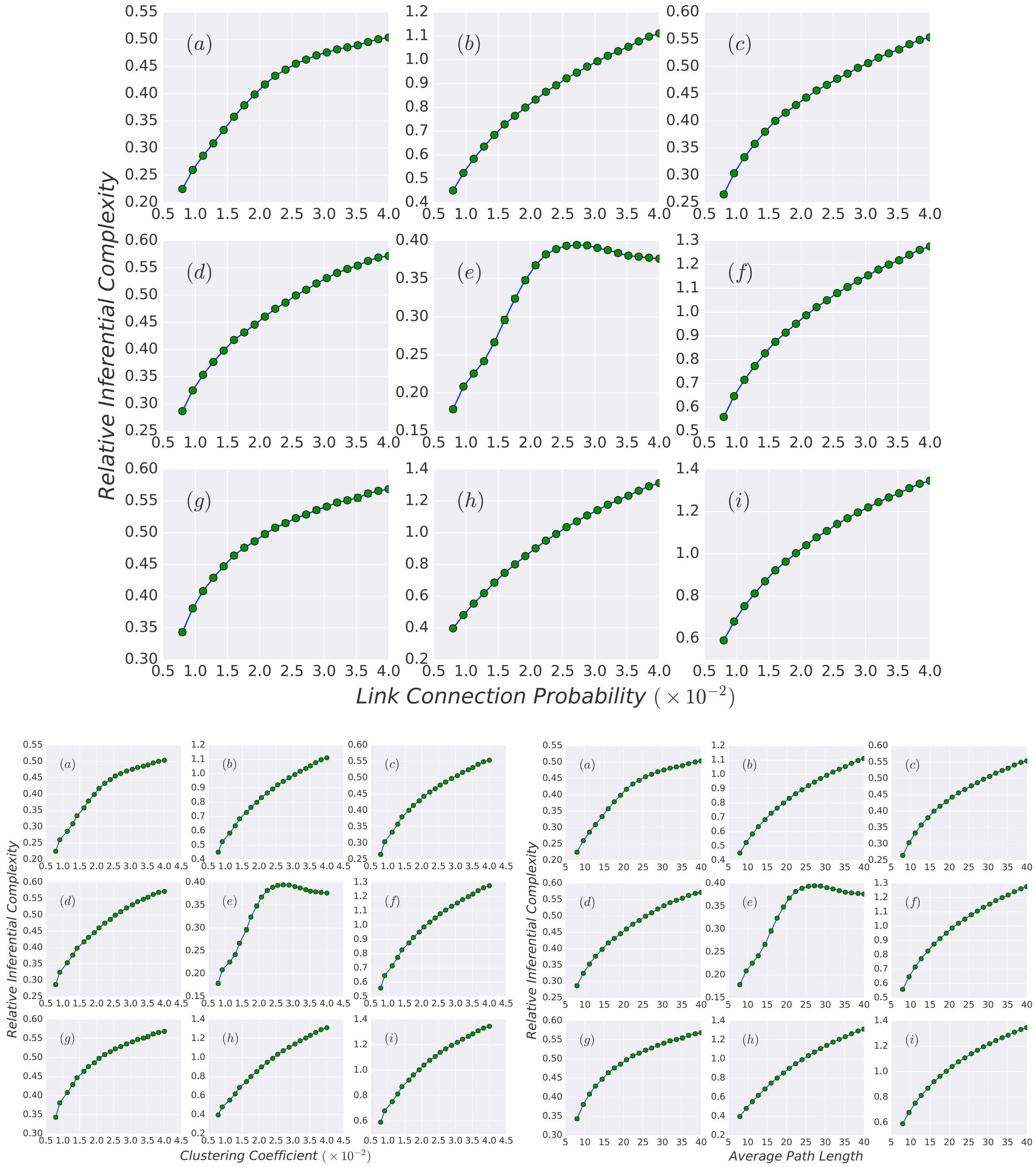

Supplementary Figure 27: Inference complexity of random graphs. Relative inference complexity as functions of link connection probability of random networks (top), as well as average clustering coefficient (left bottom) and path length (right bottom). The underlying WTDs [(a)-(i)] are listed in Supplementary Table 3. The time-aggregated networks are generated using Erdös-Rényi model. The network size is 1000. Each data point is obtained by averaging over 20 independent networks and 1000 independent diffusion cascades. Error bars denote the standard deviations.

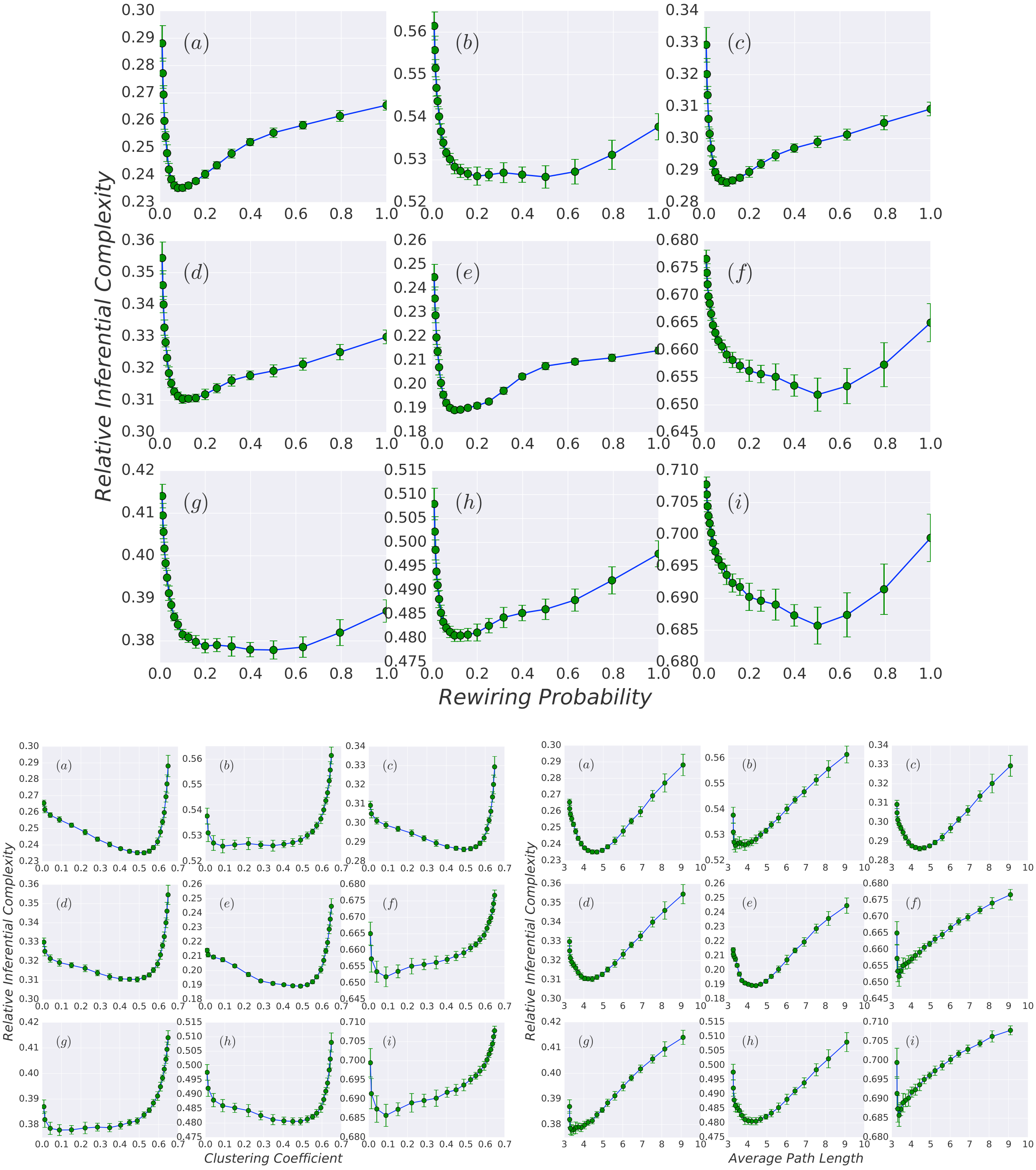

Supplementary Figure 28: Inference complexity of small-world graphs. Relative inference complexity as functions of link rewiring probability of small-world graphs (top), as well as average clustering coefficient (left bottom) and path length (right bottom). The underlying WTDs [(a)-(i)] are listed in Supplementary Table 3. The time-aggregated networks are generated using Watts-Strogatz model. The network size is 1000 and the average node degree is 20. Each data point is obtained by averaging over 20 independent networks and 1000 independent diffusion cascades. Error bars denote the standard deviations.

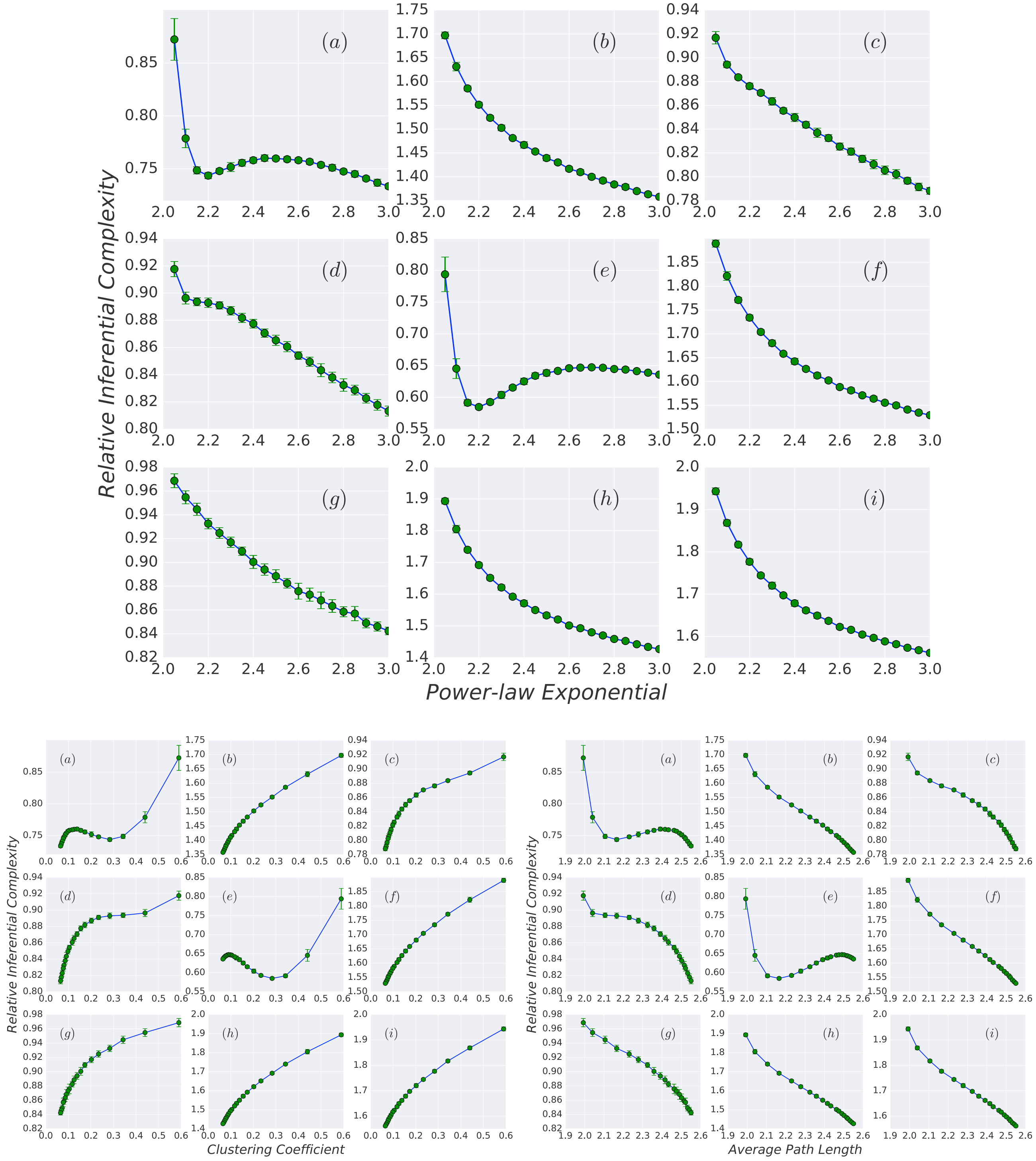

Supplementary Figure 29: Inference complexity of scale-free graphs Relative inference complexity as functions of power-law exponentials of degree distributions of scale-free graphs (top), as well as average clustering coefficient (left bottom) and path length (right bottom). The underlying WTDs [(a)-(i)] are listed in Supplementary Table 3. The time-aggregated networks are generated using Price model with tunable power-law exponentials of degree distribution, $P(k) \sim k^{-\gamma}, \gamma \in (2, 3]$. The network size is 1000 and the average node degree is 20. Each data point is obtained by averaging over 20 independent networks and 1000 independent diffusion cascades. Error bars denote the standard deviations.

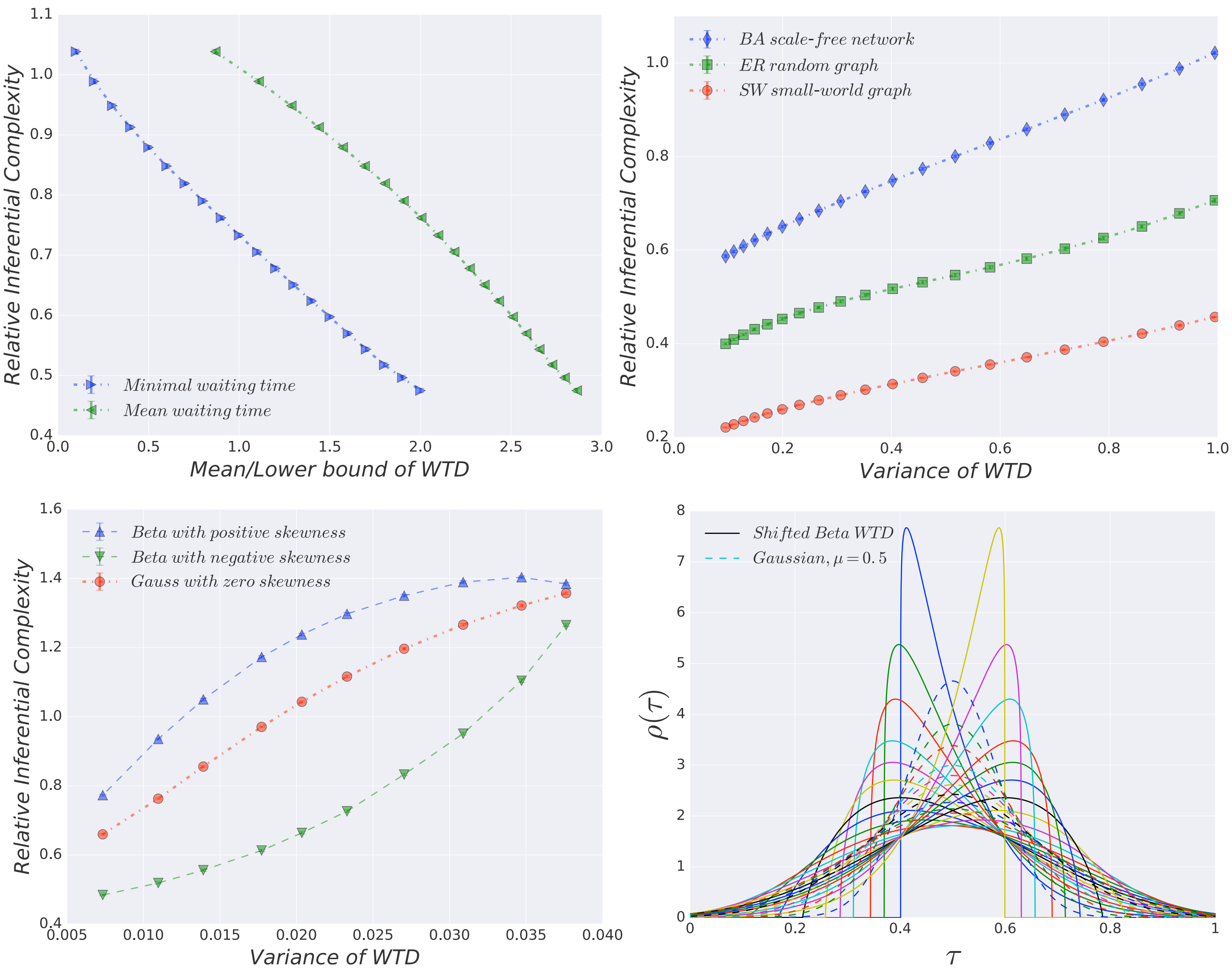

Supplementary Figure 30: Effects of distributional features of WTDs on inferential complexity. Here we considered three types of WTDs, $Pareto(\tau; a, b)$ with varying location parameters a (left top) and power-law exponentials b (right top) , shifted $Beta(\tau - c; \boldsymbol{\theta})$ and $Gauss(\tau; \mu, \sigma^2)$ sharing a common mean $\mu = 0.5$ and varying variances σ^2 (left bottom). Distributional details can be found in Supplementary Table 3. The time-aggregated graphs are Barabási-Albert scale-free networks, unless otherwise specified (right top). The network size is 1000 and the average node degree is 20. Each data point is obtained by averaging over 20 independent networks and 1000 independent diffusion cascades. Error bars denote the standard deviations. Left bottom: The curves of the Beta and Gaussian WTDs used for comparison of effects of distributional variance and skewness on the inferential complexity of STN models. In order to decouple from the influence of unmatched first two moments, we select location parameter c_i in the shifted $Beta(\tau - c_i; \boldsymbol{\theta}_i)$ to ensure that their means coincide with that of $Gauss(\tau; \mu, \sigma_i^2)$ centred at $\mu = 0.5$, and select Gaussian variances δ_i^2 in accordance with those of $Beta(\tau - c_i; \boldsymbol{\theta}_i)$. The specific values of shape parameters of Beta WTDs are set to $\boldsymbol{\theta}_i = (a_i, b_i)$ with $a_i = 1.1, 1.2, 1.3, 1.4, 1.5, 1.6, 1.8, 2.0, 2.3, 2.6, 3, 3.5, 4, 4.5, 5, 5.5, 6, 7, 8, 10$ and $b_i = a_{21-i}, i = 1, \ldots, 20$, and location parameters are $c_i \doteq 0.10, 0.13, 0.16, 0.19, 0.21, 0.24, 0.29, 0.33, 0.40, 0.46, i = 1, \ldots, 10$, $c_i = 1 - c_{21-i}, i = 11, \ldots, 20$. From our symmetric choice of shape parameters it provides a monotone decrease in distributional skewness (i.e., normalised third central moment) of Beta WTDs, and in particular, we have $\gamma_i = -\gamma_{21-i}$, where γ_i is the skewness of Beta distribution with shape parameter $\boldsymbol{\theta}_i$. Our numerical results show a positive correlation between WTD skewness and inferential complexity (left bottom), which also coincides with the previous observation that inferential complexity of STN models is negatively correlated with the lower bound of underlying WTDs.

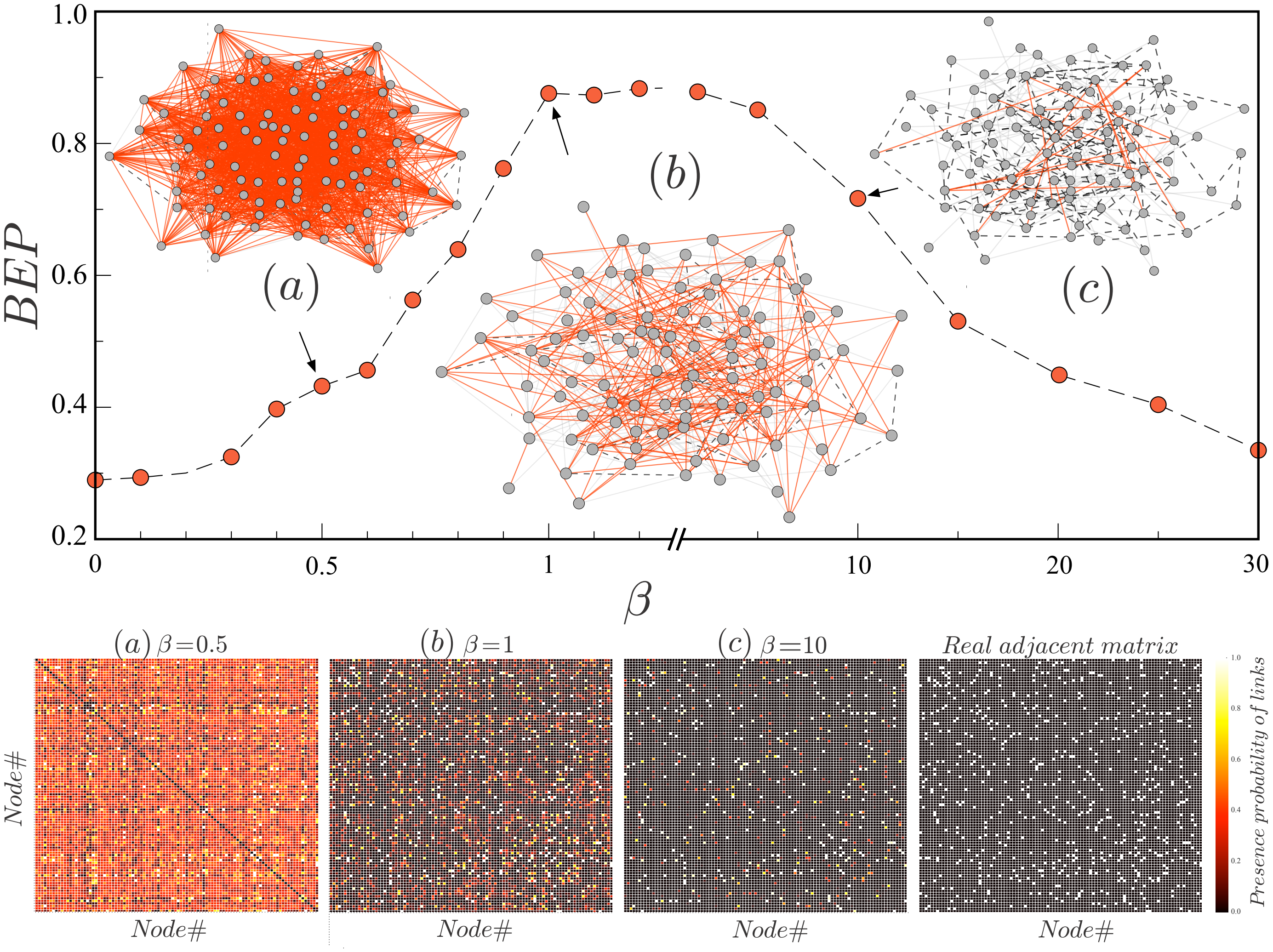

Supplementary Figure 31: Reconstruction accuracy BEP versus penalty size β of sparsity priors. Inset: single configurations of the underlying network inferred with different hyperparameters (a) $\beta = 0.5$, (b) $\beta = 1$, and (c) $\beta = 10$. Here, erroneous presence and erroneous absence of links are respectively represented by red solid and black dashed lines. The sample size used for inference tasks is $C/N = 0.7\theta_{\text{WTD}}$. The underlying time-aggregated network, the associated WTD and other parameters are the same as Supplementary Figure 6. The optimal parameter range of β is nearly 1 to 5 in the given scene. Bottom panels: (a)-(c) Heatmaps of posterior presence probabilities of link (u, v), $\hat{q}_{uv} = M^{-1} \sum_{\hat{\mathcal{G}} \in \hat{\mathbf{G}}} \mathbb{1}_{[(u,v) \in \hat{\mathcal{G}}]}$ under different hyperparameters. Here, the two extreme cases of (a) $\beta = 0.5$ and (c) $\beta = 10$ yield substantial Type I and Type II error rates, respectively. The real adjacent matrix of the underlying network is also plotted for comparison.

Supplementary Tables

Supplementary Table 1: Summary of the time-aggregated networks used in the paper. Here, N denotes the network size, L denotes the number of links, and $\langle k \rangle$ represents the average degree of a network.

Networks	N	L	$\langle k \rangle$	Description
Lattice 2D	100	180	3.6	Square lattice with non-periodic boundary conditions
Sierpinski [6]	123	243	4.0	Network derived from Sierpinski gasket variants
Pseudofractal [7]	123	243	4.0	Pseudofractal scale-free web with exponent $\gamma = 1 + \ln 3 / \ln 2$
Apollonian [8]	124	366	5.9	Network derived from Apollonian packing
Random [9]	100	291	5.8	Random graph generated by Erdös-Rényi (ER) model
Small-world [10]	100	300	6.0	Small-world Network generated by Watts-Strogatz (WS) model
Scale-free [11]	100	291	5.8	Scale-free network generated by Barabási-Albert (BA) model
Karate [12]	34	78	4.6	Network of friendship between members of a karate club
Dolphin [13]	62	159	5.1	Network of frequent associations between 62 dolphins
Miserables [14]	77	254	6.6	Coappearance network of characters in the novel *Les Miserables*
Football [15]	115	613	10.7	Network of American college football games
Jazz [16]	198	2742	27.7	Network of jazz musicians

Supplementary Table 2: Summary of the empirical data set in the paper. Here, N denotes the network size, L denotes the number of temporal links. The temporal resolution of the temporal networks is $\delta t = 20sec$.

Networks	N	L	Description
Hospital [17]	75	32424	Temporal contacts between patients and health-care workers of a hospital
Workplace [18]	92	9827	Face-to-face temporal contacts between individuals in an office building
HT09 [19]	113	20818	Face-to-face proximity network during ACM Hypertext 2009 conference
Primary [20]	92	9827	Temporal contacts between students and teachers of a primary school
High2011 [21]	126	28561	Temporal contacts between students and teachers of a high school
High2012 [21]	180	45047	
High2013 [21]	327	188508	

Supplementary Table 3: Summary of benchmark WTDs used in the paper. Here, a denotes the location parameter, b denotes the shape parameter, and $f(x;\boldsymbol{\theta})$ represents the PDF with the corresponding parameter set $\boldsymbol{\theta} = \{a, b\}$.

WTDs	a	b	$f(x;\boldsymbol{\theta})$	Description/Scope of application
Gaussian	2	0.4	$\frac{1}{\sqrt{2\pi b^2}}\exp(-(x-a)^2/2b^2)$	Central limit theorem/saddle-point approximation
Weibull	1	2.5	$\frac{b}{a^b}x^{b-1}\exp(-(x/a)^b)$	Modelling lifetime events in survival analysis [1]
Uniform	1	3	$\Theta(x-a)\Theta(b-x)$	Flat distributions [$\Theta(\cdot)$ is Heaviside step function]
Gumbel	3	2	$abx^{-a-1}\exp(-bx^{-a})$	Arrival times of epidemics in metapopulations [2]
Beta**	9	2	$\frac{\Gamma(a+b)}{\Gamma(a)\Gamma(b)}x^{a-1}(1-x)^{b-1}$	(Semi-)parametric Bayesian data analysis [3]
Bimodal	a_1=2<br>a_2=9	b_1=8<br>b_2=3	$\frac{1}{2}\mathrm{Beta}(a_1,b_1)+\frac{1}{2}\mathrm{Beta}(a_2,b_2)$	Described by linear combinations of $\mathrm{Beta}(a_i,b_i)$
Pareto	0.3	0.5	$\frac{b}{a}(x/a)^{-b-1}\Theta(x-a)$	Typical interevent-times of human activities [4]
J-shape	a=0.3<br>c=4	0.5	$\frac{a^{-b}}{a^{-b}-c^{-b}}\mathrm{Pareto}(c-x;\boldsymbol{\theta})\Theta(x)$	Reflected $\mathrm{Pareto}(x;\boldsymbol{\theta})$ [with respect to $x=c$]
Exp	–	1	$\frac{1}{b}\exp(-x/b)$	Memoryless contact process (hazard $\lambda \equiv \frac{1}{b}$) [5]

**In numerical experiments, we used the rescaled Beta distribution, $\mathrm{Beta}_{l_\tau}(x;\boldsymbol{\theta}) = l_\tau^{-1}\mathrm{Beta}(x/l_\tau;\boldsymbol{\theta})$, with support on $[0, l_\tau]$. Here we set the rescaling parameter as $l_\tau = 4$ for comparison with the other benchmark WTDs (also see Supplementary Figure 3).

Supplementary Table 4: Summary of experimental settings used for empirical validation of our method. Here, the symbol '$\surd$' indicates the algorithm input included as observed data or prior information in the reconstruction of network topology corresponding to cases of BEP_1–BEP_4 (see Table 2 in the main text), respectively.

Algorithm Input	Empirical DAT	Synthesised DAT	Empirical WTD
BEP_1	$\surd$		
BEP_2		$\surd$	
BEP_3	$\surd$		$\surd$
BEP_4		$\surd$	$\surd$

Empirical DAT: Diffusion data of the cascades realized on empirical contact sequences $\{(u_i, v_i, t_i)\}$ of temporal network $\mathcal{N}$
Synthesised DAT: Diffusion data of the cascades synthesised on the STN $\mathcal{N}_\mathrm{s} = (\mathcal{G}, \rho)$ fitted to empirical contact sequences
Empirical WTD: Population-wide, empirical distribution of waiting times that are read off from empirical contact sequences

Supplementary Table 5: Summary of critical sample sizes for different reconstruction goals. The benchmark tests are carried out using for a variety of time-aggregated networks in combination with several different types of benchmark WTDs. The black subscripted θ's represent the minimal sample sizes that assure 0.95 AUROC, AUPR and BEP, respectively. The entries distinguished in cyan colour correspond to the cases of implicit WTDs, and θ_{WTD} presents the critical sample size for WTD recovery (see Supplementary Figure 6 for definition). Here, N is the network size, L is the link number, $\langle k \rangle$ is the average degree, and the (relative) sample size ($\equiv C/N$) denotes the number of diffusion cascades divided by network size. Each critical sample size is obtained by averaging over 10 independent realizations. The dash symbol '$-$' indicates failure in achieving the recovery goals in our numerical experiments using $C/N \in (0, 3]$. All the results are obtained under a homogeneous population assumption that all time-aggregated links produce i.i.d. waiting times, and flat priors are used for avoidance of nuisance parameters. Other parameters: kernel bandwidth $h = 0.05$, WTD support $l_\tau = 8$, and error threshold $\epsilon = 10^{-4}$. More details on the reconstruction performance as a function of sample size for different cases can be found in Supplementary Figure 8–19.

Networks	N	L	$\langle k \rangle$	Gaussian	Weibull	Uniform	Gumbel	Beta	Bimodal	Pareto	J-shape	Exp
							$\begin{pmatrix} \theta_{.95\text{AUROC}} / \theta_{.95\text{AUPR}} \\ \theta_{.95\text{BEP}} / \theta_{\text{WTD}} \end{pmatrix}$					
Lattice 2D	100	180	3.6	0.03 / 0.07<br>0.08 / 0.10	0.03 / 0.08<br>0.12 / 0.10	0.04 / 0.07<br>0.08 / 0.09	0.03 / 0.06<br>0.09 / 0.10	0.06 / 0.10<br>0.13 / 0.21	0.04 / 0.11<br>0.15 / 0.15	0.04 / 0.08<br>0.11 / 0.11	0.05 / 0.08<br>0.10 / 0.17	0.04 / 0.13<br>0.18 / 0.20
Sierpinski	123	243	4.0	0.04 / 0.09<br>0.11 / 0.11	0.02 / 0.07<br>0.10 / 0.07	0.03 / 0.08<br>0.11 / 0.11	0.03 / 0.07<br>0.10 / 0.09	0.05 / 0.09<br>0.15 / 0.20	0.04 / 0.09<br>0.14 / 0.11	0.02 / 0.05<br>0.08 / 0.07	0.04 / 0.11<br>0.16 / 0.21	0.02 / 0.06<br>0.12 / 0.09
Pseudofractal	123	243	4.0	0.10 / 0.23<br>0.48 / 0.24	0.20 / 0.36<br>0.58 / 0.59	0.09 / 0.22<br>0.39 / 0.29	0.10 / 0.24<br>0.34 / 0.30	0.07 / 0.23<br>0.32 / 0.28	0.14 / 0.27<br>0.37 / 0.53	0.11 / 0.20<br>0.27 / 0.38	0.09 / 0.17<br>0.31 / 0.30	0.17 / 0.42<br>0.54 / 0.68
Apollonian	124	366	5.9	0.13 / 0.29<br>0.63 / 0.31	0.24 / 0.49<br>0.67 / 0.82	0.13 / 0.31<br>0.53 / 0.40	0.17 / 0.36<br>0.54 / 0.42	0.11 / 0.25<br>0.54 / 0.34	0.27 / 0.52<br>0.77 / 0.90	0.18 / 0.33<br>0.45 / 0.54	0.11 / 0.27<br>0.48 / 0.46	0.30 / 0.68<br>0.85 / 1.27
Random	100	291	5.8	0.08 / 0.14<br>0.20 / 0.20	0.16 / 0.29<br>0.40 / 0.42	0.10 / 0.17<br>0.24 / 0.23	0.11 / 0.20<br>0.26 / 0.26	0.07 / 0.13<br>0.18 / 0.20	0.21 / 0.41<br>0.58 / 0.61	0.16 / 0.26<br>0.31 / 0.39	0.10 / 0.16<br>0.23 / 0.27	0.30 / 0.51<br>0.67 / 0.92
Small-world	100	300	6.0	0.05 / 0.10<br>0.18 / 0.14	0.08 / 0.19<br>0.33 / 0.25	0.06 / 0.11<br>0.17 / 0.17	0.07 / 0.13<br>0.20 / 0.18	0.07 / 0.11<br>0.18 / 0.15	0.12 / 0.28<br>0.43 / 0.41	0.10 / 0.18<br>0.28 / 0.24	0.06 / 0.13<br>0.17 / 0.20	0.15 / 0.33<br>0.52 / 0.52
Scale-free	100	291	5.8	0.08 / 0.17<br>0.24 / 0.24	0.20 / 0.34<br>0.49 / 0.49	0.11 / 0.21<br>0.26 / 0.29	0.14 / 0.24<br>0.32 / 0.31	0.08 / 0.17<br>0.24 / 0.23	0.28 / 0.52<br>0.67 / 0.74	0.17 / 0.29<br>0.35 / 0.45	0.10 / 0.21<br>0.26 / 0.35	0.33 / 0.62<br>0.74 / 1.02
Karate	34	78	4.6	0.15 / 0.30<br>0.55 / 0.24	0.31 / 0.66<br>1.06 / 0.76	0.20 / 0.37<br>0.64 / 0.32	0.21 / 0.38<br>0.65 / 0.42	0.15 / 0.27<br>0.51 / 0.24	0.41 / 0.71<br>1.17 / 1.04	0.30 / 0.47<br>0.72 / 0.72	0.15 / 0.31<br>0.59 / 0.34	0.49 / 0.91<br>1.49 / 1.38
Dolphins	62	159	5.1	0.07 / 0.17<br>0.36 / 0.15	0.10 / 0.42<br>0.55 / 0.40	0.08 / 0.20<br>0.39 / 0.18	0.10 / 0.24<br>0.40 / 0.20	0.10 / 0.25<br>0.28 / 0.19	0.15 / 0.44<br>0.79 / 0.58	0.12 / 0.27<br>0.43 / 0.31	0.08 / 0.22<br>0.33 / 0.24	0.23 / 0.56<br>0.95 / 0.76
Miserables	77	254	6.6	0.10 / 0.38<br>0.73 / 0.24	0.17 / 0.42<br>1.18 / 0.58	0.12 / 0.30<br>0.64 / 0.32	0.12 / 0.31<br>0.58 / 0.34	0.10 / 0.29<br>0.53 / 0.24	0.16 / 0.54<br>0.97 / 0.62	0.13 / 0.30<br>0.55 / 0.39	0.09 / 0.33<br>0.58 / 0.32	0.18 / 0.95<br>1.10 / 0.85
Football	115	613	10.7	0.08 / 0.17<br>0.27 / 0.20	0.15 / 0.39<br>0.73 / 0.51	0.10 / 0.21<br>0.33 / 0.26	0.10 / 0.22<br>0.37 / 0.28	0.08 / 0.14<br>0.23 / 0.18	0.24 / 0.63<br>1.00 / 0.76	0.13 / 0.30<br>0.46 / 0.40	0.10 / 0.26<br>0.39 / 0.35	0.33 / 0.73<br>1.18 / 1.33
Jazz	198	2742	27.7	0.13 / 0.38<br>0.81 / 0.31	0.32 / 1.41<br>– / 1.09	0.16 / 0.45<br>0.97 / 0.39	0.16 / 0.46<br>1.00 / 0.35	0.11 / 0.28<br>0.56 / 0.24	0.53 / –<br>– / 1.66	0.19 / 0.56<br>1.12 / 0.62	0.44 / 1.78<br>– / 1.48	0.83 / –<br>– / –

Supplementary Table 6: Summary of notations used in the paper.

Notations	Description
$\mathcal{N}$	The temporal network
$\mathcal{V}$	The set of nodes in the given temporal network
$\mathcal{E}$	The set of events (temporal links) in the given temporal network
N	The number of nodes in the given (temporal) network, $N = \#\mathcal{V}$
L	The number of links in the given (temporal) network, $L = \#\mathcal{E}$
$\langle k \rangle$	The average node degree of the time-aggregated network
$\boldsymbol{\psi}$	The set of IETs of the given temporal network, $\boldsymbol{\psi} = [\psi_{uv}]_{(u,v)\in\mathcal{G}}$
ψ_{uv}	The dyad-specific IET on the link (u, v) in the given temporal network
$\mathcal{N}_\mathrm{s}$	The STN built on the given temporal network, $\mathcal{N}_\mathrm{s} = (\mathcal{G}, \boldsymbol{\rho})$
$\mathcal{G}$	The time-aggregated graph of the given STN
$\mathcal{I}_v$	The set of in-coming neighbours of node v, $\mathcal{I}_v = \{u \in \mathcal{V} : (u, v) \in \mathcal{G}\}$
$\boldsymbol{\rho}$	The set of WTDs of the given STN, $\boldsymbol{\rho} = [\rho_{uv}]_{(u,v)\in\mathcal{G}}$
ρ_{uv}	The dyad-specific WTD on link (u, v) in the given STN
τ_{uv}	The waiting time occurred on link (u, v) in the given STN
ρ	The commonly shared WTD of the given STN within a homogeneous population
$\hat{\boldsymbol{\rho}}^0$	The first WTD guess for initialising the iterative inference procedure
Φ_{uv}	The survival function of the WTD on link (u, v), $\Phi_{uv}(\tau) = \int_\tau^\infty \rho_{uv}(t)dt$
λ_{uv}	The hazard function of the WTD on link (u, v), $\lambda_{uv}(\tau) = \rho_{uv}(\tau)/\Phi_{uv}(\tau)$
Λ_v	The cumulative hazard rate of node v to be informed, $\Lambda_v(t) = \sum_{u\in\mathcal{I}_v} \lambda_{uv}(t - t_u)$
L^{in}	The weighted in-degree Laplacian matrix associated with cascade $\mathcal{D}$ on $\mathcal{N}_\mathrm{s}$
$L^{\mathrm{in}}_{s^*}$	The principal minor of Laplacian L^{in} obtained by deleting the s^*-th row and s^*-th column
s^*	The information source that initialises a single diffusion cascade at time 0, $t_{s^*} = 0$
$\mathcal{J}$	The observed trajectory of unicast-type diffusion process, $\mathcal{J} = [v_i]_{i=1}^C$
$\mathcal{D}$	The single diffusion on the given temporal network, $\mathcal{D} = [t_v]_{v\in\mathcal{G}}$
t_v	The DAT at node v in the single diffusion cascade
d_{uv}	The TDOA between nodes u and v in the single diffusion cascade, $d_{uv} = t_v - t_u$
$\mathcal{T}$	The diffusion tree associated with the observed cascade $\mathcal{D}$ on the given STN $\mathcal{N}_\mathrm{s}$
$\boldsymbol{\chi}$	The set of binary branch indicators given cascade $\mathcal{D}$ on $\mathcal{N}_\mathrm{s}$, $\boldsymbol{\chi} = [\chi_{uv}(\mathcal{D}, \mathcal{N}_\mathrm{s})]_{(u,v)\in\mathcal{G}}$
χ_{uv}	The binary branch indicator of link (u, v) given cascade $\mathcal{D}$ on $\mathcal{N}_\mathrm{s}$, $\chi_{uv}(\mathcal{D}, \mathcal{N}_\mathrm{s}) = \mathbb{1}_{[(u,v)\in\mathcal{T}]}$
$p^*_\mathcal{T}$	The likelihood weighing of diffusion trees, in the decorrelated case, $p^*_\mathcal{T} = \prod_{(u,v)\in\mathcal{T}} \beta_{uv}(\mathcal{D}, \mathcal{N}_\mathrm{s})$
β_{uv}	The branching coefficient of link (u, v) given cascade $\mathcal{D}$ on $\mathcal{N}_\mathrm{s}$, $\beta_{uv}(\mathcal{D}, \mathcal{N}_\mathrm{s}) = \lambda_{uv}(d_{uv})/\Lambda_v(t_v)$
$\bar{\beta}_{uv}$	The complementary probability of β_{uv} given cascade $\mathcal{D}$ on $\mathcal{N}_\mathrm{s}$, $\bar{\beta}_{uv}(\mathcal{D}, \mathcal{N}_\mathrm{s}) = 1 - \beta_{uv}(\mathcal{D}, \mathcal{N}_\mathrm{s})$
$\rho_{uv,vw}$	The bivariate WTD for adjacent links $(u, v), (v, w) \in \mathcal{G}$ in the second-order STN model
$\rho_{vw\vert uv}$	The conditional WTD for adjacent links $(u, v), (v, w) \in \mathcal{G}$ in the second-order STN model
$\beta_{uv,vw}$	The second-order branching coefficient $\beta_{uv,vw}(\mathcal{D}, \mathcal{N}_\mathrm{s}) = \Pr\left[(u, v) \in \mathcal{T}, (v, w) \in \mathcal{T} \vert \mathcal{D}, \mathcal{N}_\mathrm{s}\right]$
$\beta_{uv,\overline{vw}}$	The second-order branching coefficient $\beta_{uv,\overline{vw}}(\mathcal{D}, \mathcal{N}_\mathrm{s}) = \Pr\left[(u, v) \in \mathcal{T}, (v, w) \notin \mathcal{T} \vert \mathcal{D}, \mathcal{N}_\mathrm{s}\right]$
$\beta_{vw\vert uv}$	The conditional branching coefficient $\beta_{vw\vert uv}(\mathcal{D}, \mathcal{N}_\mathrm{s}) = \Pr\left[(v, w) \in \mathcal{T} \vert (u, v) \in \mathcal{T}, \mathcal{D}, \mathcal{N}_\mathrm{s}\right]$

(continued on next page)

Supplementary Table 6: Notations used in the paper (continued)

Notations	Description
C	The number of observed diffusion cascades, $C = \#D$
C/N	The relative sample size of observed diffusion cascades
T^w	The length of observation window
D	The set of observed diffusion cascades, $D = \{\mathcal{D}^i\}_{i=1}^C$
$\mathcal{D}^i$	The i-th cascade of observed diffusion, $\mathcal{D}^i = [t_v^i]_{v\in\mathcal{G}}$
t_v^i	The DAT at node v in the i-th cascade of observed diffusion
d_{uv}^i	The TDOA between nodes u and v in the i-th cascade of observed diffusion, $d_{uv}^i = t_v^i - t_u^i$
$\theta_{\bullet}$	The minimal C/N for achieving different reconstruction goals (see Supplementary Table 5)
$L(\mathcal{D}\|\mathcal{N}_{\mathrm{s}})$	The likelihood functional of the observed diffusion cascade $\mathcal{D}$ on $\mathcal{N}_{\mathrm{s}}$
$\ell(\mathcal{D}\|\mathcal{N}_{\mathrm{s}})$	The logarithmic likelihood functional of the observed diffusion cascade $\mathcal{D}$ on $\mathcal{N}_{\mathrm{s}}$
$L_{\mathcal{A}}(\mathcal{D}\|\mathcal{N}_{\mathrm{s}})$	The likelihood functional of the observed diffusion cascade $\mathcal{D}$ on $\mathcal{N}_{\mathrm{s}}$ with a taboo set $\mathcal{A}$ of links
$\Delta_{uv}\ell$	The marginal gain of the link flipping operation
K_h	The Gaussian kernel smoother, $K_h(\tau) = \frac{1}{\sqrt{2\pi h^2}}\exp(-\tau^2/2h^2)$
h	The kernel bandwidth
ξ	The entropic inferential complexity
H	The Shannon entropy
I	The mutual information
$\hat{H}$	The estimated Shannon entropy using data of empirical temporal networks
$\hat{I}$	The estimated mutual information using data of empirical temporal networks
M	The number of MCMC samples used in the topology inference step, $M = \#\hat{\mathbf{G}}$
$\hat{\mathbf{G}}$	The set of MCMC samples of the time-aggregated network configurations
$\hat{q}_{uv}$	The posterior presence probability of link (u,v) in $\hat{\mathcal{G}}$ obtained by MCMC sampling
θ	The threshold value used in the thresholding process to prune links, $\hat{\mathcal{G}} = \{(u,v) : p_{uv} > \theta\}$
K	The order of truncated Fourier series
ρ^K	The approximate WTD using the truncated Fourier series of order K
c_{uv}^n	The n-th Fourier coefficient of ρ
k_h^n	The n-th Fourier coefficient of K_h
l_τ	The support of the WTD
B	The bootstrap resample size
α	The bootstrap confidence level
β	The hyperparameter in the sparsity prior
γ	The skewness of WTD
μ	The mean of the WTD
δ^2	The variance of the WTD
δt	The duration of temporal events/the discretisation size of WTDs (ideally, $\delta t \to 0$)
ϵ	The error threshold used for judgement of convergence
π	The sparsity prior, $\pi(\mathcal{G}) \propto e^{-\beta\|\mathcal{G}\|_1}$

(continued on next page)

Supplementary Table 6: Notations used in the paper (continued)

Notations	Description
$\boldsymbol{\theta}$	The set of PDF parameters of the WTD, $\boldsymbol{\theta} = \{a, b\}$
a	The location parameter of the WTD
b	The shape parameter of the WTD
T	The vector of RVs denoting DATs of network nodes, $T = [T_v]_{v \in \mathcal{V}}$
T_v	The RV denoting DAT of node v
r_{uv}	The residue probability mass of $\rho_{uv}(\tau)$ located at the infinity, $r_{uv} = 1 - \Phi_{uv}(0)$
$\mathcal{G} \cup uv$	The network configuration obtained by adding link (u, v) to $\mathcal{G}$
$\mathcal{G} \setminus uv$	The network configuration obtained by removing link (u, v) from $\mathcal{G}$
$\Phi^{-1}(\alpha)$	The $(1-\alpha)$-quantile of WTD, Φ^{-1} is the inverse survival function of the WTD ρ
$e_1 \prec e_2$	The relation representing (both temporally and topologically) successive links $e_1, e_2 \in \mathcal{E}$
π_{uv}	The weights used for calculation of DSD of empirical datasets, $\pi_{uv} = \#\{(e_1, e_2) \in \mathcal{E}^2 : e_1 \prec e_2\}$
(x_i, y_i)	The paired waiting times on successive temporal links used for calculation of PCC and NMI
DSD	The distributional standard deviation
PCC	The Pearson correlation coefficient
NMI	The normalised mutual information
BEP	The precision/recall of the optimal topology estimate attained at the break-even point
TPR	The true positive rate
FPR	The false positive rate
AUROC	The area under the receiver operating characteristic curve
AUPR	The area under the precision-recall curve
$\|\cdot\|_1$	The ℓ_1 norm, $\|\mathcal{G}\|_1 = \#\{(u, v) \in \mathcal{G}\}$
$\|\cdot\|_p$	The p-norm
$\|\cdot\|_{\mathrm{KS}}$	The Kolmogorov-Smirnov divergence
$\langle\cdot\rangle_D$	The average taken over the ensemble of diffusion cascades
$\langle\cdot,\cdot\rangle$	The inner product defined on the (WTD) functional space support l_τ, $\langle\rho, \rho'\rangle = \int_0^{l_\tau} \rho(\tau)\rho'(\tau)d\tau$
$\mathbb{1}_{[\cdot]}$	The indicator function
$\delta(\cdot)$	The Dirac delta function
$\Theta(\cdot)$	The Heaviside unit-step function
$\Gamma(\cdot)$	The Euler gamma function
$\Pr(\cdot)$	The probability of the given event
$p(\cdot)$	The probability density of the given event
$\#$	The set cardinality
$\otimes$	The convolution operation
$\mathbb{E}$	The conditional expectation
$\hat{a}$	The estimated quantity a using observed data, a standing for $\mathcal{G}$, ρ, BEP, etc.
$a[\cdot]$	The discretised version of quantity a based on the binned WTD, a standing for ρ, Φ, λ, β_{uv}, etc.
$\tilde{a}$	The estimated quantity a using bootstrap samples, a standing for $\mathcal{G}$, ρ, etc.

Supplementary Note 1: Inverse modelling of temporal networks with STN null models

In this Supplementary Note, we first give a brief review on the related literature, and then justify the stochastic temporal network (STN) used as a null model in the time-extended inverse problem of network reconstruction.

Despite many systematic approaches to data-driven reconstruction of static networks [22], this inverse problem extending to high temporal resolution remains a nontrivial challenge. The increased ill-posedness manifests itself in detailing the extra time stamps of the internal links. A temporal network, characterised by its time variability in interaction patterns, can be defined in a variety of ways. One of the widely used representation is a set of adjacency matrices $\{A_t\}_{t\in T}$ that describe the snapshots of interactions at a series of equidistant epochs indexed by T [23]. Noticeably, the conventional methods for topology discovery, though applicable to each epoch respectively, fail to consider the temporal network as a whole, leaving little hope for meaningful inference in this context of time-varying networked systems. An intriguing but somewhat speculative idea, originated in sociology and statistics, postulates that the time evolution of A_t is governed by a graph-valued Markov process with transition kernel described in terms of a few parameters that can be learned empirically. This leads to a useful extension of the exponential random graph model [24] and other variants [25] for modelling temporal social networks. Another thread of ideas, mainly from the bioinformatics literature, is regression based [26], where A_t typically enters an ℓ_1-regularised logistic regression [27] or an autoregressive process [28] for some time series of experimentally measured (e.g., protein-protein interaction and gene expression) data and is estimated through for example, convex optimisation techniques. However, these methods commonly depend on *ad hoc* mechanisms as well as on parameterisation details to account for the time-evolving networked interactivity underlying the relevant social and biological processes.

In this study, we abandon the use of serial snapshots that can only be inferred under strong and arguably unrealistic assumptions, such as Markovianity and temporal smoothness criteria. Instead, we restrict on reconstructing a class of stochastic temporal networks (STNs)—which can be generally taken as a null model preserving temporal statistics for all dyads of interacting individuals but ignoring higher-order correlations across them—to circumvent the complexity in recovering actual snapshots.

The STN model has several other merits, of which we list two. First, under the framework of STNs, attention is shifted away from the specifics of time-stamped interactions to estimating their IET distributions which play an intrinsic role in shaping the dynamical behaviour of temporal networks. However, reading off IET distributions is far from straightforward even with complete knowledge of exact timestamps of the recurrent events. This is because the observable data are often window-censored (due to, e.g., finite-size effects [29]), a situation routinely encountered in survival analysis [30]. One of our main goals here is to provide a route to address the censored nature of indirect measurements in the context of inverse problems. Second, the STN model offers a minimal generative paradigm for temporal networks, the reconstruction of which is naturally partitioned into subtasks concerning structural and temporal aspects of the latent network separately, as we shall specify later on. This motivates an iterative coordinate-ascent inference procedure alternating between an step of topology detection for the underlying time-aggregated network and an step of density estimation for the dyad-specific IET distributions (or usually their length-biased versions). We note here that our inferential approach is complementary and not in competition with the many efficient reconstruction techniques for static networks in the sense that the relevant heuristics (e.g., sparsity optimisation [31, 32]) are also applicable to our topology discovery stage.

Basis and philosophy of our inferential method.— The proposed modelling method for STNs based on the arrival time series of diffusion is to some extent intuitive and pragmatic. In the respect of topology inference, the idea originates from the fact that the time-aggregated graph of the original temporal networks can be directly recovered, at least in the asymptotic regime of infinite process data, using the superposed spreading paths encoded in the observable arrival order of the networked diffusion. Interpreted more narrowly in the literature of random-walk exploration of networks [33], the time-aggregated graph $\mathcal{G}$ is straightforwardly estimated as

$$\hat{\mathcal{G}}(\mathcal{J}) = \bigcup_{i=1}^{l} (v_i^f, v_i), \tag{1}$$

where $\mathcal{J} = \{v_0, v_1, \ldots, v_l\}$ is the unicast path of length l consisting of nodes visited by the random walks in ascending time order, and (v_i^f, v_i) denotes the branch along which diffusion has actually taken place. Here v_i^f is determinable according to the recorded trajectory, thus being v_{i-1}.

Our primary interest in this paper is to extend this intuition to handle the more nontrivial case of cascading dynamics where diffusion proceeds in a broadcast manner. Part of the challenge stems from the unobservability of actual diffusion branches. It is often confounded by simultaneity of exposure of v_i to competing diffusive routes [i.e., $(v_j, v_i) \in \mathcal{G}$ such that $j < i$], thus causing complicated censoring patterns in a networked environment. The key to our method is a probabilistic treatment of v_i^f as being distributed over all possible "upstream" nodes that precede v_i in the cascade. Here we call these route-specific probabilities branching coefficients. They play a pivotal role in the topology inference step for our inverse modelling procedure, one important consequence being that their Shannon entropy offers a natural metric to capture the "degree of complexity" in diagnostics of diffusion pathways underlying the observed cascades over temporal networks (see Supplementary Note 11).

The further utility of using branching coefficients, on the other hand, is that they serve as a "soft" rather than sharp censoring indicator specialised for inferring statistical temporal properties of underlying interactions at the dyadic level. It is intended to formalize a fully consistent and regression-free approach to extracting the so-called waiting-time distributions (WTDs), a reflection of length-biased sampling of IETs frequently encountered in the literature. Specifically, we construct a set of first-principles, self-consistent equations for WTDs, and estimate them iteratively starting with an automated data-based distribution guess. In particular, we resort to kernel smoothing techniques [34] extensively used for density estimation in a broad variety of nonparametric settings, and unsupervised choice of the smoothing parameters is also considered to facilitate data-driven reconstruction of STNs.

Supplementary Note 2: Derivation of likelihood functional and branching coefficient

Derivation of likelihood functional.—To simplify the likelihood computation [equation (2) in the main text], we make use of the survival and hazard functions of WTDs [30], $\Phi_{uv}(\tau) = \int_{\tau}^{\infty} \rho_{uv}(t)dt$ and $\lambda_{uv}(\tau) = \rho_{uv}(\tau)/\Phi_{uv}(\tau)$, thus yielding

$$L(\mathcal{D}|\mathcal{N}_{\mathrm{s}}) = \big[\sum_{\mathcal{T}\subset\mathcal{G}} \prod_{(u,v)\in\mathcal{T}} \lambda_{uv}(d_{uv})\big] \prod_{(u,v)\in\mathcal{G}} \Phi_{uv}(d_{uv}). \tag{2}$$

To calculate the bracketed term, applying the Weighted Matrix-Tree Theorem [35] gives

$$\sum_{\mathcal{T}\subset\mathcal{G}} \prod_{(u,v)\in\mathcal{T}} \lambda_{uv}(d_{uv}) = \det L^{\mathrm{in}}_{s^*}, \tag{3}$$

where $L^{\mathrm{in}}_{s^*}$ is the principal minor of the weighted in-degree Laplacian matrix L^{in} obtained by deleting the s^*-th row and s^*-th column. More specifically, the matrix L^{in} has $\Lambda_i(\mathcal{D}|\mathcal{N}_{\mathrm{s}}) = \sum_{(j,i)\in\mathcal{G}} \lambda_{ji}(d_{ji})$ as the i-th diagonal entry and $l_{ij}(\mathcal{D}|\mathcal{N}_{\mathrm{s}}) = -\mathbb{1}_{[(j,i)\in\mathcal{G}]}\lambda_{ji}(d_{ji})$ as the (i,j)-th off-diagonal entry, where $\mathbb{1}_{[\cdot]}$ denotes the indicator function. Because L^{in} is triangular up to permutation similarity, we obtain

$$L(\mathcal{D}|\mathcal{N}_{\mathrm{s}}) = \prod_{v\in\mathcal{V}\setminus s^*} \Lambda_v(\mathcal{D}|\mathcal{N}_{\mathrm{s}}) \prod_{(u,v)\in\mathcal{G}} \Phi_{uv}(d_{uv}). \tag{4}$$

Taking logarithm of both sides yields

$$\ell(\mathcal{D}|\mathcal{N}_{\mathrm{s}}) = \sum_{v\in\mathcal{V}\setminus s^*} \log \Lambda_v(\mathcal{D}|\mathcal{N}_{\mathrm{s}}) + \sum_{(u,v)\in\mathcal{G}} \log \Phi_{uv}(d_{uv}), \tag{5}$$

which is the same as equation (3) in the main text. Note that it is technically simpler to consider logarithmic likelihood functional $\ell(\mathcal{D}|\mathcal{N}_{\mathrm{s}})$, whose maximum falls in the same place.

Derivation of branching coefficient.—The route-specific branching coefficient $\beta_{uv}(\mathcal{D}|\mathcal{N}_{\mathrm{s}}) = \Pr\left[(u,v)\in\mathcal{T}|\mathcal{D},\mathcal{N}_{\mathrm{s}}\right]$ is interpreted as the conditional probability of link (u,v) acting as an diffusion branch given the observed cascade $\mathcal{D}$ on the STN $\mathcal{N}_{\mathrm{s}}$. We first find the complementary probability, $\bar{\beta}_{uv}(\mathcal{D},\mathcal{N}_{\mathrm{s}}) = \Pr[(u,v)\notin\mathcal{T}|\mathcal{D},\mathcal{N}_{\mathrm{s}}]$, that link (u,v) acts instead as a chord in the given cascade $\mathcal{D}$. The conditional probability $\Pr[\mathcal{D},(u,v)\notin\mathcal{T}|\mathcal{N}_{\mathrm{s}}]$ is obtained by the probability of (u,v) producing a censored waiting time d_{uv}, multiplied by the likelihood of observing cascade $\mathcal{D}$ in the configuration $\mathcal{N}_{\mathrm{s}}\setminus uv$ obtained as if (u,v) were removed from the time-aggregated graph $\mathcal{G}$ as a cavity link, namely,

$$\begin{aligned}\bar{\beta}_{uv}(\mathcal{D},\mathcal{N}_{\mathrm{s}}) &= \frac{\Pr\left[T=\mathcal{D},(u,v)\notin\mathcal{T}|\mathcal{N}_{\mathrm{s}}\right]}{\Pr\left[T=\mathcal{D}|\mathcal{N}_{\mathrm{s}}\right]} = \frac{\Pr\left[T=\mathcal{D}|(u,v)\notin\mathcal{T},\mathcal{N}_{\mathrm{s}}\right]\Pr\left[\tau_{uv}>d_{uv}|\mathcal{N}_{\mathrm{s}}\right]}{\Pr\left[T=\mathcal{D}|\mathcal{N}_{\mathrm{s}}\right]} \\ &= \frac{L(\mathcal{D}|\mathcal{N}_{\mathrm{s}}\setminus uv)\Phi_{uv}(d_{uv})}{L(\mathcal{D}|\mathcal{N}_{\mathrm{s}})} = \frac{\Lambda_v(\mathcal{D}|\mathcal{N}_{\mathrm{s}}\setminus uv)}{\Lambda_v(\mathcal{D}|\mathcal{N}_{\mathrm{s}})},\end{aligned} \tag{6}$$

where $T = \{T_v\}_{v\in\mathcal{V}}$ is the vector of random variables (RVs) denoting the DATs of network nodes in our generative model, and $\Lambda_v(\mathcal{D}|\mathcal{N}_{\mathrm{s}}) = \sum_{(u,v)\in\mathcal{G}} \lambda_{uv}(d_{uv})$ is the v-th diagonal entry of the weighted in-degree

Laplacian matrix L^{in} corresponding to the observed cascade $\mathcal{D}$ on $\mathcal{N}_{\text{s}}$. Therefore, we have

$$\beta_{uv}(\mathcal{D},\mathcal{N}_{\text{s}}) = 1-\bar{\beta}_{uv}(\mathcal{D},\mathcal{N}_{\text{s}}) = \frac{\lambda_{uv}(d_{uv})}{\sum_{(w,v)\in\mathcal{G}}\lambda_{wv}(d_{wv})}, \tag{7}$$

which is the same as equation (4) in the main text.

Remark.—To illuminate the role of branching coefficients in topology inference, we consider a hypothetical situation allowing direct observation of when and by whom an individual is informed during the cascade, that is, not only can we observe the cascade of DATs $\mathcal{D}^i$, but the associated diffusion tree $\mathcal{T}^i$ as well. Thus, $\beta_{uv}(\mathcal{D}^i,\hat{\mathcal{G}},\boldsymbol{\rho})$ becomes a binary indicator $\chi^i_{uv} = \mathbb{1}_{[(u,v)\in\mathcal{T}^i]}$ for diffusion branches. We denote by $\hat{\mathcal{G}}(D,\boldsymbol{\rho},\boldsymbol{\chi})$ with $\boldsymbol{\chi} = [\chi^i_{uv}]_{u,v\in\mathcal{V},i\in\{1,\dots,C\}}$ the ML estimate of the time-aggregated network from observed diffusion cascades. For any link $(u,v)\in\hat{\mathcal{G}}(D,\boldsymbol{\rho},\boldsymbol{\chi})$, if there is at least a cascade $\mathcal{D}^i\in D$ such that $\chi^i_{uv}=1$, then the marginal gain of removing link (u,v) is $\Delta_{uv}\ell(D|\hat{\mathcal{G}},\boldsymbol{\rho}) = -\infty$, thus leading to a zero acceptance probability $p_{uv}=0$ for the flipping operation on link (u,v); and otherwise, adding (u,v) to $\hat{\mathcal{G}}$ definitely causes a decreased likelihood [noting that $\Delta_{uv}\ell(D|\hat{\mathcal{G}},\boldsymbol{\rho}) = \sum_{i=1}^{C}\log\Phi(d^i_{uv})<0$] and hence (u,v) must be excluded from the ML configuration. Thus, we simply obtain

$$\hat{\mathcal{G}}(D,\boldsymbol{\rho},\boldsymbol{\chi}) = \bigcup_{i=1}^{C}\left\{(u,v):\chi^i_{uv}=1\right\} = \bigcup_{i=1}^{C}\mathcal{T}^i. \tag{8}$$

It is intuitively clear that the ML topology corresponds to union of observed diffusion trees from the respective cascades.

In the more general case of implicit diffusion structure, this observable dichotomy is in general missing [the only exception is $\chi^i_{uv}=1$, for the first diffusion branch (u,v) in cascade $\mathcal{D}^i$, where node u is the information source of the diffusion, and node v is the first neighbour of u to be informed]. The proposed Gibbs sampling algorithm for topology inference $\hat{\mathcal{G}}(D,\boldsymbol{\rho})$ can therefore be taken as an expectation-maximisation version of $\hat{\mathcal{G}}(D,\boldsymbol{\rho},\boldsymbol{\chi})$, since the branching coefficients $\beta_{uv}(\mathcal{D}^i,\hat{\mathcal{G}},\boldsymbol{\rho})$ we used in place of χ_{uv} satisfy

$$\beta_{uv}(\mathcal{D}^i,\hat{\mathcal{G}},\boldsymbol{\rho}) = \Pr\left[(u,v)\in\mathcal{T}^i\middle|\mathcal{D}^i,\hat{\mathcal{G}},\boldsymbol{\rho}\right] = \mathbb{E}\left[\chi^i_{uv}\middle|\mathcal{D}^i,\hat{\mathcal{G}},\boldsymbol{\rho}\right], \tag{9}$$

where $\mathbb{E}[\cdot]$ denotes the conditional expectation. Also see the remark at the end of Supplementary Note 5 for discussion of the role of branching coefficients in the density estimation step.

A direct derivation.—At the end of this Supplementary Note, we provide another direct calculation of $L(\mathcal{D}|\mathcal{N}_{\text{s}})$ and $\beta_{uv}(\mathcal{D},\mathcal{N}_{\text{s}})$, rather than using Matrix-Tree Theorem of weighted graphs or Superposition Theorem of point processes.

First, by the chain rule of probability, the likelihood of observing a single diffusion cascade $\mathcal{D}=[t_v]_{v\in\mathcal{V}}$ is

$$\begin{aligned} L(\mathcal{D}|\mathcal{N}_{\text{s}}) =& p(T=\mathcal{D}|T_{s^*}=t_{s^*}) \\ =& p(T_{v_1}=t_{v_1}|T_{s^*}=t_{s^*})p(T_{v_2}=t_{v_2}|T_{s^*}=t_{s^*},T_{v_1}=t_{v_1})\cdots p(T_{v_n}=t_{v_n}|T_{s^*}=t_{s^*},\dots,T_{v_{n-1}}=t_{v_{n-1}}), \end{aligned} \tag{10}$$

where $p(\cdot)$ denotes the conditional probability density, and $\{s^*, v_1, \dots, v_n\}$ is the node list in an increasing order according to their DATs in the given cascade $\mathcal{D}$. Here we need to calculate the probability of node v_i's DAT falling within an infinitesimal interval dt, $T_{v_i} \in [t_{v_i}, t_{v_i} + dt)$, conditioning on the preceding DATs $\{T_v = t_v\}_{v \in \mathcal{J}_{v_i}}$ with $\mathcal{J}_{v_i} = \{s^*, v_1, \dots, v_{i-1}\}$. Due to the indeterminacy on the diffusion tree $\mathcal{T}$ underlying the realised cascade, this conditional probability considers all possible neighbours of node v_i being the first to inform v_i at time t_{v_i} and is written

$$
\begin{aligned}
p(T_{v_i} = t_{v_i} | \{T_v = t_v\}_{v \in \mathcal{J}_{v_i}}) dt &= \sum_{u \in \mathcal{I}_{v_i} \cap \mathcal{J}_{v_i}} p(T_{v_i} = t_{v_i}, (u, v_i) \in \mathcal{T} | \{T_v = t_v\}_{v \in \mathcal{J}_{v_i}}) dt \\
&= \sum_{u \in \mathcal{I}_{v_i} \cap \mathcal{J}_{v_i}} \Pr\left[t_u + \tau_{uv_i} \in [t_{v_i}, t_{v_i} + dt)\right] \prod_{\substack{u' \neq u \\ u' \in \mathcal{I}_{v_i} \cap \mathcal{J}_{v_i}}} \Pr\left[t_{u'} + \tau_{u'v_i} > t_{v_i}\right] \\
&= \sum_{u \in \mathcal{I}_{v_i} \cap \mathcal{J}_{v_i}} \rho_{uv_i}(t_{v_i} - t_u) dt \prod_{\substack{u' \neq u \\ u' \in \mathcal{I}_{v_i} \cap \mathcal{J}_{v_i}}} \int_{t_{v_i} - t_{u'}}^{\infty} \rho_{u'v_i}(\tau) d\tau \\
&= \left[\sum_{u \in \mathcal{I}_{v_i} \cap \mathcal{J}_{v_i}} \lambda_{uv_i}(d_{uv_i}) \right] dt \prod_{u \in \mathcal{I}_{v_i} \cap \mathcal{J}_{v_i}} \Phi_{uv_i}(d_{uv_i}).
\end{aligned}
\tag{11}
$$

Note that the event $(u, v_i) \in \mathcal{T}$ implies that any other incident links are chords censored by the diffusion occurred on (u, v_i). Here we used the null model assumption of mutually independent waiting times occurring on STN links. With the convention that $\lambda_{uv}(\tau) = 0$, $\Phi_{uv}(\tau) = 1, \tau < 0$, the above equation further simplifies to

$$
p(T_{v_i} = t_{v_i} | \{T_v = t_v\}_{v \in \mathcal{J}_{v_i}}) = \left[\sum_{u \in \mathcal{I}_{v_i}} \lambda_{uv_i}(d_{uv_i}) \right] \prod_{u \in \mathcal{I}_{v_i}} \Phi_{uv_i}(d_{uv_i}). \tag{12}
$$

Finally, we obtain

$$
L(\mathcal{D} | \mathcal{N}_\mathrm{s}) = \prod_{v \in \mathcal{V} \setminus s^*} \left[\sum_{u \in \mathcal{I}_v} \lambda_{uv}(d_{uv}) \right] \prod_{u \in \mathcal{I}_v} \Phi_{uv}(d_{uv}), \tag{13}
$$

which is consistent with equation (4).

Then using the Bayesian rule gives

$$
\begin{aligned}
\beta_{uv}(\mathcal{D}, \mathcal{N}_\mathrm{s}) = \frac{\Pr\left[T = \mathcal{D}, (u, v) \in \mathcal{T} | \mathcal{N}_\mathrm{s}\right]}{\Pr\left[T = \mathcal{D} | \mathcal{N}_\mathrm{s}\right]} &= \frac{p(T_v = t_v, (u, v) \in \mathcal{T} | T_{s^*} = t_{s^*}, \dots, T_{v_{i-1}} = t_{v_{i-1}})}{p(T_v = t_v | T_{s^*} = t_{s^*}, \dots, T_{v_{i-1}} = t_{v_{i-1}})} \\
= \frac{\lambda_{uv}(d_{uv}) \prod_{u \in \mathcal{I}_v} \Phi_{uv}(d_{uv})}{\left[\sum_{u' \in \mathcal{I}_v} \lambda_{u'v}(d_{u'v})\right] \prod_{u \in \mathcal{I}_v} \Phi_{uv}(d_{uv})} &= \frac{\lambda_{uv}(d_{uv})}{\sum_{u' \in \mathcal{I}_v} \lambda_{u'v}(d_{u'v})},
\end{aligned}
\tag{14}
$$

where $\{s^*, v_1, \dots, v_i\} \subset \mathcal{V}$ are the chronologically ordered sequence of nodes ending with $v_i = v$. Obviously, the branching coefficients of incident links to any informed node v (except the information source s^*) are normalised to unity, $\sum_{u \in \mathcal{I}_v} \beta_{uv} = 1$. Particularly for underlying trees $\mathcal{T}$, $\beta_{uv}(\mathcal{D}, \mathcal{T}) = \Theta(d_{uv})$. Most importantly, we note that the calculation is completely localised $[\beta_{uv}(\mathcal{D}, \mathcal{N}_\mathrm{s}) = \beta_{uv}(\mathcal{D}, \mathcal{I}_v)]$ with the utility of the null model assumption of STNs.

Supplementary Note 3: NP-hardness proof for maximum likelihood topology inference

We first decompose the ML topology inference into a set of subproblems for reconstructing local configurations centered at respective nodes. For brevity, we assume that the sample size is $C = 1$, namely, only a single diffusion cascade of DATs $\mathcal{D} = [t_v]_{v\in\mathcal{V}}$ is observed. Consider the following ML estimation for the time-aggregated topology of STNs [up to a set of non-inferable links (u, v) such that $d_{uv} < 0$]:

$$\begin{aligned}
\hat{\mathcal{G}}(\mathcal{D}, \boldsymbol{\rho}) &= \arg\max_{\mathcal{G}} L(\mathcal{D}|\mathcal{G}, \boldsymbol{\rho}) \\
&= \arg\max_{\mathcal{G}} \left[\sum_{v\in\mathcal{V}} \log \sum_{u\in\mathcal{I}_v} \lambda_{uv}(d_{uv}) + \sum_{(u,v)\in\mathcal{G}} \log \Phi_{uv}(d_{uv})\right] \\
&= \arg\max_{\mathcal{G}} \sum_{v\in\mathcal{V}} \left[\log \sum_{u\in\mathcal{I}_v} \lambda_{uv}(d_{uv}) + \sum_{u\in\mathcal{I}_v} \log \Phi_{uv}(d_{uv})\right] \\
&= \bigcup_{v\in\mathcal{V}} \arg\max_{\mathcal{I}_v\subset\mathcal{V}} \left[\log \sum_{u\in\mathcal{I}_v} \lambda_{uv}(d_{uv}) + \sum_{u\in\mathcal{I}_v} \log \Phi_{uv}(d_{uv})\right],
\end{aligned} \tag{15}$$

where $d_{uv} = t_v - t_u$ represents the TDOA between nodes u and v, $\Phi_{uv}(\tau)$ and $\lambda_{uv}(\tau)$ are the survival and hazard functions of WTD $\rho_{uv}(\tau)$ on link (u, v), respectively. As such, the original task consists of several well-paralleled subproblems, each solving an ML equation with respect to the neighbourhood set $\mathcal{I}_v = \big\{u \in \mathcal{V} : (u, v) \in \mathcal{G}\big\}$ of node v, and then the inferred local configurations $\mathcal{I}_v^*$ together constitute the ML estimate $\hat{\mathcal{G}}$. Here, we slightly abused the notation $\hat{\mathcal{G}} = \bigcup_{v\in\mathcal{V}} \mathcal{I}_v^* = \bigcup_{v\in\mathcal{V}} \big\{(u, v) : u \in \mathcal{I}_v^*\big\}$.

Despite the deceptive simplicity of the ML equation, it belongs to a broad class of submodular function maximisation problems in the literature of combinatorial optimisation, which is, however, in general computationally intractable [36, 37]. For each node $v \in \mathcal{V}$, the subproblem aims to maximizing the following submodular utility function with respect to the local configuration $\mathcal{I}_v$:

$$f(\mathcal{I}_v) = \log \sum_{u\in\mathcal{I}_v} b_u - \sum_{u\in\mathcal{I}_v} c_u, \tag{16}$$

with $b_u = \lambda_{uv}(d_{uv})$ and $c_u = -\log \Phi_{uv}(d_{uv})$. Here, b_u and c_u can be respectively regarded as the "benefit" and "cost" of establishing a connection from nodes u to v. We further assume that $\{b_u\}$ and $\{c_u\}$ are strictly positive, because for any link (u, v) with TDOA $d_{uv} < 0$, we have $b_u = c_u = 0$ and thus $f(\mathcal{I}_v) = f(\mathcal{I}_v \setminus u)$. To verify the submodularity of the utility function [equation (16)], we calculate the marginal gain of adding a new neighbour u to $\mathcal{I}_v$ as follows:

$$\Delta_u f(\mathcal{I}_v) = f(\mathcal{I}_v \cup u) - f(\mathcal{I}_v) = \log\Big(1 + \frac{b_u}{\sum_{u'\in\mathcal{I}_v} b_{u'}}\Big) - c_u, \tag{17}$$

which is monotonically decreasing as the set $\mathcal{I}_v$ that u is added to increases.

NP-hardness proof. Next we show that the subproblem of finding the ML local configuration $\mathcal{I}_v^*$ as a maximiser of equation (16) is NP-hard. The NP-hardness proof is by reduction from PARTITION [38]. Given a set of n

positive numbers, $S = \{a_1, \ldots, a_n\}$, the PARTITION problem is a task of deciding whether there is a subset $Q^* \subset S$ such that $a(Q^*) = a(S \backslash Q^*)$, where $a(Q^*) = \sum_{x \in Q^*} x$ denotes the sum of set elements in Q^*. We first set $a(S) = 2$ by scaling if necessary, and then the PARTITION problem calls for a subset Q^* such that $a(Q^*) = 1$. Consider now a subproblem of inferring the local configuration, $\mathcal{I}_v^* \subset \{u_1, \ldots, u_n\}$ centred at node v, from observed DATs $\{t_{u_1}, \ldots, t_{u_n}\}$, as well as that of the focus node v which is the latest to be informed. We select $\rho_{uv}(\tau)$ from arbitrarily-shaped WTDs properly so that $\lambda_{uv}(d_{uv}) = -\log \Phi_{uv}(d_{uv}) = a_u$, $u = u_1, \ldots, u_n$, as shown in Supplementary Figure 1. Thus the PARTITION task is equivalent to checking whether the ML solution $\mathcal{I}_v^*$ to the original subproblem satisfies that $f(\mathcal{I}_v^*) = -1$, because

$$f(\mathcal{I}_v^*) = \log \sum_{u \in \mathcal{I}_v^*} a_u - \sum_{u \in \mathcal{I}_v^*} a_u = -1 \iff \max_{Q \subset S} \log a(Q) - a(Q) = -1 \iff a(Q^*) = 1, \tag{18}$$

noting that the function $g(x) = \log x - x$ has a unique maximum at $x^* = 1$.

Supplementary Note 4: Performance assessments for the topology inference algorithms

To quantitatively measure the performance of our inference method for underlying time-aggregated networks, here we adopted two standard judgements, the area under the receiver operating characteristic curve (AUROC) and the area under the precision-recall curve (AUPR) [39]. True positive rate (TPR), false positive rate (FPR), precision and recall used for calculation of AUROC and AUPR are, respectively, given by

$$\begin{aligned} \mathrm{TPR}(\theta) &= \frac{\mathrm{TP}(\theta)}{P}, \\ \mathrm{FPR}(\theta) &= \frac{\mathrm{FP}(\theta)}{Q}, \\ \mathrm{Precision}(\theta) &= \frac{\mathrm{TP}(\theta)}{\hat{L}(\theta)} = \frac{\mathrm{TP}(\theta)}{\mathrm{TP}(\theta)+\mathrm{FP}(\theta)}, \\ \mathrm{Recall}(\theta) &= \frac{\mathrm{TP}(\theta)}{P}, \end{aligned} \tag{19}$$

where $\theta \in [0, 1]$ is a prescribed cut-off threshold used for excluding links with small posterior presence probabilities, $P(Q)$ is the number of positives (negatives) in the gold standard, i.e., the number of present (absent) links in the real underlying network $\mathcal{G}$, $\hat{L}(\theta) = \mathrm{TP}(\theta) + \mathrm{FP}(\theta)$ is the link number of the estimated network after thresholding, and $\mathrm{TP}(\theta)$ $[\mathrm{FP}(\theta)]$ is the number of true positives (false positives) in the remaining links. More precisely,

$$\begin{aligned} \mathrm{TP}(\theta) &= \#\Big\{(u,v) \in \mathcal{G} \Big| \frac{1}{M} \textstyle\sum_{\hat{\mathcal{G}} \in \hat{\mathbf{G}}} \mathbb{1}_{[(u,v)\in\hat{\mathcal{G}}]} \geq \theta\Big\}, \\ \mathrm{FP}(\theta) &= \#\Big\{(u,v) \notin \mathcal{G} \Big| \frac{1}{M} \textstyle\sum_{\hat{\mathcal{G}} \in \hat{\mathbf{G}}} \mathbb{1}_{[(u,v)\in\hat{\mathcal{G}}]} \geq \theta\Big\}, \end{aligned} \tag{20}$$

where the symbol # denotes the set cardinality, and $\hat{\mathbf{G}}$ are the samples of time-aggregated graphs drawn from the Gibbs sampler. Note that $\mathrm{Recall}(\theta)$ (also called sensitivity) is equivalent to $\mathrm{TPR}(\theta)$.

Due to the sparsity nature of empirical complex networks (namely, $P \ll Q$), the inferential problem can be thought of as a highly skewed classification task. Therefore, another measurement index, called break-even point (BEP) [39], is preferably used for controlling Type I and Type II classification errors. The BEP strikes the best balance between precision and recall on positive links at a break-even cut-off θ^* such that $\mathrm{Precision}(\theta^*) = \mathrm{Recall}(\theta^*)$. Note that $P = \mathrm{TP}(\theta) + \mathrm{FN}(\theta)$, where $\mathrm{FN}(\theta) = \#\big\{(u,v) \in \mathcal{G} | q_{uv} < \theta\big\}$ is the number of false negative in the excluded links after thresholding. Thus the BEP attained at the optimal cut-off θ^* corresponds to satisfying $\mathrm{FP}(\theta^*) = \mathrm{FN}(\theta^*)$, that is, the number of false-positive links is equal to the number of false-negative links, as illustrated in Fig. 2 in the main text. Note that the actual values of the BEP depend on underlying topologies, thus raising the question as to how to choose the optimal cut-off θ^*

for thresholding in practice. Here we consider the predicted ROC and PR curves according to

$$\begin{aligned}
\widehat{\mathrm{TP}}(\hat{\theta}) &= \sum_{(u,v):\hat{q}_{uv}\geq\hat{\theta}} \hat{q}_{uv}, \quad \widehat{\mathrm{FP}}(\hat{\theta}) = \sum_{(u,v):\hat{q}_{uv}\geq\hat{\theta}} (1-\hat{q}_{uv}),\\
\widehat{\mathrm{FN}}(\hat{\theta}) &= \sum_{(u,v):\hat{q}_{uv}<\hat{\theta}} \hat{q}_{uv}, \quad \widehat{\mathrm{TN}}(\hat{\theta}) = \sum_{(u,v):\hat{q}_{uv}<\hat{\theta}} (1-\hat{q}_{uv}),\\
\widehat{P} &= \widehat{\mathrm{TP}}(\hat{\theta}) + \widehat{\mathrm{FN}}(\hat{\theta}), \quad \widehat{Q} = \widehat{\mathrm{FP}}(\hat{\theta}) + \widehat{\mathrm{TN}}(\hat{\theta}),
\end{aligned} \tag{21}$$

where $\hat{q}_{uv} = M^{-1}\sum_{\hat{\mathcal{G}}\in\hat{\mathbf{G}}} \mathbb{1}_{[(u,v)\in\hat{\mathcal{G}}]}$ is the posterior presence probability of link (u,v) in the estimated network. This allows an unsupervised selection procedure for the cut-off $\hat{\theta}^*$ such that $\widehat{\mathrm{FP}}(\hat{\theta}^*) = \widehat{\mathrm{FN}}(\hat{\theta}^*)$, which corresponds to the BEP of the predicted PR curves.

Supplementary Note 5: Asymptotic consistency proof for the iterative WTD estimator

We denote the converged estimate of underlying WTDs by $\hat{\boldsymbol{\rho}} = [\hat{\rho}_{uv}(\tau)]_{(u,v)\in\mathcal{G}}$, which satisfies the following self-consistent relation:

$$\hat{\rho}_{uv}(\tau) = \frac{1}{C}\sum_{i=1}^{C}\left\{\beta_{uv}(\mathcal{D}^i,\mathcal{G},\hat{\boldsymbol{\rho}})K_h(\tau-d_{uv}^i) + \left[1-\beta_{uv}(\mathcal{D}^i,\mathcal{G},\hat{\boldsymbol{\rho}})\right]\frac{\hat{\rho}_{uv}(\tau)\Theta(\tau-d_{uv}^i)}{\hat{\Phi}_{uv}(d_{uv}^i)}\otimes K_h(\tau)\right\}, \tag{22}$$

We next prove $\hat{\boldsymbol{\rho}} = \boldsymbol{\rho}$ in the asymptotic limit as sample size $C\to\infty$ and kernel bandwidth $h\to 0$.

Asymptotic consistency proof. If the underlying network $\mathcal{G}$ consists of only two nodes, we are already done [in which case our estimator reduces to standard kernel density estimation, $\hat{\rho}_{uv}(\tau) = C^{-1}\sum_{i=1}^{C}K_h(\tau-d_{uv}^i)$]. Then we proceed by induction on the network size. Let $v^i = \arg\max_{v\in\mathcal{V}} t_v^i$ be the latest informed node in the cascade $\mathcal{D}^i$, D' be the reduced DATs of D obtained by deleting v^i from each cascade $\mathcal{D}^i \in D$, and $\hat{\boldsymbol{\rho}}'$ be the estimated WTDs from D'. By our induction assumption, we have $\hat{\boldsymbol{\rho}}' = \boldsymbol{\rho}$. Therefore we only need to show that the estimated $\hat{\boldsymbol{\rho}}''$ from the appended data of DATs (i.e., the subsequent diffusion process towards node v^i posterior to $\mathcal{D}^{i'} \in D'$) also converges to $\boldsymbol{\rho}$. For brevity, we use the same notation $\hat{\boldsymbol{\rho}}$ in place of $\hat{\boldsymbol{\rho}}''$, if no confusion arises.

We next prove $\hat{\boldsymbol{\rho}} = \boldsymbol{\rho}$. Suppose that v is a last node to be informed during a corresponding cascade. Let u_i and t_i be node v's incoming neighbours and their DATs, for $i = 1,\ldots,\#\mathcal{I}_v$, where $\#\mathcal{I}_v$ is the in-degree of node v. To lighten the notation, we denote link (u_i, v) by i. Then the complementary cumulative probability function of node v's DAT is $S(t) = \prod_i \Phi_i(t-t_i)$, and hence its PDF reads $f(t) = -dS(t)/dt = \sum_i \rho_i(t-t_i)S_{-i}(t)$, where $S_{-i}(t) = \prod_{j\neq i}\Phi_j(t-t_j)$. Thus the probability of i acting as a diffusion branch with waiting time τ is given by

$$f_i(\tau) = \rho_i(\tau)S_{-i}(t_i+\tau). \tag{23}$$

Next we calculate the probability [denoted by $f_{i\prec j}(\tau)$] that i acts as a chord with waiting time τ, while another $j(\neq i)$ acts as the diffusion branch. This can be implemented in two stages: first, u_j informs v prior to u_i as well as any other neighbours at some instant $t < t_i+\tau$, the probability of which is $f_j(t-t_j)$ according to equation (23); and then u_i produces a waiting time τ from a truncated WTD such that $\tau_i > t - t_i$, because conditioning on the previous stage, the instant u_i informs v must be later than t. Therefore we have

$$f_{i\prec j}(\tau) = \int_{-\infty}^{t_i+\tau}\frac{\rho_i(\tau)}{\Phi_i(t-t_i)}f_j(t-t_j)dt = \rho_i(\tau)\int_{-\infty}^{\tau}\frac{f_j(\tau'-d_{ij})d\tau'}{\Phi_i(\tau')}, \tag{24}$$

where $d_{ij} = t_j - t_i$ is the TDOA between u_i and u_j. Here we adopted the conventions $\rho_i(\tau) = 0, \Phi_i(\tau) = 1$, $\tau < 0$.

One can readily verify the following equality

$$f_i(\tau) + \sum_{j\neq i} f_{i\prec j}(\tau) = \rho_i(\tau). \tag{25}$$

It is equivalent to checking that

$$A(\tau) = S_{-i}(t_i + \tau) + \sum_{j \neq i} \int_{-\infty}^{\tau} \frac{f_j(\tau' - d_{ij}) d\tau'}{\Phi_i(\tau')} \equiv 1, \tag{26}$$

which is satisfied because $dA(\tau)/d\tau = 0$ and $A(-\infty) = 1$.

On the other hand, with branching coefficients [equation (14)] $\beta_i(t_v) = \lambda_i(t_v - t_i)/\sum_j \lambda_j(t_v - t_j) = f_i(t_v - t_i)/f(t_v)$, we can rewrite $f_i(\tau)$ as $f_i(\tau) = \beta_i(\tau + t_i) f(\tau + t_i) = \int_{-\infty}^{\infty} \beta_i(t) f(t) \delta(t - t_i - \tau) dt$. (Here we emphasize the dependence of β_i on t_v^a, and suppress the other arguments, $\{t_u^a\}_{u \in \mathcal{I}_v}$, $\mathcal{I}_v$ and $\boldsymbol{\rho}$.) With the observed DATs $\{t_v^a\}$ of node v from corresponding diffusion cascades D_v, plugging the empirical PDF, $\bar{f}(t) = C_v^{-1} \sum_a \delta(t - t_v^a)$, yields

$$\bar{f}_i(\tau) = C_v^{-1} \sum_{a=1}^{C_v} \beta_i(t_v^a) \delta(\tau - d_{iv}^a), \tag{27}$$

where $d_{iv}^a = t_v^a - t_i^a$ is the TDOA between u_i and v in cascade $\mathcal{D}_v^a$, and $C_v = \#\{\mathcal{D}^a \in D | \arg\max_{u \in \mathcal{V}} t_u^a = v\}$. Without loss of generality, we assume that the diffusion cascades such that share the same eventually informed node v are relabelled 1 through C_v. Clearly, $\sum_{v \in \mathcal{V}} C_v = C$.

Then inserting into equation (24) we obtain

$$\bar{f}_{i \prec j}(\tau) = C_v^{-1} \sum_{a=1}^{C_v} \beta_j(t_v^a) \frac{\rho_i(\tau) \Theta(\tau - d_{iv}^a)}{\Phi_i(d_{iv}^a)}. \tag{28}$$

In the asymptotic limit $C_v \to \infty$, the Glivenko-Cantelli Theorem implies $\bar{f}_i(\tau) \to f_i(\tau)$, $\bar{f}_{i \prec j}(\tau) \to f_{i \prec j}(\tau)$ a.s., and thus the updating rule [equation (22)] can be simply reexpressed as

$$\hat{\rho}_i^{k+1}(\tau) = \hat{f}_i^k(\tau) + \sum_{j \neq i} \hat{f}_{i \prec j}^k(\tau), \tag{29}$$

with $\hat{f}_i^k(\tau)[\hat{f}_{i \prec j}^k(\tau)]$ obtained by replacing $\rho_i(\tau)$ by the current WTD estimate $\hat{\rho}_i^k(\tau)$. We note here that $\hat{f}_i^k(\tau) = \hat{\beta}_i^k(\tau + t_i) f(\tau + t_i)$, where $\hat{\beta}_i^k(t)$ is the estimated branching coefficients from $\hat{\rho}_i^k(\tau)$, and $f(t)$ is true due to the infinitely many empirical observations $\{t^a\}$ in the asymptotic limit. Since integration of equation (24) by parts gives

$$\int_x^{\infty} \hat{f}_{i \prec j}^k(\tau) d\tau = -\int_x^{\infty} d\hat{\Phi}_i^k(\tau) \int_{-\infty}^{\tau} \frac{\hat{f}_j^k(\tau' - d_{ij}) d\tau'}{\hat{\Phi}_i^k(\tau')} = \hat{S}_j^k(x - d_{ij}) + \hat{\Phi}_i^k(x) \int_{-\infty}^{x} \frac{\hat{f}_j^k(\tau' - d_{ij}) d\tau'}{\hat{\Phi}_i^k(\tau')}, \tag{30}$$

where $\hat{S}_j(\tau)$ is the survival function of $\hat{f}_j(\tau)$.

Thus integrating both sides of equation (29) from τ to ∞ yields

$$\hat{\Phi}_i^{k+1}(\tau) = \sum_j \hat{S}_j^k(\tau - d_{ij}) + \hat{\Phi}_i^k(\tau) \sum_{j \neq i} \int_{-\infty}^{\tau} \frac{\hat{f}_j^k(\tau' - d_{ij}) d\tau'}{\hat{\Phi}_i^k(\tau')}. \tag{31}$$

Recalling $\hat{f}_i^k(\tau) = \hat{\beta}_i^k(\tau + t_i) f(\tau + t_i)$, from the normalisation condition for the branching coefficients [equation (14)] we obtain $\sum_j \hat{f}_j^k(\tau - d_{ij}) = f(\tau + t_i)$ regardless of any deviation in the estimation.

Taking limit as $k \to \infty$ gives

$$\hat{\Phi}_i(\tau) = S(\tau + t_i) + \hat{\Phi}_i(\tau) \int_{-\infty}^{\tau} \frac{f(\tau' + t_i)[1 - \hat{\beta}_i(\tau' + t_i)]}{\hat{\Phi}_i(\tau')} d\tau', \tag{32}$$

whence we have $\hat{\Phi}_i(\tau)\hat{\Psi}_i(\tau) = S(\tau + t_i)$, letting

$$\hat{\Psi}_i(\tau) = 1 - \int_{-\infty}^{\tau} \frac{f(\tau' + t_i)[1 - \hat{\beta}_i(\tau' + t_i)]}{\hat{\Phi}_i(\tau')} d\tau', \tag{33}$$

which can be interpreted as the cavity probability of v not being informed prior to $\tau + t_i$, given that i is a chord and removed from the local configuration $\mathcal{I}_v$ as a cavity link.

Thus the self-consistently estimated survival functions $\hat{\Phi}_i(\tau)$ together with $\hat{\Psi}_i(\tau)$ constitute a solution to the following set of differential equations:

$$\begin{cases} \dfrac{d}{d\tau}\hat{\Phi}_i = -\dfrac{f(\tau + t_i)\hat{\beta}_i(\tau + t_i)}{\hat{\Psi}_i(\tau)}, & \text{(34a)} \\[2ex] \dfrac{d}{d\tau}\hat{\Psi}_i = -\dfrac{f(\tau + t_i)[1 - \hat{\beta}_i(\tau + t_i)]}{\hat{\Phi}_i(\tau)}, & \text{(34b)} \end{cases}$$

with initial conditions $\hat{\Phi}_i(-\infty) = \hat{\Psi}_i(-\infty) = 1$.

Since true WTD $\rho_i(\tau)$ also satisfies the self-consistent condition [equation (25)], it is therefore a solution to equation (34) with exactly the same initial conditions. Finally, from standard theorems on the uniqueness of solutions to differential equations it follows $\hat{\rho}_i(\tau) = \rho_i(\tau)$, which completes the proof.

Remark.—As a final remark of this note, recalling the hypothetical case of observable diffusion trees (see the remark at the end of Supplementary Note 2), the proposed WTD estimator [equation (22)] reduces to

$$\hat{\rho}_{uv}(\tau) = \frac{1}{C} \sum_{i=1}^{C} \left\{ \chi_{uv}^i K_h(\tau - d_{uv}^i) + (1 - \chi_{uv}^i) \frac{\hat{\rho}_{uv}(\tau)\Theta(\tau - d_{uv}^i)}{\hat{\Phi}_{uv}(d_{uv}^i)} \otimes K_h(\tau) \right\}, \tag{35}$$

where $\chi_{uv}^i = \mathbb{1}_{[(u,v)\in\mathcal{T}^i]}$ are the observed binary indicators of diffusion branches for cascade $\mathcal{D}^i$ on $\mathcal{N}_{\mathrm{s}}$. Equation (35) closely resembles equation (22) with χ_{uv}^i replaced by its expectation $\beta_{uv}(\mathcal{D}^i, \mathcal{G}, \hat{\boldsymbol{\rho}}) = \mathbb{E}\left[\chi_{uv}^i \middle| \mathcal{G}, \hat{\boldsymbol{\rho}}\right]$ when the data $\boldsymbol{\chi}$ are missing, as we have already seen [equation (9)]. In addition, combining Eqs. (22) and (35) corresponds a hybrid case of partially observed $\boldsymbol{\chi}$. In other words, information can be observably transmitted among a fraction of the population during the diffusion process, depending on the act of an information recipient, whether revealing or concealing her sender.

Supplementary Note 6: Selection of first-guess WTD and smoothing kernel bandwidth

Selection of first-guess WTD.—In spite that the iteratively estimated WTD $\hat{\rho}(D, \mathcal{G})$ are irrespective of initial conditions, an improperly selected initial guess $\hat{\rho}^0$ for WTDs may cause extremely slow final convergence in practice. To demonstrate how the initial conditions affect the convergence rate of our iterative procedure, we consider two typical cases of erroneously guessed $\hat{\rho}^0$: first, where $\hat{\rho}^0$ underestimates real waiting times [Supplementary Figure 4(a)], and second, where $\hat{\rho}^0$ overestimates real waiting times [Supplementary Figure 4(b)]. We find that in general the number of iterations it needs for compensating the overestimated WTDs is overwhelming compared to that for the underestimated case, in part because of the "Redistribute-to-the-Right" imputation method we adopted to handle censored waiting times of diffusion chords in our inference procedure. Therefore, an initial $\hat{\rho}^0$ is preferably chosen as a right-skewed distribution relative to the real WTD to obtain a better rate of convergence. Hence we select $\hat{\rho}^0$ as exponential distribution

$$\hat{\rho}^0(\tau) = \frac{1}{\mu_0} \exp(-x/\mu_0), \tag{36}$$

with mean μ_0 equal to the average TDOA of observed first diffusion branches outgoing from the source node, denoted by

$$\mu_0 = \Big\langle \min_{v \in \mathcal{V} \setminus s^*} t_v - t_{s^*} \Big\rangle_D, \tag{37}$$

where the angular brackets denote the average over the observed diffusion cascades D. Note that the TDOAs of such first diffusion branches correspond to uncensored observations of waiting times, which is obviously underestimated due to their extreme value properties.

Selection of smoothing kernel bandwidth.—We have showed the asymptotic consistency of the proposed WTD estimator, whereas in practice only finite data are available for inference. Therefore we adopt a smoothed version of the self-consistent estimator, where the Gaussian kernel $K_h(\tau) = \frac{1}{\sqrt{2\pi h^2}} \exp(-\tau^2/2h^2)$ is used as a regulariser in the task of density estimation, and the bandwidth h determines the spread of the kernel, which affect the degree of approximation between the reconstructed and real WTDs, as shown in Supplementary Figure 5.

However, it is difficult to optimally balance the overfitting and underfitting risks, particularly when neither the time-aggregated network nor the actual WTD can be observed. Given observed or numerically synthesised DAT cascades on temporal networks, one can repeat the inference procedure over a grid of kernel bandwidths to determine the overall best-fitting value of h^* in the sense of minimizing an objective function consisting of a goodness-of-fit term and a regularisation term as follows:

$$h^* = \arg\min_h \left\{ \|\rho, \hat{\rho}_h\|_{\mathrm{KS}} + \lambda \int_0^\infty \|\hat{\rho}_h^{(k)}(\tau)\|_p d\tau \right\},$$

where $\|\rho, \hat{\rho}_h\|_{\mathrm{KS}}$ denotes the Kolmogorov-Smirnov distance between the real and reconstructed WTD, $\|\cdot\|_p$ is the vector p-norm, and $\hat{\rho}_h^{(k)}$ represents the k-times derivative of the WTD estimate using kernel bandwidth h. Here roughness penalties are employed to regularize the calculation, λ being a nonnegative smoothing parameter controlling the size of the roughness penalty.

Clearly, the best-fitting kernel bandwidth h^* heavily relies on the real WTD ρ and the smoothing parameter λ. By numerically testing a wide range values of h, we find that the procedure for network inference is insensitive to the bandwidth choice. Supplementary Figure 5(j) shows the critical relative sample sizes that ensure at least 0.95 BEP for inference of the benchmark STNs, implying that kernel bandwidths have no qualitative effect on the reconstruction accuracy. Unless otherwise stated, we hereafter adopt an intermediate kernel bandwidth $h = 0.05$ in our numerical simulations.

To achieve unsupervised estimation, we provide the following practical recommendations for choice of kernel bandwidths, which can be used as a crude rule of the thumb:

$$h \sim 0.01 \times \Phi^{-1}(\alpha), \quad \alpha = \begin{cases} 0 & \text{for bounded WTDs,} \\ 0.01 & \text{for diffusive WTDs,} \end{cases}$$

where the inverse survival function $\Phi^{-1}(\alpha)$ represents the upper bound of $(1-\alpha)$-support of the WTD.

Supplementary Note 7: Detailed algorithm implementation

Generative model.—We first outline the generative algorithm used as the forward model to synthesize diffusion cascades, assuming, for brevity, that information propagates through a homogeneous population with identical WTD $\rho_{uv}(\tau) \equiv \rho(\tau)$, for each link $(u, v) \in \mathcal{G}$. Note that the described algorithms can be naturally extended to non-identically distributed WTDs. We represent $\rho(\tau)$ in computer programs by the following discretization: divide the support of the WTD into equally spaced bins of width δt, and for each bin $(i\delta t, (i+1)\delta t]$, place a probability mass $\rho[i+1]\delta t = \int_0^{\delta t} \rho(i\delta t + \tau')d\tau'$ at the right endpoint of the interval. Here, we use the shorthand $\rho[i]$ to denote $\rho[i\delta t]$ for notational simplicity. Thus the binned version of the survival and hazard functions of $\rho(\tau)$ is accordingly written as

$$\Phi[i] = 1 - \sum_{j<i} \rho[i], \qquad \lambda[i] = \rho[i]/\Phi[i], \tag{38}$$

with the conventions $\Phi[i] = 1$ and $\rho[i] = \lambda[i] = 0$, $i < 0$. The effect of nonzero discretisation size (bin width δt) on our inferential algorithm is further discussed in Supplementary Note 10. Specifically, the generative model first produces a randomly weighted network with τ_{uv} corresponding to the realized waiting time on each link $(u, v) \in \mathcal{G}$ during the diffusion cascade. Here, we use the inverse-transform method [41] to extract τ_{uv} from WTD $\rho[\cdot]$, and then employs the single-source Dijkstra algorithm [42] to determine the cascade of DATs $\mathcal{D}$ as shortest path lengths from the source node s^* to every other nodes of $\mathcal{G}$, as Algorithm 3 shows.

Coordinate-ascent iterative inference for STN model.—As outlined in Algorithm 4, the overall procedure of algorithm iteratively alternates between (1) Markov chain Monte Carlo sampling the time-aggregated topology space of network $\mathcal{G}$, and (2) self-consistently estimating the dyad-specific WTD ρ underlying the temporal interaction sequences. Next we specify the two steps, topology inference and density estimation, separately.

Time-aggregated network inference.—We apply a Markov chain Monte Carlo (MCMC) method [41] to explore the configuration space of static network topology. To ensure the stationarity of Markov chains, a burn-in period of 10 is used in our algorithm. The maximum lag is also set to 10 to enhance the independence of samples, and the number $M = 200$ of network configurations are drawn from the sampling procedure. In general, the major computational time complexity is due to the calculation of the marginal gain $\Delta_{uv}\ell(D|\hat{\mathcal{G}}, \rho)$ of flipping any link (u, v) in the current configuration $\hat{\mathcal{G}}$. Under the null model assumption of independent WTDs, this can be done efficiently. Noting that $\Delta_{uv}\ell(D|\hat{\mathcal{G}}, \rho) = \sum_{i=1}^{C}\{\log[1 \pm \beta_{uv}(\mathcal{D}^i, \hat{\mathcal{G}}, \rho)] \pm \log \Phi_{uv}(d_{uv}^i)\}$, we only need to compute the branching coefficient $\beta_{uv}(\mathcal{D}^i, \hat{\mathcal{G}}, \rho)$. Towards this end, we record and update the diagonal matrix entries $[\Lambda_v(\mathcal{D}^i|\hat{\mathcal{G}}, \rho)]_{v\in\mathcal{V}}$ of the weighted in-degree Laplacians L^{in} of the current configuration $\hat{\mathcal{G}}$ with respect to the observed DAT cascades $\mathcal{D}^i \in D$, as displayed in Algorithm 5. Also, our topology inference algorithm is still valid for undirected networks, in which case each undirected edge is regarded as two directed links of opposite directions, and, accordingly, calculation of marginal gains $[(\Delta_{uv} + \Delta_{vu})\ell(D|\hat{\mathcal{G}}, \rho)]$, link flipping operations $[\hat{\mathcal{G}} \leftarrow \hat{\mathcal{G}} \cup \{(u, v), (v, u)\}, \hat{\mathcal{G}} \setminus \{(u, v), (v, u)\}]$, as well as updates of cumulative hazard rates $[\Lambda_v(\mathcal{D}^i|\hat{\mathcal{G}}, \rho), \Lambda_u(\mathcal{D}^i|\hat{\mathcal{G}}, \rho)]$ are proceeded for both links (u, v) and (v, u) simultaneously.

Density estimation for WTD.—The detailed implementation of the density estimation step $\hat{\rho}(D, \hat{\mathcal{G}})$ in the main text is described in Algorithm 6. To initialize an iterative scheme for self-consistently estimating the

underlying WTD, the procedure first determines the *a priori* guess $\hat{\rho}_0$ and the smoothing kernel bandwidth h through the observed diffusion cascades (see Methods for more details on the automatic selection procedures), and updates the WTD estimate $\hat{\rho}^k(D, \hat{\mathcal{G}})$ according to self-consistency conditions until convergence is reached.

Bootstrap confidence analysis.— To measure the approximation level of the reconstructed STN model, we use bootstrap confidence analysis—originally proposed by Efron [43] and standardised as a computer-intensive resampling procedure for hypothesis tests and confidence interval estimation [44]—for calculating the confidence bands of estimated WTDs. More specifically, we adopted the parametric bootstrap method, where both G and ρ are regarded as generalised "parameters" of the data generative process, and the bootstrap resamples of diffusion cascades can thus be generated using the corresponding STN tuple $\hat{N}_s = (\hat{G}, \hat{\rho})$ estimated from the original diffusion data. A pseudo-code implementation for the bootstrap confidence analysis is provided in Algorithm 7, and the benchmark test results are shown in Supplementary Figure 7.

Algorithm 1: Random Number Generator with Inverse-Transform Method

Input: $\Phi[\cdot]$ ▷ Discretised survival function of the WTD
Output: τ ▷ Random (binned) waiting time drawn from the input WTD
$r \leftarrow uniform(0, 1)$; ▷ Draw a random number uniformly distributed within $[0, 1]$
$\tau_{\text{lower}} \leftarrow 0$; $\tau_{\text{upper}} \leftarrow l_\tau$; ▷ l_τ is the upper bound of the support of $\Phi[\cdot]$
while $\tau_{\text{upper}} - \tau_{\text{lower}} > 1$ **do** ▷ Binary search used to locate the bin into which the random number r falls
  $\tau = \lfloor (\tau_{\text{lower}} + \tau_{\text{upper}})/2 \rfloor$;
  if $r < \Phi[\tau]$ **then** $\tau_{\text{lower}} \leftarrow \tau$ **else** $\tau_{\text{upper}} \leftarrow \tau$;
return τ

Algorithm 2: Single-source Dijkstra Shortest Path Algorithm

Input: $\{\tau_{uv}\}_{(u,v)\in\mathcal{G}}$, s^* ▷ Link-weighted G representing the realized waiting times, as well as source node s^*
Output: $[t_v]_{v\in\mathcal{G}}$ ▷ The synthesized cascade of DAT $\mathcal{D}$ as shortest path lengths from s^* to respective nodes
$R = \{s^*\}$; $t_{s^*} = 0$; ▷ R is the set of *informed* nodes, and $t_{s^*} = 0$ is the initial time of the diffusion cascade
while $R \neq \mathcal{V}$ **do** ▷ Whenever there is any currently *ignorant* node in the network
  $d_{v^*} \leftarrow \min_{u\in R, v\in\mathcal{V}\backslash R}\{t_u + \tau_{uv}\}$; ▷ Determine the next *informed* node v^* and its shortest path length
  $R \leftarrow R \cup \{v^*\}$; $t_{v^*} \leftarrow d_{v^*}$; ▷ Add v^* to the *informed* node set and record its shortest path length from s^*
return $[t_v]_{v\in\mathcal{G}}$

Algorithm 3: Generative Model of Single Diffusion Cascades

Input: $\mathcal{G}$, $\rho[\cdot]$, s^* ▷ STN model and information source
Output: $\mathcal{D}$ ▷ Single diffusion cascade of DATs
$\Phi[\tau] \leftarrow 1 - \sum_{\tau'<\tau} \rho[\tau'], \tau \in \{1, \dots, l_\tau\}$; ▷ Calculate the survival function of the WTD using equation (38)
foreach $(u, v) \in \mathcal{G}$ **do**
  $\tau_{uv} \leftarrow inverse\_transform(\Phi[\cdot])$; ▷ Generate random waiting times with the Inverse-Transform method
$\mathcal{D} \leftarrow dijkstra(\{\tau_{uv}\}, s^*)$; ▷ Determine all DATs t_v as FTP lengths from s^* to v using the Dijkstra method
return $\mathcal{D}$

Algorithm 4: Iterative Inference Procedure for STN Model

Input: D ▷ Sample of observed diffusion cascades on the STN

$\hat{\rho} \leftarrow \hat{\rho}^0(D)$ ▷ Initialize the iteration with a data-driven guess (see Method and Line 1 of Algorithm 6)

repeat

  $\hat{\rho}' \leftarrow \hat{\rho}$ ▷ Record the current WTD estimate

  $\hat{\mathbf{G}} \leftarrow \textit{topology\_inference\_step}(D, \hat{\rho})$ ▷ Update the current topology inference $\hat{\mathbf{G}}$ using Algorithm 5

  $\hat{\rho} \leftarrow \textit{density\_estimation\_step}(D, \hat{\mathbf{G}})$ ▷ Update the current density estimation $\hat{\rho}$ using Algorithm 6

until $\|\hat{\rho}, \hat{\rho}'\|_{\mathrm{KS}} < \epsilon$; ▷ Convergence is judged by the Kolmogorov-Smirnov divergence between $\hat{\rho}$ and $\hat{\rho}'$

$\hat{\theta}^* \leftarrow \textit{predicted\_BEP}(\hat{\mathbf{G}})$ ▷ Calculate the predicted BEP [see equation (21) in Supplementary Note 4]

$\hat{\mathcal{G}} \leftarrow \textit{thresholding}(\hat{\mathbf{G}}, \hat{\theta}^*)$ ▷ A single topology estimate after thresholding, $\hat{\mathcal{G}} = \left\{(u,v) \in \mathcal{V}^2 : \hat{q}_{uv} \geq \hat{\theta}^*\right\}$

Output: $\hat{\mathcal{N}}_{\mathrm{s}} = (\hat{\mathcal{G}}, \hat{\rho})$ ▷ Converged estimates of the STN tuple

Algorithm 5: MCMC Algorithm with Gibbs Sampling

Input: $\rho[\cdot]$, D ▷ Binned WTD and sample of diffusion cascades

  burn_in, *max_lag*, M ▷ Burn-in period, maximum lag and sample size used in the MCMC sampling

Output: $\hat{\mathbf{G}}$ ▷ MCMC Samples drawn from the configuration space of network $\mathcal{G}$ according to $L(D|\mathcal{G}, \rho)$

$\hat{\Phi}[\tau] \leftarrow 1 - \sum_{\tau' < \tau} \rho[\tau']$, $\hat{\lambda}[\tau] = \rho[\tau]/\Phi[\tau]$, $\tau \in \{1, \ldots, l_\tau\}$; ▷ Calculate the survival and hazard functions

$\hat{\mathcal{G}} \leftarrow$ *empty graph*; ▷ The initial network configuration $\hat{\mathcal{G}}^0$ is assumed for simplicity to be an empty graph

$[\Lambda_v^i]_{v \in \mathcal{V}} \leftarrow \varepsilon$, $i \in \{1, \ldots, C\}$; ▷ Diagonal entries $\Lambda_v^i \triangleq \Lambda_v(\mathcal{D}^i|\hat{\mathcal{G}}, \rho)$ of Laplacian L^{in} initialized as $\varepsilon = 10^{-5}$

$\hat{\mathbf{G}} \leftarrow \varnothing$; *iteration* $\leftarrow 0$; *lag* $\leftarrow 0$;

while $\#\hat{\mathbf{G}} < M$ **do** ▷ Iterate until the number of sampled configurations reaches the predefined size M

  iteration $\leftarrow$ *iteration* $+ 1$;

  foreach (u, v) **do** ▷ The Gibbs sampler alternately considers every proposal of flipping link (u, v)

    if $(u, v) \in \hat{\mathcal{G}}$ **then** ▷ For present links

      $\Delta_{uv}\ell(D|\hat{\mathcal{G}}, \rho) \leftarrow \sum_{i=1}^{C} \left\{\log\left(1 - \lambda[d_{uv}^i]/\Lambda_v^i\right) - \log \Phi[d_{uv}^i]\right\}$; ▷ Marginal gain of removing (u, v)

      $p_{uv} \leftarrow \left[1 + \exp\left(-\Delta_{uv}\ell(D|\mathcal{G}, \rho)\right)\right]^{-1}$; $r \leftarrow \textit{uniform}(0, 1)$;

      if $r < p_{uv}$ **then** ▷ Accept the proposal with probability p_{uv}

        $\hat{\mathcal{G}} \leftarrow \hat{\mathcal{G}} \setminus (u, v)$; ▷ Remove link (u, v) from the current configuration $\hat{\mathcal{G}}$

        $\Lambda_v^i \leftarrow \left(\Lambda_v^i - \lambda[d_{uv}^i]\right) \big/ \left(1 - \lambda[d_{uv}^i]\delta t\right)$, $i \in \{1, \ldots, C\}$; ▷ Update $\Lambda_v(\mathcal{D}^i|\hat{\mathcal{G}}, \rho)$ for each $\mathcal{D}^i \in D$

    else ▷ For absent links

      $\Delta_{uv}\ell(D|\hat{\mathcal{G}}, \rho) \leftarrow \sum_{i=1}^{C} \left\{\log\left(1 + \lambda[d_{uv}^i]/\Lambda_v^i\right) + \log \Phi[d_{uv}^i]\right\}$; ▷ Marginal gain of adding (u, v)

      $p_{uv} \leftarrow \left[1 + \exp\left(-\Delta_{uv}\ell(D|\mathcal{G}, \rho)\right)\right]^{-1}$; $r \leftarrow \textit{uniform}(0, 1)$;

      if $r < p_{uv}$ **then** ▷ Accept the proposal with probability p_{uv}

        $\hat{\mathcal{G}} \leftarrow \hat{\mathcal{G}} \cup (u, v)$; ▷ Add link (u, v) to the current configuration $\hat{\mathcal{G}}$

        $\Lambda_v^i \leftarrow \left(1 - \lambda[d_{uv}^i]\delta t\right) * \Lambda_v^i + \lambda[d_{uv}^i]$, $i \in \{1, \ldots, C\}$; ▷ Update $\Lambda_v(\mathcal{D}^i|\hat{\mathcal{G}}, \rho)$ for each $\mathcal{D}^i \in D$

  if *iteration* $>$ *burn_in* **then** ▷ Discard the burn-in samples to ensure stationarity of the Markov chain

    lag $\leftarrow$ *lag* $+ 1$;

  if *lag* $=$ *max_lag* **then** ▷ Graph $\hat{\mathcal{G}}$ is drawn every *max_lag* iterations to ensure independence of samples

    $\hat{\mathbf{G}} \leftarrow \hat{\mathbf{G}} \cup \hat{\mathcal{G}}$; *lag* $\leftarrow 0$;

return $\hat{\mathbf{G}}$

Algorithm 6: Density Estimation for WTD

Input: $\hat{\mathbf{G}}$, D, l_τ ▷ Sampled graph configurations, observed DAT cascades, and support range of WTD

Output: $\hat{\rho}[\cdot]$ ▷ Estimated WTD

$\mu_0 \leftarrow \left\langle \min_{v\in\mathcal{V}\setminus s^*} t_v \right\rangle_D$; $\hat{\rho}[\cdot] \leftarrow \mathrm{Exp}[\mu_0]$; ▷ Initialize the *a priori* WTD as (binned) exponential of mean μ_0

$h \leftarrow 0.01 * l_\tau$; $K_h[\cdot] \leftarrow {K_h}^{Gauss}(\cdot)$; ▷ Select the discretised kernel smoother as Gaussian with bandwidth h

repeat

  $\hat{\Phi}[\tau] \leftarrow 1 - \sum_{\tau'<\tau} \rho[\tau']$, $\hat{\lambda}[\tau] = \rho[\tau]/\Phi[\tau]$, $\tau \in \{1, \dots, l_\tau\}$; ▷ Calculate survival and hazard functions

  $tdoa[\tau] \leftarrow 0$, $beta[\tau] \leftarrow 0$, $\tau \in \{1, \dots, l_\tau\}$; ▷ Statistics of branching coefficients by different TDOAs

  foreach $\hat{\mathcal{G}} \in \hat{\mathbf{G}}$ **do**

    foreach $\mathcal{D}^i \in D$ **do**

      foreach $(u, v) \in \hat{\mathcal{G}}$ **do**

        if $d^i_{uv} \in \{1, \dots, l_\tau\}$ **then**

          $tdoa[d^i_{uv}] \leftarrow tdoa[d^i_{uv}] + 1$; ▷ Count different TDOAs from respective network links

          $beta[d^i_{uv}] \leftarrow beta[d^i_{uv}] + \hat{\lambda}(d^i_{uv}) / \sum_{w\in\hat{\mathcal{G}}} \hat{\lambda}(d^i_{wv})$; ▷ Count branching coefficients

  $\hat{\rho}^\dagger[\tau] \leftarrow 0$, $\tau \in \{1, \dots, l_\tau\}$;

  for $\tau \in \{1, \dots, l_\tau\}$ **do** ▷ Calculate the next WTD estimate

    if $tdoa[\tau] > 0$ **then**

      $\hat{\rho}^\dagger[\tau] \leftarrow \hat{\rho}^\dagger[\tau] + beta[\tau]$; ▷ For observed waiting times on diffusion branches

      for $\tau' \in \{\tau + 1, \dots, l_\tau\}$ **do**

        $\hat{\rho}^\dagger[\tau'] \leftarrow \hat{\rho}^\dagger[\tau'] + (tdoa[\tau] - beta[\tau]) * \hat{\rho}[\tau'] / \hat{\Phi}[\tau]$; ▷ For censored waiting times on chords

  $\hat{\rho}^\dagger[\cdot] \leftarrow \hat{\rho}^\dagger[\cdot] \otimes K_h[\cdot]$; ▷ Regularization implemented by convolution with the kernel smoother

  $Z \leftarrow \sum_\tau \hat{\rho}^\dagger[\tau]$; $\hat{\rho}^\dagger[\cdot] \leftarrow \hat{\rho}^\dagger[\cdot]/Z$; ▷ The newly estimated WTD is normalized to sum to unity

  $\hat{\rho}'[\cdot] \leftarrow \hat{\rho}[\cdot]$; $\hat{\rho}[\cdot] \leftarrow \hat{\rho}^\dagger[\cdot]$; ▷ Record and update the current WTD estimate

until $\|\hat{\rho}, \hat{\rho}'\|_{\mathrm{KS}} < \epsilon$; ▷ The error threshold used for judging the convergence of iterations is $\epsilon = 10^{-4}$

return $\hat{\rho}[\cdot]$

Algorithm 7: Computation of Bootstrap Confidence Band

Input: $\hat{\mathcal{G}}$, $\hat{\rho}[\cdot]$, D ▷ Estimated underlying network, WTD, and original observed DAT cascades

B, α ▷ Bootstrap resample size and confidence level

Output: $\hat{\rho}_{\mathrm{lower}}[\cdot]$, $\hat{\rho}_{\mathrm{upper}}[\cdot]$ ▷ The lower and upper bands of $\hat{\rho}[\cdot]$ with $1 - \alpha$ confidence

for $b \in \{1, \dots, B\}$ **do**

  for $\mathcal{D}^i \in D$ **do**

    $s^i \leftarrow \arg\min_{v\in\mathcal{G}} t^i_v \in \mathcal{D}^i$; ▷ Find the source node for cascade $\mathcal{D}^i$

    $\tilde{\mathcal{D}}^i \leftarrow generative\_model(\hat{\mathcal{G}}, \hat{\rho}[\cdot], s^i)$; ▷ Resample DAT cascades with the same sources

  $(\tilde{\mathcal{G}}^b, \tilde{\rho}^b) \leftarrow STN\_inference(\{\tilde{\mathcal{D}}^i\})$; ▷ Inferred bootstrap STN tuple from the resmapled DAT data

for $\tau \in \{1, \dots, l_\tau\}$ **do** ▷ Calculate the pointwise α-level confidence interval for the estimated WTD

  $\hat{\rho}_{\mathrm{lower}}[\tau] \leftarrow \frac{\alpha}{2}$*-quantile of* $\{\tilde{\rho}^b[\tau]\}_{b\in\{1,\dots,B\}}$;

  $\hat{\rho}_{\mathrm{upper}}[\tau] \leftarrow (1 - \frac{\alpha}{2})$*-quantile of* $\{\tilde{\rho}^b[\tau]\}_{b\in\{1,\dots,B\}}$;

return $\hat{\rho}_{\mathrm{lower}}[\cdot]$, $\hat{\rho}_{\mathrm{upper}}[\cdot]$

Supplementary Note 8: Definitions of DSD, PCC and NMI

In this Supplementary Note, we provide detailed definitions of the three statistical quantities in Table 2 in the main text—distributional standard deviation (DSD), Pearson correlation coefficient (PCC), and normalised mutual information (NMI).

On one hand, to reflect the node heterogeneity in the underlying WTDs, we have calculated

$$\mathrm{DSD} = \sqrt{\frac{N}{N-1}\frac{1}{\pi_0}\sum_{u\in V}\pi_u\|\rho_u(\tau),\rho(\tau)\|^2_{\mathrm{KS}}}, \tag{39}$$

where $\rho_u(\tau) = (1/\pi_u)\sum_{(u,v)\in G}\pi_{uv}\rho_{uv}(\tau)$ is the average WTD over all node u's outgoing links weighted by $\pi_{uv} = \#\{[(w,u,t'),(u,v,t)] \in E^2 : (w,u,t') \prec (u,v,t)\}$ denoting the number of ordered pairs of successive temporal links, $(w,u,t') \prec (u,v,t)$, namely, $t' < t$ and $\forall t'' \in (t',t) : (u,v,t'') \notin E$ hold. Note that π_{uv} weighs the occurrence frequency of temporal links (u,v,t) appearing as the first routes for diffusion from node u to node v, and the prefactor $N/(N-1)$ in equation (39) comes from Bessel's correction since the empirical network data were sampled in a finite observation window $[0,T^w]$. Furthermore, $\rho(\tau) = (1/\pi_0)\sum_{u\in G}\pi_u\rho_u(\tau)$, with weights $\pi_u = \sum_{u\in V}\pi_{uv}$ and $\pi_0 = \sum_{u\in V}\pi_u$, represents the overall average (or mean-field) WTD across the entire population, which reads as the following empirical PDF of waiting times obtained from the raw temporal network data:

$$\rho(\tau) = \frac{1}{\pi_0}\sum_{(w,u,t')\in E}\ \sum_{\substack{(u,v,t)\in E:\\ (w,u,t')\prec(u,v,t)}}\delta(\tau-(t-t')), \tag{40}$$

where $\delta(\cdot)$ is the Dirac delta function.

Remark.—Instead of defining $\mathrm{DSD}^{\mathrm{link}} = \sqrt{\frac{L}{L-1}\frac{1}{\pi_0}\sum_{(u,v)\in G}\pi_{uv}\|\rho_{uv}(\tau),\rho(\tau)\|^2_{\mathrm{KS}}}$ at the link level, we used Eq.(39) to explore the node heterogeneity of empirical WTDs of temporal networks primarily for avoiding the problem of overfitting. Note that it is also natural to consider the average WTD across all outgoing links from a node, which corresponds to many realistic scenario where temporal links (e.g., phone calls and emails) are activated by the information senders. Besides, we make further discussion on our choice of weights π_{uv}. In contrast to the mean-field IET distribution, $\psi(\tau) = (1/L)\sum_{(u,v)\in E}\pi^{\mathrm{IET}}_{uv}\psi_{uv}(\tau)$, obtained by simply weighing the link-level $\psi_{uv}(\tau)$ with the corresponding link number $\pi^{\mathrm{IET}}_{uv} = \#\{(u,v,t) \in E\}$, we instead chosen, when averaging WTDs, the weight π_{uv} for each WTD $\rho_{uv}(\tau)$ as the the number of length-2, time-respecting paths ending with this link. This is natural from the definition of waiting times on such paths consisting of successive temporal links, which encapsulates the added temporal properties of the diffusion propagator in the context we considered here. As a straightforward consequence of this weight choice, the (frequency-weighted) average WTD is expressible as the empirical PDF of waiting times [see equation (40)]. Intuitively, such frequencies π_{uv} of links (u,v) to possibly be the first routes to spread the information also provide quantification of the importance centrality of (topological) links in the diffusion dynamics on temporal networks.

On the other hand, examination of inter-link dependencies underlying realistic WTDs is much more straightforward than that of the distributional identity. Here we adopted Pearson's correlation analysis to check for empirical associations between the waiting times on any pair of successive temporal links. More

specifically,

$$\text{PCC} = \frac{\sum(x_i - \bar{x})(y_i - \bar{y})}{\sqrt{\sum(x_i - \bar{x})^2}\sqrt{\sum(y_i - \bar{y})^2}}, \tag{41}$$

where $\{(x_i, y_i)\} = \{(t - t', t' - t'')|\exists[(s, w, t''), (w, u, t'), (u, v, t)] \in E^3 : (s, w, t'') \prec (w, u, t') \prec (u, v, t)\}$ represents the set of successive waiting times occurred on all possible time-respecting paths of length 3 in the empirical network, $\bar{x}$ and $\bar{y}$ are the means of $\{x_i\}$ and $\{y_i\}$, respectively.

Note that PCC only reflects precision of a linear tendency, to discover the most general dependence we further calculated the normalised mutual information between the observations $\{x_i\}$ and $\{y_i\}$:

$$\text{NMI} = \frac{\hat{I}(X, Y)}{\min[\hat{H}(X), \hat{H}(Y)]}, \tag{42}$$

where $\hat{H}(X), \hat{H}(Y)$ and $\hat{I}(X, Y)$ represents the estimated entropy and mutual information from the samples $\{x_i\}$, $\{y_i\}$ and $\{(x_i, y_i)\}$ [47], and thus NMI is normalised to $[0, 1]$ and describes the second-order (inter-link) dependency, ranging from mutual independence (NMI=0) to deterministic functional relationship (NMI=1) between the waiting times on any successive temporal links. Relevant statistical indices (DSD, PCC and NMI) of a variety of realistic temporal networks, as well as the performance of our inferential method applied to the computer-generated diffusion cascades using these empirical datasets are reported in Table 2 in the main text.

Supplementary Note 9: Extended STN model with second-order inter-link correlations

In this note we discuss the case of higher-order STNs allowing nonzero correlations between waiting times occurring on STN links. Here we focus on the second-order WTD, $\rho_{uv,vw}(\tau_1, \tau_2)$ which describes the joint probability that the waiting times for successive spreads of information on any two adjacent links $(u, v), (v, w) \in \mathcal{G}$ are τ_1, τ_2, respectively. We reformulate the likelihood function as

$$L(\mathcal{D}|\mathcal{N}_\mathrm{s}) = \textstyle\sum_{\mathcal{T}\subset\mathcal{G}} L(\mathcal{D}, \mathcal{T}|\mathcal{N}_\mathrm{s}) = \displaystyle\sum_{\mathcal{T}\subset\mathcal{G}} \prod_{\substack{(v,w)\in\mathcal{T}\\(u,v)\in\mathcal{T}}} \rho_{vw|uv}(d_{vw}|d_{uv}) \prod_{\substack{(v,w)\notin\mathcal{T}\\(u,v)\in\mathcal{T}}} \Phi_{vw|uv}(d_{vw}|d_{uv}) \tag{43}$$

where link (u, v) denotes the branch of diffusion tree $\mathcal{T}$ successively followed by link (v, w), and $\rho_{vw|uv}(\tau_2|\tau_1) = \rho_{uv,vw}(\tau_1, \tau_2)/\int_0^\infty \rho_{uv,vw}(\tau_1, \tau)d\tau$ and $\Phi_{vw|uv}(\tau_2|\tau_1) = \int_{\tau_2}^\infty \rho_{vw|uv}(\tau|\tau_1)d\tau$ are the conditional PDF and survival function of the second-order WTD $\rho_{uv,vw}(\tau_1, \tau_2)$, respectively. In particular when $v = s^*$ is the information source of the diffusion, we use the notation $\rho_{vw|\varnothing}(\tau|\varnothing) = \rho_{vw}(\tau)$ to present an ordinary WTD defined under the first-order null model assumption.

We consider the marginal gain $\Delta_{uv}\ell(D|\mathcal{G}, \rho)$ of flipping a single link (u, v) similarly defined as the logarithmic likelihood ratio between the proposed and current network configurations [see equation (6) in the main text]. The MCMC sampling procedure for the topology inference is still valid, but becomes extremely computation-intensive, mainly because calculating the likelihood function requires an enumeration of all possible diffusion trees compatible with the observed diffusion cascade. Note that the Weighted Matrix-Tree Theorem does not hold under the case of inter-link correlations.

Next we provide the recipe for estimating $\rho_{vw|uv}(\tau_2|\tau_1)$. We first introduce the second-order branching coefficients with respect to successive links $(u, v), (v, w) \in \mathcal{G}$ for the following two distinct cases:

$$\begin{aligned}
\beta_{uv,vw}(\mathcal{D}, \mathcal{N}_\mathrm{s}) &= \Pr\left[(u, v) \in \mathcal{T}, (v, w) \in \mathcal{T}|\mathcal{D}, \mathcal{N}_\mathrm{s}\right] = \frac{\left[L - L_{\{uv\}} - L_{\{vw\}} + L_{\{uv,vw\}}\right](\mathcal{D}|\mathcal{N}_\mathrm{s})}{L(\mathcal{D}|\mathcal{N}_\mathrm{s})}, \\
\beta_{uv,\overline{vw}}(\mathcal{D}, \mathcal{N}_\mathrm{s}) &= \Pr\left[(u, v) \in \mathcal{T}, (v, w) \notin \mathcal{T}|\mathcal{D}, \mathcal{N}_\mathrm{s}\right] = \frac{\left[L_{\{vw\}} - L_{\{uv,vw\}}\right](\mathcal{D}|\mathcal{N}_\mathrm{s})}{L(\mathcal{D}|\mathcal{N}_\mathrm{s})},
\end{aligned} \tag{44}$$

where $L_\mathcal{A}(\mathcal{D}|\mathcal{N}_\mathrm{s})$ is the likelihood of observing the diffusion cascade $\mathcal{D}$ on the STN $\mathcal{N}_\mathrm{s}$ given the taboo set $\mathcal{A}$ of links acting as cavity chords, i.e.,

$$L_\mathcal{A}(\mathcal{D}|\mathcal{N}_\mathrm{s}) = \sum_{\substack{\mathcal{T}\subset\mathcal{G}\\ \mathcal{T}\cap\mathcal{A}=\varnothing}} L(\mathcal{D}, \mathcal{T}|\mathcal{N}_\mathrm{s}). \tag{45}$$

Therefore, the conditional WTDs $\boldsymbol{\rho} = \left[\rho_{vw|uv}(\tau_2|\tau_1)\right]_{(u,v),(v,w)\in\mathcal{E}}$ can be estimated via a similar iterative procedure from the following self-consistent equations:

$$\begin{aligned}
\hat{\rho}_{vw|uv}(\tau_2|\tau_1) = \frac{1}{\sum_{a=1}^C K_h(\tau_1 - d^a_{uv})} \sum_{a=1}^C K_h(\tau_1 - d^a_{uv}) \Big\{ &\beta_{vw|uv}(\mathcal{D}^a, \mathcal{G}, \hat{\boldsymbol{\rho}}) K_h(\tau_2 - d^a_{vw}) + \\
&\left[1 - \beta_{vw|uv}(\mathcal{D}^a, \mathcal{G}, \hat{\boldsymbol{\rho}})\right] \frac{\hat{\rho}_{vw|uv}(\tau_2|\tau_1)\Theta(\tau_2 - d^a_{vw})}{\Phi_{vw|uv}(d^a_{vw}|\tau_1)} \otimes K_h(\tau_2) \Big\},
\end{aligned} \tag{46}$$

with the conditional branching coefficient $\beta_{vw|uv}(\mathcal{D},\mathcal{N}_{\rm s}) = \Pr\left[(v,w)\in\mathcal{T}\middle|(u,v)\in\mathcal{T},\mathcal{D},\mathcal{N}_{\rm s}\right]$ given by

$$\beta_{vw|uv}(\mathcal{D},\mathcal{N}_{\rm s}) = \frac{\beta_{uv,vw}(\mathcal{D},\mathcal{N}_{\rm s})}{\beta_{uv,vw}(\mathcal{D},\mathcal{N}_{\rm s})+\beta_{uv,\overline{vw}}(\mathcal{D},\mathcal{N}_{\rm s})} = 1-\frac{\left[L_{\{vw\}}-L_{\{uv,vw\}}\right](\mathcal{D}|\mathcal{N}_{\rm s})}{\left[L-L_{\{uv\}}\right](\mathcal{D}|\mathcal{N}_{\rm s})}. \tag{47}$$

Figure 22 shows the estimated second-order WTDs using synthesised diffusion cascades on an illustrative network consisting of seven nodes, which verifies the consistency of our proposed iterative method.

We further check the validation of the following pairwise approximate relation between the first- and second-order branching coefficients:

$$\beta_{uv,vw}(\mathcal{D},\mathcal{N}_{\rm s}) \doteq \beta_{uv}(\mathcal{D},\mathcal{N}_{\rm s})\beta_{vw}(\mathcal{D},\mathcal{N}_{\rm s}). \tag{48}$$

As shown in Supplementary Figure 22(e), numerical results imply that such an approximation holds well for those central links with relatively large branching coefficients, in spite of increased deviations for peripheral links with relatively small branching coefficients. It should be noted that in the second-order case, $\beta_{uv}(\mathcal{D},\mathcal{N}_{\rm s})$ is calculated using the following formula

$$\beta_{uv}(\mathcal{D},\mathcal{N}_{\rm s}) = \frac{\left[L-L_{\{uv\}}\right](\mathcal{D}|\mathcal{N}_{\rm s})}{L(\mathcal{D}|\mathcal{N}_{\rm s})}, \tag{49}$$

and that equation (14) usually becomes incorrect in the presence of polyadic dependencies.

Supplementary Note 10: Effect of discretisation size and Fourier-domain WTD estimator

Effect of discretisation size.—In computer simulations, we adopt the discretised WTDs with binning-based discretisation size δt. It is clear that in the case of discretised waiting times, it is possible for an individual to have "tied informers" with exactly simultaneous arrival times, as illustrated in Supplementary Figure 2. Here, we do not explicitly distinguish between discretisation widths and event durations because they produce the identical effect of finite temporal resolution and hence destroy the uniqueness property of branches. We denote by $\beta_{uv}[\mathcal{D}, \mathcal{N}_\mathrm{s}]$ the probability of link (u, v) being a single or one of multiple tied diffusion branches during the observed cascade. We next show that the discretised version of branching coefficient is written

$$\beta_{uv}[\mathcal{D}, \mathcal{N}_\mathrm{s}] = \frac{\lambda_{uv}[d_{uv}]}{\Lambda_v[\mathcal{I}_v]}, \tag{50}$$

with $\lambda_{uv}[\cdot]$ denoting the discretised hazard function and $\Lambda_v[\mathcal{I}_v]$ recursively given by

$$\Lambda_v[\varnothing] = 0, \qquad \Lambda_v[\mathcal{I}_v] = \big(1 - \lambda_{uv}[d_{uv}]\delta t\big)\Lambda_v[\mathcal{I}_v \setminus u] + \lambda_{uv}[d_{uv}], \quad \forall u \in \mathcal{I}_v. \tag{51}$$

We first show that $\Lambda_v[\mathcal{I}_v]$ is well-defined. It suffices to verify that for distinguished neighbours $u, u' \in \mathcal{I}_v$,

$$\begin{aligned}
&\big(1 - \lambda_{uv}[d_{uv}]\delta t\big)\Lambda_v(\mathcal{I}_v \setminus u) + \lambda_{uv}[d_{uv}] \\
&\quad = \big(1 - \lambda_{uv}[d_{uv}]\delta t\big)\Big\{\big(1 - \lambda_{u'v}[d_{u'v}]\delta t\big)\Lambda_v(\mathcal{I}_v \setminus \{u, u'\}) + \lambda_{u'v}[d_{u'v}]\Big\} + \lambda_{uv}[d_{uv}] \\
&\quad = \big(1 - \lambda_{u'v}[d_{u'v}]\delta t\big)\Big\{\big(1 - \lambda_{uv}[d_{uv}]\delta t\big)\Lambda_v(\mathcal{I}_v \setminus \{u, u'\}) + \lambda_{uv}[d_{uv}]\Big\} + \lambda_{u'v}[d_{u'v}] \\
&\quad = \big(1 - \lambda_{u'v}[d_{u'v}]\delta t\big)\Lambda_v(\mathcal{I}_v \setminus u') + \lambda_{u'v}[d_{u'v}],
\end{aligned} \tag{52}$$

and that $\Lambda_v[\mathcal{I}_v] = \lambda_{uv}[d_{uv}]$ for the local configuration $\mathcal{I}_v$ consisting of only a single incident link (u, v).

Next we prove equation (50). From the inclusion-exclusion principle, the conditional probability of observing DAT t_v at the focus node v given the local information $\{t_u\}_{u\in\mathcal{I}_v}$ is expressed as

$$\begin{aligned}
&p\Big(T_v = t_v \Big| \{T_u = t_u\}_{u\in\mathcal{I}_v}\Big)\delta t \\
&= \sum_{u\in\mathcal{I}_v} \rho_{uv}[d_{uv}]\delta t \prod_{u'\neq u} \Phi_{u'v}[d_{u'v}] - \sum_{u,u'\in\mathcal{I}_v} \rho_{uv}[d_{uv}]\rho_{u'v}[d_{u'v}]\delta t^2 \prod_{u''\neq u,u'} \Phi_{u''v}[d_{u''v}] + \cdots - (-1)^{\#\mathcal{I}_v} \prod_{u\in\mathcal{I}_v} \rho_{u'v}[d_{u'v}]\delta t \\
&= \Bigg\{ \sum_{u\in\mathcal{I}_v} \lambda_{uv}[d_{uv}]\delta t - \sum_{u,u'\in\mathcal{I}_v} \lambda_{uv}[d_{uv}]\lambda_{u'v}[d_{u'v}]\delta t^2 + \cdots - (-1)^{\#\mathcal{I}_v} \prod_{u'\in\mathcal{I}_v} \lambda_{u'v}[d_{u'v}]\delta t \Bigg\} \prod_{u\in\mathcal{I}_v} \Phi_{uv}[d_{uv}] \\
&= \Bigg\{ \lambda_{uv}[d_{wv}]\delta t + \big(1 - \lambda_{uv}[d_{wv}]\delta t\big)\Big[\sum_{u'\in\mathcal{I}_v\setminus w} \lambda_{u'v}[d_{u'v}]\delta t - \cdots - (-1)^{\#\mathcal{I}_v\setminus w} \prod_{u'\in\mathcal{I}_v\setminus w} \lambda_{u'v}[d_{u'v}]\delta t \Big] \Bigg\} \prod_{u\in\mathcal{I}_v} \Phi_{uv}[d_{uv}].
\end{aligned} \tag{53}$$

By checking the recursive relation equation (51), one can readily verify that the curly braced term is exactly $\Lambda_v[\mathcal{I}_v]\delta t$, which thus gives

$$p\Big(T_v = t_v \Big| \{T_u = t_u\}_{u\in\mathcal{I}_v}\Big) = \Lambda_v[\mathcal{I}_v] \prod_{u\in\mathcal{I}_v} \Phi[d_{uv}]. \tag{54}$$

Similarly we first calculate the complementary probability of the branching coefficient, $\bar{\beta}_{uv}[\mathcal{D}, \mathcal{N}_\mathrm{s}] =$

$L(\mathcal{D}|\mathcal{G}\setminus uv,\rho)\Pr[\tau_{uv}>d_{uv}]/L(\mathcal{D}|\mathcal{G},\rho)$, and hence

$$\begin{aligned}\beta_{uv}[\mathcal{D},\mathcal{N}_{\mathrm{s}}] &= 1-\frac{p\Big(T_v=t_v\Big|\{T_{u'}=t_{u'}\}_{u'\in\mathcal{I}_v\setminus u}\Big)\Big(\Phi[d_{uv}]-\rho[d_{uv}]\delta t\Big)}{p\Big(T_v=t_v\Big|\{T_u=t_u\}_{u\in\mathcal{I}_v}\Big)} \\ &= 1-\frac{\Lambda_v[\mathcal{I}_v\setminus u]\Big(1-\lambda_{uv}[d_{uv}]\delta t\Big)}{\Lambda_v[\mathcal{I}_v]}=\frac{\lambda_{uv}[d_{uv}]}{\Lambda_v[\mathcal{I}_v]}.\end{aligned} \tag{55}$$

Here we used the recursive relation of $\Lambda_v[\mathcal{I}_v]$ in the last equality, thus completing the proof of equation (50).

Note that such branching coefficients under discrete WTDs violate the normalization condition, and clearly we have $\sum_{u\in\mathcal{I}_v}\beta_{uv}(\mathcal{D},\mathcal{G},\rho)>1$, if permitting simultaneous occurrence of multiple branches from tied informers during the diffusion cascade. Straightforwardly, one also sees from Supplementary Figure 2 that the branches constitute an acyclic diffusion tree with likelihood $1-O(\delta t)$. Note that in the continuum limit ($\delta t\to 0$), equation (50) simplifies into equation (14), and the updates for maintaining $\Lambda_v(\mathcal{D}|\mathcal{G},\rho)$ in Algorithm 5 reduce to $\Lambda_v\leftarrow\Lambda_v\pm\lambda_{uv}[d_{uv}]$ when flipping link (u,v) from the current configuration $\mathcal{G}$.

Fourier-domain WTD estimator.—Similar to the role played by the MCMC iteration number M in topology inference, the discretisation size δt affects both inferential precision and algorithmic complexity of the WTD estimator. Straightforwardly, the implementation with bin width δt has twice number of parameters to be learned (i.e., the probability masses $\rho[i]\delta t=\int_{i\delta t}^{(i+1)\delta t}\rho(\tau')d\tau', i=0,1,\ldots,\lceil l_\tau/\delta t\rceil$) and thus about four times computation complexity than the WTD estimator with bin width $\delta t'=2\delta t$. Motivated by this, a promising speedup of our algorithm by reducing the parameter space is to perform the transform from time-domain estimation to Fourier-domain.

Here we illustrate the Fourier-domain estimation using truncated Fourier series (up to the K-th order) to approximate the underlying WTDs:

$$\rho_{uv}^K(\tau)=\sum_{n=-K}^{K}c_{uv}^n e^{in\pi\tau/l_\tau}, c_{uv}^n=\frac{1}{2l_\tau}\int_{-l_\tau}^{l_\tau}\rho_{uv}(\tau)e^{-in\pi\tau/l_\tau}d\tau, \tag{56}$$

which is estimated as $\hat{\rho}^K(\tau)=\sum_{n=-K}^{K}\hat{c}_n e^{in\pi\tau/l_\tau}$ with the Fourier series coefficients $\{\hat{c}_{uv}^n\}$ given by

$$\hat{c}_{uv}^n=\frac{1}{C}\sum_{i=1}^{C}k_h^n\left\{\beta_{uv}(\mathcal{D}^i,\mathcal{G},\hat{\boldsymbol{\rho}})e^{-in\pi d_{uv}^i/l_\tau}+\Big[1-\beta_{uv}(\mathcal{D}^i,\mathcal{G},\hat{\boldsymbol{\rho}})\Big]\frac{1}{2l_\tau}\int_{d_{uv}^i}^{l_\tau}\frac{\hat{\rho}_{uv}(\tau)}{\hat{\Phi}_{uv}(d_{uv}^i)}e^{-in\pi\tau/l_\tau}d\tau\right\}, \tag{57}$$

where l_τ is the support of WTD $\rho(\tau)$, and k_n is the n-th Fourier series coefficient of the kernel smoother, $K_h(\tau)=\sum_{n=-K}^{K}k_h^n e^{in\pi\tau/l_\tau}$. In particular, in the limit case of vanishing kernel width [$K_h(\tau)\to\delta(\tau)$, as $h\to 0$], the Fourier series coefficients reduce to $k_h^n=1$.

We note here that the integrand in the last term of equation (22) is a complex exponential function and hence readily evaluable, ultimately leading to a linear combination of $\{\hat{c}_{uv}^n\}$, by which we have established a set of self-consistency equations with respect to $\{\hat{c}_{uv}^n\}$ for the iterative WTD estimator. Furthermore, by introducing the even (or odd) extension of WTD ρ_{uv}, one can further use Fourier sine (or cosine) series to simplify the computation. Note that the former cases implies that $\hat{\rho}_{uv}^K(0)=0$, while the latter imposes no such restrictions.

Thanks to this radically reduced number of parameters, the convergence rate of self-consistency iterations is substantially improved compared to the time-domain estimator. Note that this is at the cost of relaxing the nonnegativity constraint of WTDs, and there arises another problem that requires $\hat{\rho}^K(\tau)$ to be "bona fide" density [45]. In other words, $\hat{\rho}^K(\tau)$ has to be the PDF of a real distribution that is normalized to one and $\hat{\rho}^K(\tau) \geq 0$ holds on its domain. Conventionally, additional processing steps (e.g., smoothing, truncation and rescaling) are needed for such non-bona fide density estimator to fix the nonnegativity constraint violation.

Note that there are two important affecting parameters. The first is the order K of the truncated Fourier series used for inference, which determines a similar tradeoff between accuracy and complexity as that encountered in the time-domain (see Supplementary Figure 23 for illustrative numerical examples). The second is the WTD support l_τ. Obviously, using an underestimated value results in loss of distributional information. A conservative choice is equating l_τ to the length T^w of the observation window, or the maximum timespan of recorded diffusion cascades. However, such overestimated values may cause the overfitting and/or convergence problems. In fact, there is an abundant and fruitful literature relating to the so-called "support estimation" problem, particular for high-dimensional distributions [46]. However, finding efficient data-driven methods for support estimation using indirect measurements of collective dynamics is a desirable and challenging task. This issue is out of our scope and not directly related to other parts of the present paper. For simplicity, we assume l_τ to be known in our numerical experiments.

Remark.—As a final remark, the binned WTD $\rho[i]$ is rewritten as $\langle \rho, b_i \rangle = \int_0^{l_\tau} \rho(\tau')b_i(\tau')d\tau'$, the inner product of WTD $\rho(\tau)$ and the i-th standard orthogonal basis $b_i(\tau) = (1/\delta t)\mathbb{1}_{[\tau \in [i\delta t, (i+1)\delta t)]}$ of the "binning" operation on WTD $\rho(\tau)$. Therefore the binned WTD $\rho[i]$ also constitutes a generalized Fourier series of $\rho(\tau)$. In fact, our inferential framework is readily extended to any generalized Fourier series representation, ranging from Fourier-Bessel or other orthogonal polynomial series expansions to wavelet domain. A consequent question is which series performs the best for the purpose of density estimation. Here we leave as an open problem the optimal orthogonal series estimates that minimise the predictive error, in particular for the empirically relevant power-law distributions exhibiting marked anomalous patterns such as bursts and heavy tails.

Supplementary Note 11: Derivation of the entropic complexity metric $\xi(\mathcal{D},\mathcal{N}_\mathrm{s})$

Let us first elucidate the information-theoretic meaning of $\xi(\mathcal{D},\mathcal{N}_\mathrm{s})$ [equation (8) in the main text]. In the literature of algebraic graph theory, the complexity $\xi(\mathcal{G})$ of a static network $\mathcal{G}$, is defined as the (logarithmic) number of its spanning trees, which reflects the path variability in the connectedness between network nodes. Here, we extendedly consider the complexity $\xi(\mathcal{D},\mathcal{N}_\mathrm{s})$ of temporal network $\mathcal{N}_\mathrm{s}$ as the uncertainty of potential diffusion trees associated with the observed cascade that takes place on them. Let $p_\mathcal{G} = \left\{p_\mathcal{T} \triangleq p(\mathcal{T}|\mathcal{D},\mathcal{N}_\mathrm{s})\right\}_{\mathcal{T}\subset\mathcal{G}}$ be any probability mass function over all possible diffusion trees $\mathcal{T}$ for the diffusion cascade $\mathcal{D}$ on $\mathcal{N}_\mathrm{s}$. Applying Jensen's inequality to the log-likelihood function gives

$$\ell(\mathcal{D}|\mathcal{N}_\mathrm{s}) = \log\sum_{\mathcal{T}\subset\mathcal{G}} L(\mathcal{D},\mathcal{T}|\mathcal{N}_\mathrm{s}) \geq \sum_{\mathcal{T}\subset\mathcal{G}} p_\mathcal{T}\log\frac{L(\mathcal{D},\mathcal{T}|\mathcal{N}_\mathrm{s})}{p_\mathcal{T}}, \tag{58}$$

and the exact equality is achieved when $p^*_\mathcal{T} \propto L(\mathcal{D},\mathcal{T}|\mathcal{N}_\mathrm{s})$ as the likelihood weighting of diffusion trees. Thus substituting Eqs. (4) and (14) yields

$$\begin{aligned} p^*_\mathcal{T} &= \frac{L(\mathcal{D},\mathcal{T}|\mathcal{N}_\mathrm{s})}{L(\mathcal{D}|\mathcal{N}_\mathrm{s})} = \frac{\prod_{(u,v)\in\mathcal{T}}\rho_{uv}(d_{uv})\prod_{(u,v)\notin\mathcal{T}}\Phi_{uv}(d_{uv})}{\prod_{v\in\mathcal{V}\backslash s^*}\Lambda_v(\mathcal{D}|\mathcal{N}_\mathrm{s})\prod_{(u,v)\in\mathcal{G}}\Phi_{uv}(d_{uv})} \\ &= \prod_{(u,v)\in\mathcal{T}}\frac{\lambda_{uv}(d_{uv})}{\Lambda_v(\mathcal{D}|\mathcal{N}_\mathrm{s})} = \prod_{(u,v)\in\mathcal{T}}\beta_{uv}(\mathcal{D},\mathcal{N}_\mathrm{s}). \end{aligned} \tag{59}$$

To quantify the indeterminacy of diffusion structure underlying the cascading process on networks, we define $\xi(\mathcal{D},\mathcal{N}_\mathrm{s}) = H\left[p^*_\mathcal{G}\right] = -\sum_{\mathcal{T}\subset\mathcal{G}} p^*_\mathcal{T}\log p^*_\mathcal{T}$ as the Shannon entropy of $p^*_\mathcal{G} = \left\{p^*_\mathcal{T}\right\}_{\mathcal{T}\subset\mathcal{G}}$. By exploiting uncorrelated properties of STN links, we can effectively express $\xi(\mathcal{D},\mathcal{N}_\mathrm{s})$ in terms of entropies of branching coefficients

$$\xi(\mathcal{D},\mathcal{N}_\mathrm{s}) = \sum_{v\in\mathcal{V}\backslash s^*} H\left[\left\{\beta_{uv}(\mathcal{D},\mathcal{N}_\mathrm{s})\right\}_{u\in\mathcal{I}_v}\right] = -\sum_{(u,v)\in\mathcal{G}}\beta_{uv}(\mathcal{D},\mathcal{N}_\mathrm{s})\log\beta_{uv}(\mathcal{D},\mathcal{N}_\mathrm{s}), \tag{60}$$

which is equivalent to equation (8) in the main text.

We next prove equation (60). Let $\mathcal{T}_{uv} = \left\{\mathcal{T}\subset\mathcal{G}\colon (u,v)\in\mathcal{T}\right\}$ be the set of diffusion trees such that a specific branch (u,v) is included. Let $v = \arg\max_{u\in\mathcal{V}}\{t_u\in\mathcal{D}\}$ be the latest informed node in the given cascade $\mathcal{D}$, and thus $\mathscr{T}_v = \left\{\mathcal{T}_{uv}\right\}_{u\in\mathcal{I}_v}$ constitutes a partition of all possible diffusion trees. From the composition law of the entropy [40], it follows that

$$\xi(\mathcal{D},\mathcal{N}_\mathrm{s}) = H\left[p^*_\mathcal{G}\right] = H\left[\left\{p^*_{\mathcal{T}_{uv}}\right\}_{\mathcal{T}_{uv}\in\mathscr{T}_v}\right] + \sum_{\mathcal{T}_{uv}\in\mathscr{T}_v} p^*_{\mathcal{T}_{uv}} H\left[\left\{p^*_\mathcal{T}/p^*_{\mathcal{T}_{uv}}\right\}_{\mathcal{T}\in\mathcal{T}_{uv}}\right], \tag{61}$$

where $p^*_{\mathcal{T}_{uv}}$ is the partition weight assigned to $\mathcal{T}_{uv}$. By the definition of the branching coefficient, we immediately have

$$p^*_{\mathcal{T}_{uv}} = \sum_{\mathcal{T}\in\mathcal{T}_{uv}} p^*_\mathcal{T} = \Pr\left[(u,v)\text{ is a branch given }\mathcal{D}\text{ on }\mathcal{N}_\mathrm{s}\right] = \beta_{uv}(\mathcal{D},\mathcal{N}_\mathrm{s}). \tag{62}$$

Note that $p^*_\mathcal{T}/p^*_{\mathcal{T}_{uv}} = \prod_{(w,u)\in\mathcal{T}\backslash v}\beta_{wu}(\mathcal{D},\mathcal{N}_\mathrm{s})$, which is exactly the probability mass placed on the corresponding diffusion tree, $\mathcal{T}\setminus v$, contained in the subgraph obtained by deleting v from $\mathcal{G}$. The above equation can thus

be compactly rewritten as

$$\xi(\mathcal{D},\mathcal{N}_{\rm s}) = H\big[p^*_{\mathcal{G}}\big] = H\Big[\big\{\beta_{uv}(\mathcal{D},\mathcal{N}_{\rm s})\big\}_{u\in\mathcal{I}_v}\Big] + H\big[p^*_{\mathcal{G}\setminus v}\big]. \tag{63}$$

Repeating this procedure recursively, we finally obtain equation (60).

Remark.—Finally, it is noteworthy that for the more general case of high-order STNs with inter-link correlations (see Supplementary Note 9), we have

$$\xi(\mathcal{D},\mathcal{N}_{\rm s}) \leq -\sum_{(u,v)\in\mathcal{G}} \beta_{uv}(\mathcal{D},\mathcal{N}_{\rm s}) \log \beta_{uv}(\mathcal{D},\mathcal{N}_{\rm s}), \tag{64}$$

where the inequality follows from non-negativity of mutual information, implying that the sum of entropies of branching probabilities over incident links of respective nodes provides an upper bound on the complexity of the diffusion structure on general temporal networks.

Supplementary Note 12: MAP topology inference with the ℓ_1 sparsity penalty

Note that in subcritical inferential cases, the failure of WTD recovery from insufficient amounts of data usually arises from redundant links in the erroneously inferred time-aggregated topologies (see Supplementary Figure 6), rather than being limited by the density estimation via kernel methods *per se*. An intuition behind this observation is as follows. A single diffusion cascade $\mathcal{D}$ provides primarily two aspects of information: first, it reflects the probability or propensity of actual links underlying any node pair (u, v) with TDOA $d_{uv} \in (0, l_\tau]$; and second, it indicates definitely null connections between (u, v) such that $d_{uv} \in (l_\tau, \infty)$. Here, l_τ denotes the support of the WTD ρ. In general, the smaller the sample size is, the less null connections are detectable. Thus the topology inference from insufficient observed data can seriously result in Type I errors, as illustrated in Supplementary Figure 6. This is also clear from equation (16). In the equivalent utility maximisation problem (see Supplementary Note 3), the cost of establishing link (u, v) in the ML configuration $\hat{\mathcal{G}}$ takes the form $c_{uv} = -\sum_{i=1}^{C} \log \Phi(d_{uv}^i)$, which monotonically increases with the sample size C. We next show that introducing the Bayesian approach with properly selected sparsity priors over network configurations can significantly alleviate this issue.

To improve the reconstruction performance, we apply prior distributions to encourage sparse configurations of the time-aggregated topology $\mathcal{G}$. More concretely, we assign a Laplace prior of the form $\pi(\mathcal{G}) \propto e^{-\beta\|\mathcal{G}\|_1}$, which postulates an exponential family over the configuration space of the latent network. Here, $\|\cdot\|_1$ denotes the ℓ_1-norm (i.e., $\|\mathcal{G}\|_1$ represents the link number of $\mathcal{G}$), and β is a nonnegative hyperparameter controlling the amount of penalty for configurations with dense links. This is equivalent to introducing an extra cost β for establishing links, leading to $c_{uv} = -\sum_{i=1}^{C} \log \Phi(d_{uv}^i) + \beta$. We further test our inference algorithm using Laplace priors with different hyperparameters β, finding that imposing sparsity priors can significantly improve the reconstruction accuracy. To achieve a tradeoff between Type I and Type II errors occurred in the result of topology inference, there exists an optimal range for the values of hyperparameter β, as shown in Supplementary Figure 31. Despite the markedly improved reconstruction performance, we used flat and noninformative priors (namely, $\beta = 0$) in the main text for avoidance of nuisance parameters.

Supplementary References

[1] Lawless, J. F. *Statistical models and methods for lifetime data*, (John Wiley & Sons, 2011).

[2] Gautreau, A., Barrat, A. & Barthelemy, M. Global disease spread: statistics and estimation of arrival times. *J. Theor. Biol.* **251**, 509–522 (2008).

[3] Gelman, A., Carlin, J. B., Stern, H. S. & Rubin, D. B. *Bayesian data analysis*, (Chapman & Hall/CRC Boca Raton, FL, 2014).

[4] Barabási, A.-L. The origin of bursts and heavy tails in human dynamics. *Nature (London)* **435**, 207–211 (2005).

[5] Bailey, N. T. *The mathematical theory of infectious diseases and its applications* (Hafner Press, NY, 1975).

[6] Sierpinski, M. Sur une courbe dont tout point est un point de ramification. *Comptes Rendus (Paris)* **160**, 302–305 (1915).

[7] Dorogovtsev, S. N., Goltsev, A. & Mendes, J. F. F. Pseudofractal scale-free web. *Phys. Rev. E* **65**, 066122 (2002).

[8] Andrade Jr, J. S., Herrmann, H. J., Andrade, R. F. & Da Silva, L. R. Apollonian networks: Simultaneously scale-free, small world, euclidean, space filling, and with matching graphs. *Phys. Rev. Lett.* **94**, 018702 (2005).

[9] Erdős, P. & Rényi, A. On random graphs i. *Publ. Math. Debrecen* **6**, 290–297 (1959).

[10] Watts, D. J. & Strogatz, S. H. Collective dynamics of 'small-world' networks. *Nature (London)* **393**, 440–442 (1998).

[11] Barabási, A.-L. & Albert, R. Emergence of scaling in random networks. *Science* **286**, 509–512 (1999).

[12] Zachary, W. W. An information flow model for conflict and fission in small groups. *J. Anthropol. Res.* **33**, 452–473 (1977).

[13] Lusseau, D. *et al.* The bottlenose dolphin community of doubtful sound features a large proportion of long-lasting associations. *Behav. Ecol. Sociobiol.* **54**, 396–405 (2003).

[14] Knuth, D. E. *The Stanford GraphBase: A Platform for Combinatorial Computing* (Addison-Wesley, NY, 1993).

[15] Girvan, M. & Newman, M. E. J. Community structure in social and biological networks. *Proc. Natl. Acad. Sci. USA* **99**, 7821–7826 (2002).

[16] Gleiser, P. M. & Danon, L. Community structure in jazz. *Adv. Complex Syst.* **6**, 565–573 (2003).

[17] Vanhems, P. *et al.* Estimating potential infection transmission routes in hospital wards using wearable proximity sensors. *PLoS ONE* **8**(9), e73970 (2003).

[18] Génois, P. *et al.* Data on face-to-face contacts in an office building suggest a low-cost vaccination strategy based on community linkers. *Network Science* **3**, 326–347 (2015).

[19] Isella, L. *et al.* What's in a crowd? Analysis of face-to-face behavioral networks. *J. Theor. Biol.* **271**, 166–180 (2011).

[20] Stehlé, J. *et al.* High-resolution measurements of face-to-face contact patterns in a primary school. *PLoS ONE* **6**(8), e23176 (2011).

[21] Mastrandrea, R., Fournet, J. & Barrat, A. Contact patterns in a high school: a comparison between data collected using wearable sensors, contact diaries and friendship surveys. *PLoS ONE* **10**(9), e0136497 (2015).

[22] Timme, M. & Casadiego, J. Revealing networks from dynamics: an introduction. *J. Phys. A* **47**, 343001 (2014).

[23] Holme, P. & Saramäki (Eds.), J. *Temporal Networks* (Springer-Verlag, Berlin, 2013).

[24] Hanneke, S., Fu, W. & Xing, E. P. Discrete temporal models of social networks. *Electron. J. Stat.* **4**, 585–605 (2010).

[25] Crane, H. *et al.* Time-varying network models. *Bernoulli* **21**, 1670–1696 (2015).

[26] Kim, Y., Han, S., Choi, S. & Hwang, D. Inference of dynamic networks using time-course data. *Brief. Bioinform.* **15**, 212–228 (2014).

[27] Song, L., Kolar, M. & Xing, E. P. Keller: estimating time-varying interactions between genes. *Bioinformatics* **25**, i128–i136 (2009).

[28] Lebre, S., Becq, J., Devaux, F., Stumpf, M. P. & Lelandais, G. Statistical inference of the time-varying structure of gene-regulation networks. *BMC Sys. Biol.* **4**, 130 (2010).

[29] Kivelä, M. & Porter, M. A. Estimating interevent time distributions from finite observation periods in communication networks. *Phys. Rev. E* **92**, 052813 (2015).

[30] Kleinbaum, D. G. & Klein, M. *Survival analysis: a self-learning text* (Springer Science & Business Media, 2006).

[31] Shen, Z., Wang, W.-X., Fan, Y., Di, Z. & Lai, Y.-C. Reconstructing propagation networks with natural diversity and identifying hidden sources. *Nature Comm.* **5**, 4323 (2014).

[32] Han, X., Shen, Z., Wang, W.-X. & Di, Z. Robust reconstruction of complex networks from sparse data. *Phys. Rev. Lett.* **114**, 028701 (2015).

[33] Yang, X.-J. Exploring complex networks by walking on them. *Phys. Rev. E* **71**(1), 016107 (2005).

[34] Wand, M. P. & Jones, M. C. *Kernel Smoothing* (Chapman & Hall, UK, 1994).

[35] Duval, A., Klivans, C. & Martin, J. Simplicial matrix-tree theorems. *Trans. Am. Math. Soc.* **361**, 6073–6114 (2009).

[36] Papadimitriou, C. H. & Steiglitz, K. *Combinatorial optimization: algorithms and complexity* (Courier Corporation, 1982).

[37] Ahmed, S. & Atamtürk, A. Maximizing a class of submodular utility functions. *Math. Program., Ser. A* **128**, 149–169 (2011).

[38] Johnson, D. *Computers and intractability: a guide to the theory of NP-completeness.* (Freeman, San Francisco, CA, 1979).

[39] Krzanowski, W. J. & Hand, D. J. *ROC curves for continuous data* (Chapman & Hall/CRC Press, 2009).

[40] Jaynes, E. T. Information theory and statistical mechanics. *Phys. Rev.* **106**, 620 (1957).

[41] Kroese, D. P., Taimre, T. & Botev, Z. I. *Handbook of Monte Carlo methods*, (John Wiley & Sons, NJ, 2013).

[42] Kleinberg, J. & Tardos, E. *Algorithm design* (Pearson/Addison-Wesley, MA, 2006).

[43] Efron, B. Bootstrap methods: another look at the jackknife. *Annals of Statistics* **7**, 1–26 (1979).

[44] Efron, B. & Tibshirani, R. J. *Handbook of Monte Carlo methods*, (Chapman & Hall, CRC Press, 1993).

[45] Kronmal, R. & Tarter, M. The estimation of probability densities and cumulatives by Fourier series methods. *J. Amer. Statist. Assoc.* **63**(323), 925–952 (1968).

[46] Schölkopf, B. *et al.* Estimating the support of a high-dimensional distribution. *Neural Comput.* **13**(7), 1443–1471 (2001).

[47] Kraskov, A., bibinfoauthorStögbauer, H. & Grassberger, P. Estimating mutual information. *Phys. Rev., E* **69**(6), 066138 (2004).
